# Targeted NAT10 Degradation by PROTAC NP1192 Suppresses Hypoxia-Adaptive Glycolysis and Reinvigorates CD8^+^ Effector T‑Cell Function for Synergistic Cancer Immunotherapy

**DOI:** 10.1021/acscentsci.5c00812

**Published:** 2025-09-16

**Authors:** Keyi Ao, Zhiqiang Sun, Yi Hao, Jiaqi Qin, Chenglong Xu, Xiuli Wen, Zichao Yang, Li Li, Shaoyan Gan, Xiaona Chen, Xin Li, Jian Zhang, Jianjun Chen, Xia Guo

**Affiliations:** † Shenzhen Key Laboratory of Viral Oncology, Department of Science and Innovation, Shenzhen Hospital, 70570 Southern Medical University, Shenzhen, Guangdong 518100, P. R. China; ‡ Shenzhen School of Clinical Medicine, 70570 Southern Medical University, Shenzhen, Guangdong 510515, P. R. China; § Guangdong Provincial Key Laboratory of New Drug Screening, NMPA Key Laboratory for Research and Evaluation of Drug Metabolism, School of Pharmaceutical Sciences, 70570 Southern Medical University, Guangzhou, Guangdong 510515, P. R. China; ∥ Department of Ultrasound, South China Hospital, Medical School, Shenzhen University, Shenzhen, Guangdong 518116, P. R. China; ⊥ Department of Human Cell Biology and Genetics, School of Medicine, 255310 Southern University of Science and Technology, Shenzhen, Guangdong 518055, P. R. China; # Joint Laboratory of Guangdong−Hong Kong Universities for Vascular Homeostasis and Diseases, SUSTech Homeostatic Medicine Institute, School of Medicine, 255310 Southern University of Science and Technology, Shenzhen, Guangdong 518055, P. R. China; 7 Clinical Research Center, the First People’s Hospital of Foshan (The Affiliated Foshan Hospital of Southern University of Science and Technology), School of Medicine, 255310 Southern University of Science and Technology, Shenzhen, Guangdong 528000, P. R. China

## Abstract

Tumor resistance to immune checkpoint blockade (ICB) therapy is frequently driven by adaptive metabolic reprogramming in the hypoxic tumor microenvironment (TME). The key N4-acetylcytidine (ac4C) RNA modification mediator N-acetyltransferase 10 (NAT10) emerges as a promising therapeutic target, despite the lack of potent targeting agents. Here, we engineered NP1192, a PROTAC degrader targeting NAT10. NP1192 achieved nearly 70% NAT10 degradation and a 26.8% lower IC_50_ than canonical NAT10 inhibitor Remodelin in cervical cancer cells, outperforming Remodelin in antitumor effect *in vivo*, *in vitro*, and across three tumor organoids. It abrogated ac4C modifications on *HIF1A* mRNA and translation, reducing hypoxic lactate production and depleted ATP, and suppressed HIF-1α-mediated PD-L1 upregulation. In xenograft models, NP1192 combined with anti-PD-L1 inhibited subcutaneous xenograft growth and reduced tumor-core lactate gradients by > 80%. Furthermore, scRNA-seq and *in vitro* coculture experiments identified expansion of IFN-γ^+^ effector CD8^+^ T cells (T_eff_) and decline in exhausted CD8^+^ T cells (T_ex_). NP1192 in combination with anti-PD-L1 enhanced proliferation and effector function of CD8^+^ T_eff_ cells, thereby reversing resistance to anti-PD-L1 blockade therapy and synergizing with immunotherapy. These findings establish PROTAC-mediated NAT10 degradation as a dual metabolic-immune strategy to enhance checkpoint blockade efficacy.

## Graphical abstract

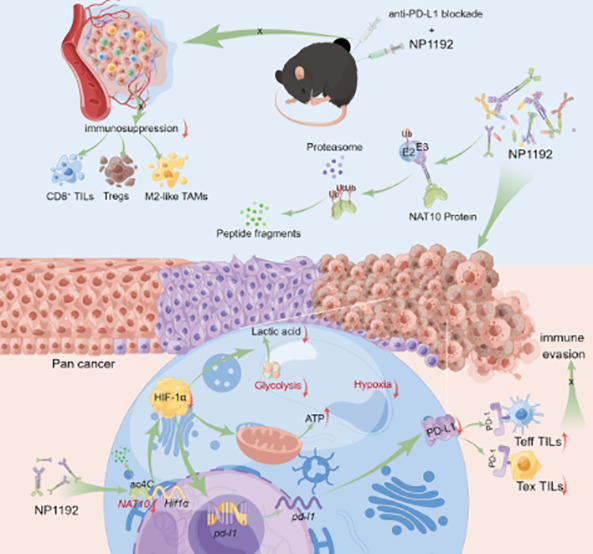

## Introduction

Advancements in understanding epigenetic regulatory mechanisms have accelerated the development of epigenetic-based antineoplastic agents to correct for aberrant epigenetic processes. Beyond the three FDA-approved classes of inhibitors,^1^ significant progress has been made in targeting posttranscriptional modifications. For example, FTO inhibitors, such as CS1 and carbon disulfide, disrupt mRNA methylation, exhibiting potent antileukemic effects and impairing cancer stem cell (CSC) self-renewal and immune evasion.^2^ Additionally, modulation of epigenetic alterations offers a promising cancer therapy strategy when combined with immunotherapy. For instance, decitabine enhances the antitumor immune response when combined with PD-1 inhibitors with exhausted CD8^+^ precursor T cells reversed.^3^ These advances in epigenetic modulators have deepened our understanding of how cancer cells exploit epigenetic mechanisms for survival.

N-Acetyltransferase 10 (NAT10), the key enzyme catalyzing N4-acetylcytidine (ac4C) modifications, stabilizes and enhances RNA translation efficiency. Overexpression of NAT10 has been linked to poor prognosis and aggressive tumor phenotypes in various malignancies.^4,5^ NAT10 interacts with mRNA transcripts of oncogenes like c-Myc and tumor suppressors like p53, promoting tumorigenesis.^6^ Additionally, NAT10 is involved in maintaining CSC self-renewal and survival.^7,8^ Our prior and other research in cervical cancer (CCa) demonstrated a correlation between elevated NAT10 expression and advanced CCa stage and metastasis, highlighting NAT10 as a potential therapeutic target in cancer management.^9,10^

Remodelin, a small-molecule inhibitor, suppresses NAT10 activity by binding to its catalytic site, causing structural changes that block RNA acetylation.^10^ However, its clinical application is limited by issues such as drug resistance, toxicity, and reduced efficacy,^11,12^ highlighting the need for optimization. In response, proteolysis-targeting chimeras (PROTACs) have emerged as a promising therapeutic approach. By using bifunctional molecules that simultaneously bind to an E3 ligase and a target protein, PROTACs facilitate ubiquitination and proteasomal degradation.^13–15^ This “event-driven” mechanism offers advantages like improved selectivity, overcoming resistance, and reducing off-target toxicity.^16^ PROTACs have shown efficacy against oncogenic proteins such as BRD4, NSD3, FOXM1, and STAT3, positioning them as promising candidates for modulating NAT10 in cancer therapy.^17–20^

In this study, a selective PROTAC degrader of NAT10, NP1192, is designed and proved to effectively reduce hypoxia-induced glycolysis, reverse CD8^+^ T cell dysfunction, and enhance anti-PD-L1 therapy, making it a promising cancer immunotherapeutic agent.

## Results

### Design and Synthesis of NAT10-Targeting PROTAC Degraders

PROTACs have garnered considerable attention in recent years as a cutting-edge technology for protein degradation since they have several advantages over conventional inhibitors, including their ability to overcome drug resistance and target previously “undruggable” proteins. PROTACs promote protein degradation through the ubiquitin–proteasome system (UPS) by recruiting an E3 ligase and the protein of interest (POI), facilitating the ubiquitination of the POI and subsequently triggering its recognition and degradation by the UPS system. We performed molecular modeling studies by docking a NAT10 inhibitor (Remodelin) into the human NAT10 protein structure downloaded from the AlphaFold protein structure database to design NAT10 PROTAC degraders. The docking poses are illustrated in Figure 1A–B. The benzene ring linked with the thiazole forms π–π-stacking interactions with Phe722. In addition, the cyclopentyl group and the amino group connected to the thiazole are exposed to the solvent and thus can be conjugated via a linker to pomalidomide (an E3 ligase ligand) (Figure 1C). We describe the discovery of a novel series of NAT10 PROTAC degraders exemplified by representative compounds NP1192 and NP1149, as shown in Figure 1D.

**Figure 1:**
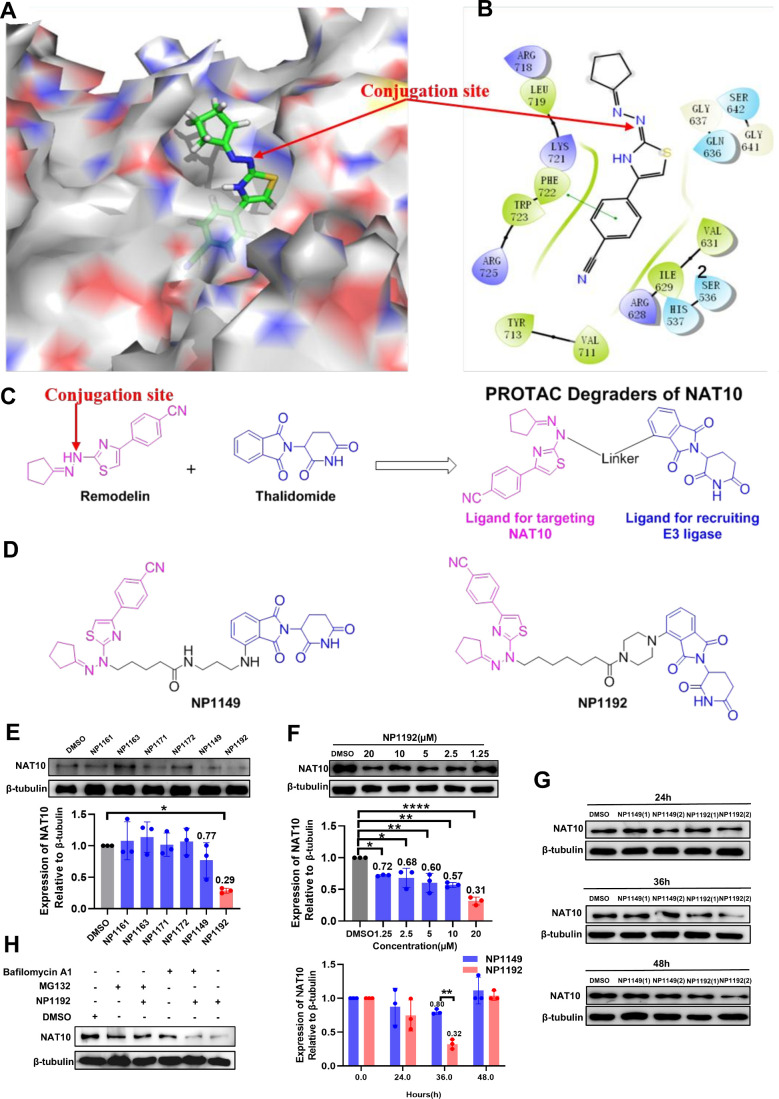
**Design strategy for a PROTAC targeting NAT10 based on Remodelin and verification of the potent degradative effect of NP1192**. (A) The binding mode of Remodelin in the NAT10 catalytic pocket. (B) Two-dimensional docking fitting diagram of Remodelin at the NAT10 binding site. (C) Design strategy for NAT10 PROTACs. (D) Representative chemical structures of the degraders NP1149 and NP1192. (E) Effects of 6 compounds (NP1161, NP1163, NP1171, NP1172, NP1149 and NP1192) on the degradation of NAT10 in SiHa cells treated at the same time and concentration. (F) Effects of NP1192 on the degradation of NAT10 in SiHa cells treated with various concentrations of the compound (0, 1.25 μM, 2.5 μM, 5 μM, 10 μM and 20 μM). (G) Effects of compounds NP1149 and NP1192 on the degradation of NAT10 in SiHa cells treated for various durations (24, 36, and 48 h). (H) Effects of NP1192 on the degradation of NAT10 in SiHa cells pretreated with NP1192 alone or in combination with MG132 or bafilomycin A1 at a concentration of 200 nM for 12 h and then treated with NP1192 for 36 h. The data represent the means ± SEM of triplicate samples. *P < 0.05, **P < 0.01, ***P < 0.001, and ****P < 0.0001.

The synthesis of **TP1–TP20** is described in Supplementary Methods. First, thiosemicarbazide **1** was reacted with cyclopentanone to obtain intermediate **2**, which was subsequently cyclized with 4-(2-bromoacetyl) benzonitrile to yield intermediate **3**. Next, substitution reactions between compound **3** and various brominated esters were conducted, which was followed by hydrolysis to generate compounds **4–7**. Second, 3-fluorophthalic anhydride **8** was converted to intermediate **9** in the presence of 3-aminopiperidine-2,6-dione hydrochloride. Next, intermediate **9** was converted with appropriate amines to intermediates **10–14**, in which the Boc group was deprotected using a 4 M HCl-dioxane solution to produce compounds **15–19**. Finally, intermediates **4–7** were coupled with intermediates **15–19** to yield compounds **TP1–TP20**.

Next, compounds **TP21–TP32** were synthesized following the synthetic route outlined in the Supplementary Methods. Briefly, compound **9** was coupled with piperidine **20**, piperazine **23** and piperazine **24** to provide intermediates **21**, **25**, and **26**, respectively. The removal of the Boc group from intermediate **21** generated compound **22**, which was condensed with intermediates **4–7** to afford compounds **TP21–TP24**. The terminal hydroxyl groups of compounds **25** and **26** were oxidized to the corresponding aldehyde intermediates **27** and **28**, which reacted with compounds **23** and **24** to produce compounds **29** and **30**, respectively. The Boc groups of compounds **29** and **30** were then deprotected to produce intermediates **31** and **32**. Finally, intermediates **31** and **32** were coupled with compounds **4–7** to yield **TP25–TP32** (Supplementary Methods).

### Identification of NP1192 as a NAT10 PROTAC Degrader That Induces Dose-Dependent and Durable NAT10 Degradation in CCa Cells

A panel of 32 newly synthesized compounds (**TP1–32**) was assessed for their ability to degrade NAT10 using Western blot (WB) analysis. Among these, **TP24** (NP1192) (71%, *P* = 0.013) and **TP12** (NP1149) (23%) exhibited the most potent degradation rates and were selected for further studies (Figure 1E). In a dose- and time-dependent manner, NP1192 treatment led to a substantial depletion of total NAT10 protein in SiHa (human) and U14 (murine) CCa cell lines. In SiHa cells, NP1192 induced degradation rates of 28% (*P* = 0.028), 32% (*P* = 0.011), 40% (*P* = 0.002), and 43% (*P* = 0.001) at concentrations of 1.25, 2.5, 5, and 10 μM, respectively, with a maximum of 69% (*P* < 0.0001) degradation at 20 μM after 36 h (Figure 1F), and only 20% degradation after 24 h (Figure 1G). Similarly, the same treatment led to a nearly 70% reduction in the NAT10 protein content in murine U14 cervical cells after 36 h (Figure 2A). Importantly, NP1192 did not exhibit the classical hook effect, a phenomenon often associated with PROTAC-mediated degradation, even at concentrations as high as 20 μM (Figure 1F–G, 2A). In contrast, NP1149 showed minimal NAT10 degradation at 36 h (20%, *P* = 0.008) with inferior to NP1192 at any tested time (Figure 1G). To explore the degradation mechanism, SiHa cells were cotreated with proteasome inhibitor MG132 or lysosomal inhibitors bafilomycin A1 except for NP1192. As anticipated, MG132 significantly impeded NP1192-mediated degradation of NAT10, whereas bafilomycin A1 exerted no discernible effect on NP1192’s efficacy (Figure 1H), thereby confirming that NP1192 facilitates NAT10 degradation through the ubiquitin-proteasome system (UPS) rather than via lysosomal pathways.

**Figure 2:**
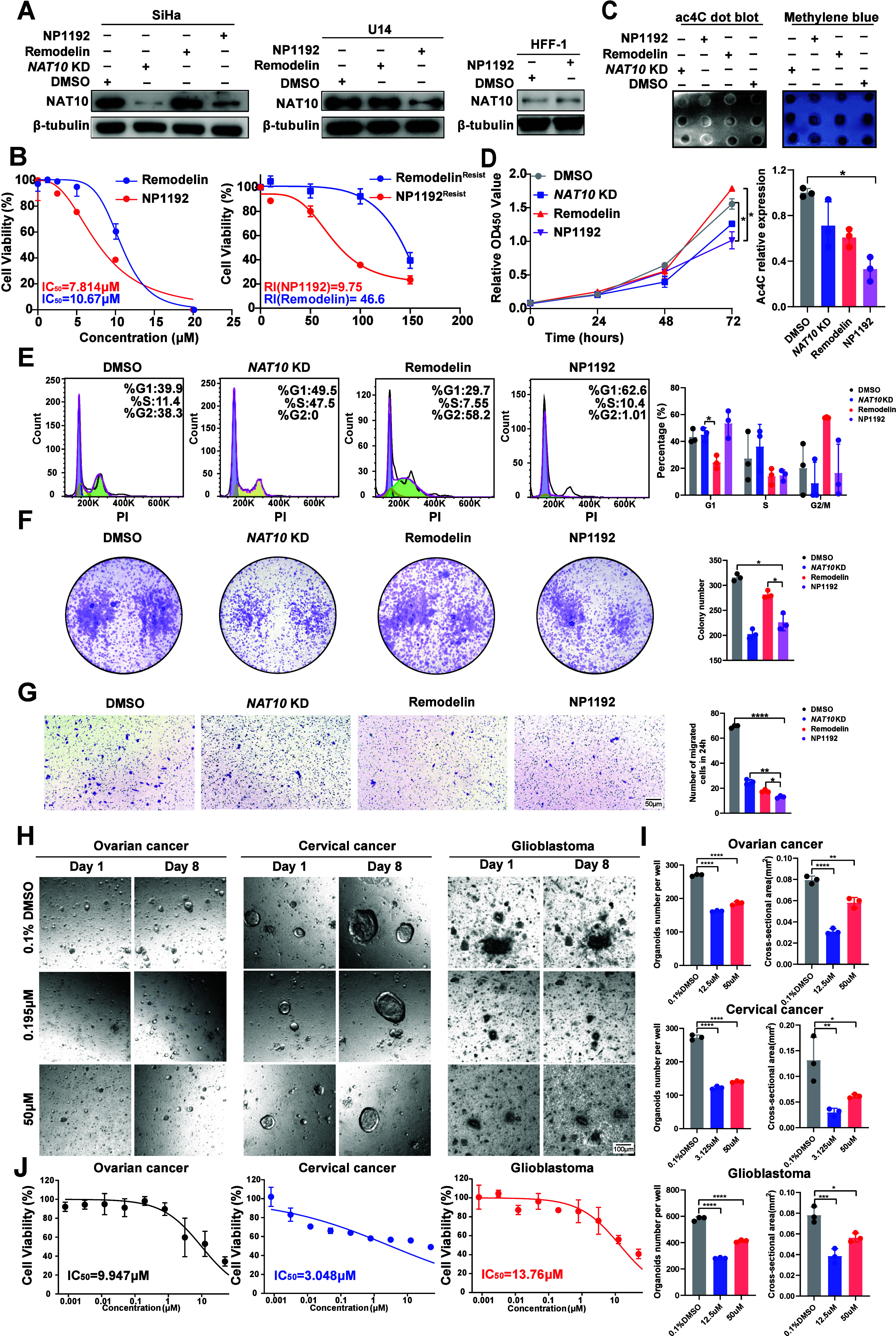
**The NAT10 PROTAC NP1192 is a highly effective NAT10 PROTAC that promotes** ***NAT10*** **KD and inhibits ac4C modification**. (A) Effects of DMSO, Remodelin, and NP1192 treatment or NAT10 knockout on NAT10 degradation in SiHa, U14, and HFF-1 cells. The cells were treated with Remodelin or NP1192 at a concentration of 20 μM for 36 h. (B) The IC_50_ values of Remodelin and NP1192 for the inhibition of SiHa cell viability after treatment for 36 h. Dose–response curves of NP1192 and Remodelin resistant SiHa cells with resistance index (RI = IC_50_ resistant/parental SiHa). (C) Global ac4C abundance in SiHa cells and NAT10-KD SiHa cells after treatment with DMSO, Remodelin, or NP1192 for 36 h. The cells were treated with Remodelin or NP1192 at a concentration of 20 μM. (D) Inhibition of the proliferation of SiHa cells and NAT10-KD SiHa cells after treatment with DMSO, Remodelin or NP1192, as measured by CCK-8 assays at 0, 24, 48, and 72 h. The cells were treated with Remodelin or NP1192 at a concentration of 20 μM. (E) Cell cycle arrest in SiHa cells and NAT10-KD SiHa cells after 36 h of treatment with DMSO, Remodelin, or NP1192, as validated by flow cytometry. The cells were treated with Remodelin or NP1192 at a concentration of 20 μM. (F) Effects of DMSO, Remodelin, and NP1192 treatment or NAT10 knockout on the proliferative capacity of SiHa cells, as measured by colony formation assays after 2 weeks. The cells were pretreated with Remodelin or NP1192 at a concentration of 20 μM after 36 h. (G) Effects of DMSO, Remodelin, or NP1192 treatment on the invasion of SiHa cells and NAT10-KD SiHa cells after 12 h, as measured via Transwell invasion assays. The cells were pretreated with Remodelin or NP1192 at a concentration of 20 μM after 36 h. (H–J) Representative images of three distinct PDOs (OVs, CESCs, and GBMs) after treatment with NP1192 at concentrations of 0.195 μM, 50 μM or 0.1% DMSO on the first and eighth days (H). The organoid number and cross-sectional area per well were calculated (I). The IC_50_ values of NP1192 for the inhibition of cell viability in the three types of PDOs were also determined (J). The data represent the means ± SEM of triplicate samples. *P < 0.05, **P < 0.01, ***P < 0.001, and ****P < 0.0001.

### Superior Efficacy of NP1192 in Targeting NAT10 and Inhibiting RNA Acetylation than Remodelin in SiHa Cells

Previous investigations have established that Remodelin, a potent NAT10 inhibitor, exerts its effects by suppressing the lysine acetyltransferase (KAT) activity of NAT10.^10^ However, NP1192 exhibited significantly greater efficacy in reducing CCa cell viability, as reflected by its substantially 26.8% lower IC_50_ value in SiHa cells (IC_50_ = 7.814 μM for NP1192 vs 10.67 μM for Remodelin). Specifically, we generated Remodelin-resistant (Remodelin^Resist^ SiHa) and NP1192-resistant (NP1192^Resist^ SiHa) CCa cell lines by applying a stepwise dose escalation strategy, as previously described in the literature.^21^ The IC_50_ for NP1192^Resist^ SiHa cells was determined to be 76.2 μM, whereas the IC_50_ for parental SiHa cells was 7.814 μM as previously detected, yielding an RI of 9.75 (76.2/7.814). Similarly, the Remodelin^Resist^ SiHa cells also demonstrated an RI of 46.60 (497.3/10.67). Both resistant cell lines demonstrated RI over 5, confirming the successful establishment of drug-resistant sublines.^22^ Moreover, the higher RI suggesting that Remodelin may be prone to significant resistance development during therapeutic administration (Figure 2B).^22,23^ Additionally, NP1192 elicited a more pronounced depletion in NAT10 protein levels compared to Remodelin, with its effect approaching those observed with the CRISPR-Cas9-mediated NAT10 knockout. Notably, NP1192 treatment substantially induced minimal cytotoxicity in nonmalignant HFF-1 cells with low NAT10 expression compared to the neoplastic, underscoring its potential as a selective anticancer therapeutic (Figure 2A).

Owing to the acetyltransferase function of NAT10 and the known effect of Remodelin, it was hypothesized that NP1192 would attenuate cellular acetylation levels. Consistent with this, RNA dot blot analysis specifically assessing RNA ac4C modifications revealed a significant reduction in ac4C levels in NP1192-treated cells compared with both Remodelin-treated and NAT10-knockdown groups (Figure 2C). These results further validate the superior efficacy of NP1192 in diminishing NAT10-mediated RNA acetylation. Collectively, these findings position NP1192 as a potent, selective NAT10-targeting PROTAC degrader capable of robustly depleting NAT10 protein and inhibiting ac4C modification in cancer cells.

### NP1192 reduces CCa cell viability and has potent anticancer efficacy *in vitro* and *in vivo*

*In vitro* assessments established NP1192 as a potent NAT10 protein degrader in CCa cells. Cell phenotypic experiments were further carried out to explore the effect of NP1192 on the proliferation and invasion of CCa cells. CCK-8 assays showed that NP1192 reduced cell viability with increased cell cycle arrest detected in the cell cycle determination (Figure 2D–E). Furthermore, NP1192 treatment led to a substantial decrease in colony formation capacity, with the magnitude of inhibition being only second to that in NAT10-knockdown cells, whereas Remodelin treatment exerted minimal suppressive effects (Figure 2F). Additionally, Transwell invasion assays revealed that NP1192 substantially hindered CCa cell invasion, with a significant decrease in the number of cells crossing the basement membrane, mirroring results from NAT10 knockdown experiments (Figure 2G). These findings collectively highlight NP1192’s potent tumor-suppressive properties, inhibiting both cell proliferation and malignant progression. Having identified the favorable *in vitro* antiproliferative activity, we further evaluated the pharmacokinetic properties of compound NP1192 in male Sprague–Dawley (SD) rats. As shown in Figure S1 and Table S1, after intravenous (2 mg/kg) or oral (10 mg/kg) administration of NP1192, the AUC_(0–24h)_ values of NP1192 in plasma were 365 ± 42.6 and 24.7 ± 1.11 (ng/mL·h), respectively, indicating that NP1192 has a low oral exposure. The *C*_max_ of NP1192 in plasma were 759 ± 134 and 13.9 ± 6.42 (ng/mL) when administered intravenously and orally, respectively, and oral bioavailability (F) was 1.35%, probably due to the high molecular weight (MW = 734.30) and lipophilicity (LogP = 5.29) of NP1192. In addition, NP1192 exhibited appropriate half-lives (0.967 ± 0.214 and 2.40 ± 1.15 h for iv and po administration, respectively).

To corroborate the results *in vivo*, subcutaneous xenograft models were established using U14 cells in C57BL/6J mice, followed by treatment with NP1192 or Remodelin (Figure 3H). NP1192 significantly reduced tumor volume compared to DMSO or Remodelin at an equimolar dose of 25 mg/kg (Figure 3F). *In vivo* bioluminescence imaging performed at 48-h intervals demonstrated a more substantial decline in tumor burden following NP1192 treatment than Remodelin, underscoring its superior pharmacological degraded potency (Figure 3L). Notably, NP1192 treatment did not cause significant weight loss or systemic toxicity, suggesting a favorable safety profile for clinical use (Figure 3F–G). Finally, we evaluated the acute toxicity of NP1192 in C57BL/6J mice, as shown in Figure S2, and a dose of 1000 mg/kg was well-tolerated by the C57BL/6J mice, indicating the benign safety profiles of NP1192 (LD_50_ > 1000 mg/kg).

**Figure 3:**
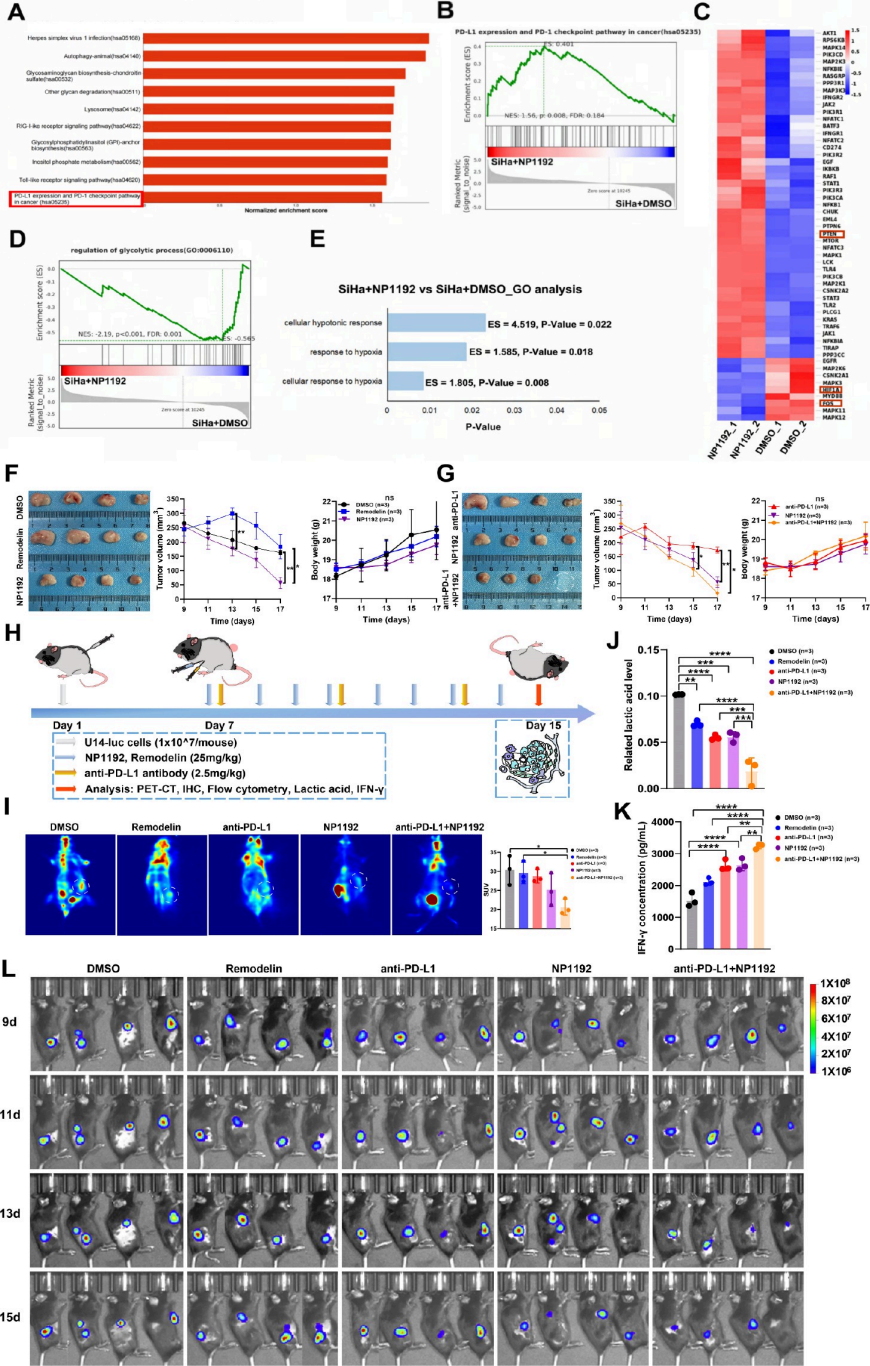
**Treatment with NAT10 PROTAC NP1192 modulates the signaling pathways affected by PD-1/PD-L1 immune checkpoint blockade**. (A–C) GSEA enrichment pathway bar plot showing the top 10 pathways enriched with upregulated genes upon NP1192 treatment at a concentration of 20 μM after 36 h (A). GSEA enrichment pathway map showing that the PD-1/PD-L1 immune checkpoint blockade pathway is positively associated with NP1192 treatment at a concentration of 20 μM after 36 h (B). Heatmap showing the expression of the genes in the PD-1/PD-L1 immune checkpoint blockade pathway (C). (D) GSEA enrichment pathway map showing that regulation of the glycolytic process pathway is positively associated with NP1192 treatment at a concentration of 20 μM after 36 h. (E) GO enrichment pathway bar plot showing the enrichment of three hypoxia-related pathway alterations upon NP1192 treatment at a concentration of 20 μM after 36 h. (F–H) Experimental flowchart (H) and effects of DMSO, Remodelin, anti-PD-L1 blockade, NP1192 or anti-PD-L1 blockade plus NP1192 on tumor volume and body weight (F and G) in C57BL/6J mouse xenograft models after treatment for 1 week. (I) PET–CT scan showing the biodistribution of ^18^F-FDG at the tumor location in xenograft model mice treated with various therapies for 1 week. The SUV values of the different groups were also analyzed. (J) Effects of various treatments on the lactic acid content of tumors in xenograft mouse models after treatment for 1 week. (K) ELISA was used to measure the IFN-γ concentration in plasma collected from xenograft model mice treated with various therapies for 1 week. (L) *In vivo* bioluminescence imaging of xenograft model mice treated with DMSO, Remodelin, anti-PD-L1 blockade, NP1192 or anti-PD-L1 blockade plus NP1192 on the 9th, 11th, 13th, and 15th days. The data represent the means ± SEM of triplicate samples. *P < 0.05, **P < 0.01, ***P < 0.001, and ****P < 0.0001.

To more accurately mimic the complexity of tumor heterogeneity and intercellular interactions, patient-derived organoids (PDOs) were established from epithelial stem cells of high-grade serous ovarian carcinoma (OV), cervical squamous cell carcinoma and endocervical adenocarcinoma (CESC), and glioblastoma (GBM).^24–32^ By comparing the photography pictures on the first and eighth days, NP1192 treatment induced a pronounced reduction in organoid size and number across all three tumor models, as compared to the 0.1% DMSO-treated controls (Figure 2H–I). Importantly, the architecture and cellular integrity of the corresponding normal organoids remained unaltered following NP1192 exposure, indicating minimal cytotoxicity to nonmalignant tissues and corroborating findings from previous *in vitro* and *in vivo* evaluations (Figure 2H). To further assess the therapeutic sensitivity, ATP-based viability assays were performed across the organoid models. NP1192 exhibited a clear dose-dependent inhibitory effect, with IC_50_ at 9.947 μM for OV, 3.048 μM for CESC, and 13.76 μM for GBM (Figure 2J). Notably, the IC_50_ values derived from organoid models were consistently lower than that observed in CCa cell lines (IC_50_ = 7.814 μM) (Figure 2B), suggesting improved pharmacodynamic efficacy in more physiologically relevant models.

In conclusion, the results underscore NP1192’s robust inhibitory effects on cancer proliferation and invasion with low toxicity profile both *in vitro* and *in vivo*, thereby holding great promise for clinical application at relatively feasible therapeutic concentrations.

### NP1192 Primarily Results in the Modulation of PD-1/PD-L1 Immune Checkpoint Signaling Pathways

To elucidate the molecular mechanisms underpinning the antitumor effects of PROTAC NP1192, RNA sequencing was conducted to assess differential gene expression patterns. Gene set enrichment analysis (GSEA) identified a broad spectrum of dysregulated pathways following NP1192 treatment (Figure 3A). Among these, significant enrichment of the PD-1/PD-L1 immune checkpoint pathway drew particular attention (NES = 1.56, *P* = 0.008) (Figure 3B). The PD-1/PD-L1 immune checkpoint pathway plays a pivotal role in immune evasion and therapy of CCa.^33,34^ Human papillomavirus (HPV) infection promotes PD-L1 expression through regulating multiple signaling pathways (e.g., YAP, HIF-1α, Wnt/β-catenin, JAK-STAT, PI3K/AKT, and STING-TBK1), enabling tumor cells to evade immune system attacks.^35–37^ PD-L1 expression level is closely associated with the prognosis of CCa and possesses predictive value in immunotherapy.^38^ However, the relationship between NAT10 and the PD-1/PD-L1 pathway requires further investigation to clarify its potential role in CCa immunotherapy. Although targeting the PD-1/PD-L1 axis has proven effective in enhancing antitumor immunity, the response rate remains low in most patients.^39–42^ Transcriptomic profiling of NP1192-treated cells revealed downregulation of oncogene FOS and glycolysis-related gene *HIF1A*, while tumor suppressor PTEN was upregulated (Figure 3C). Intriguingly, NP1192 administration also affected regulation of glycolytic process pathway (NES = −2.19, *P* < 0.001) with significant alterations in genes involved in glycolysis, indicating a profound impact on tumor cell metabolism (Figure 3C–D). Gene ontology (GO) enrichment analysis of SiHa cells treated with NP1192 further demonstrated significant changes in hypoxia-related pathways, including “cellular response to hypoxia” (ES = 1.805, *P* = 0.008), “response to hypoxia” (ES = 1.585, *P* = 0.018), and “cellular hypotonic response” (ES = 4.519, *P* = 0.022) (Figure 3E). These findings suggest that NP1192 not only modulates NAT10 activity but also alters the hypoxic TME. Additionally, transcriptomic analysis revealed enrichment of cell cycle-related pathways, particularly in cell division and G1/S transition, consistent with previous observations of cell cycle arrest (Figure S3A). The data also revealed enrichment of immune system processes and cell killing-related biological processes (Figure S3B).

Prior studies have established that hypoxia-induced upregulation of PD-L1 enhances tumor immune evasion and fosters an immunosuppressive TME, ultimately conferring resistance to PD-L1 blockade therapy.^43–46^ In this context, NP1192 was shown to reverse the hypoxic microenvironment in CCa, thereby achieving tumor suppression *in vitro*. Thus, we postulated that the combination of NP1192 and anti-PD-L1 antibody may exhibit synergistic anticancer effects and optimize the efficacy of immunotherapy. Hypodermatic xenograft models were established in immunocompetent mice, and various treatment regimens were evaluated *in vivo*, with agents including DMSO, Remodelin, anti-PD-L1 antibody, NP1192, and the NP1192-anti-PD-L1 combination administered intraperitoneally every 2 or 3 days. Tumor growth and *in vivo* imaging were monitored every 2 days (Figure 3H). The combination significantly inhibited CCa xenograft tumor growth, outperforming both NP1192 and the anti-PD-L1 antibody alone (Figure 3G).

Similarly, *in vivo* imaging corroborated these findings (Figure 3L). The combined treatment also yielded the most pronounced reduction (>80%, *P* < 0.001) in tumor lactate levels, indicating improved metabolic control followed by more than 40% decline in NP1192 group (Figure 3J). Additionally, ^18^F-FDG-PET-CT scans revealed minimal metabolic uptake in the combination-treated group, further suggesting that NP1192 effectively suppresses tumor metabolic activity (Figure 3I).

### NP1192 Pharmacologically Inhibits HIF-1α-Induced Hypoxia and Immune Evasion via the NAT10/ac4C-HIF-1α Axis

Given the established role of PD-L1 in promoting tumor immune evasion, we hypothesized that regulation of the PD-1/PD-L1 signaling axis involves multiple transcription factors modulated by acetylation and suppressed by NP1192. NAT10, known for enhancing mRNA stability and translation through acetylation within the coding sequence (CDS), was studied previously by us using RNA-seq, acRIP-seq and ribo-seq on NAT10 knockout SiHa and untreated control cells.^9^ acRIP-seq analysis revealed 808 ac4C peaks in SiHa cells, revealing high enrichment of the CXXCXXCXX motif, predominantly near protein-coding regions, with a notable shift in ac4C distribution from coding regions to the 3′ UTR in cells with low NAT10 expression. The validity and correctness of ribo-seq have also been proven.^9^ Furthermore, we conducted an in-depth analysis of all genes clustered within the PD-1/PD-L1 pathway and cross-referenced them across four sequencing data sets (acRIP-seq, ribo-seq, RNA-seq with *NAT10* KD or NP1192 treatment) to generate a Venn diagram (Figure 4A–B). One hypoxia-related gene that consistently exhibited decreased expression across all four sequencing data sets was HIF-1α, a known pro-tumorigenic factor, regulating PD-L1 under hypoxic conditions.^47^ The decreased peak value in the IGV plot of the acRIP-seq data after NAT10 knockdown indicated the impaired epigenetic ac4C modification of *HIF1A*, and acRIP-qPCR further confirmed decreased acetylation with NP1192 treated or NAT10 knockdown (Figure 4C, P). Moreover, both the GO and KEGG analyses revealed alterations in the hypoxia signaling pathways, including “HIF-1 signaling pathway” (ES = 1.426, *P* = 0.003), “response to hypoxia” (ES = 1.233, *P* = 0.020), and “cellular response to hypoxia” (ES = 1.489, *P* = 0.0004) (Figure 4D). The transcriptome data revealed a significant decrease in *HIF1A* mRNA expression following NAT10 knockdown (Figure 4G). Additionally, NAT10 knockdown or applying NP1192 led to decreased *HIF1A* mRNA stability (Figure 4E) and a reduction in global nascent protein translation (Figure 4O). NP1192 treatment also decreased HIF-1α protein levels, as validated by WB (Figure 4F), supporting the idea that NP1192 suppresses HIF-1α transcription and translation by inhibiting NAT10-mediated ac4C modification, disrupting the hypoxic response.

**Figure 4:**
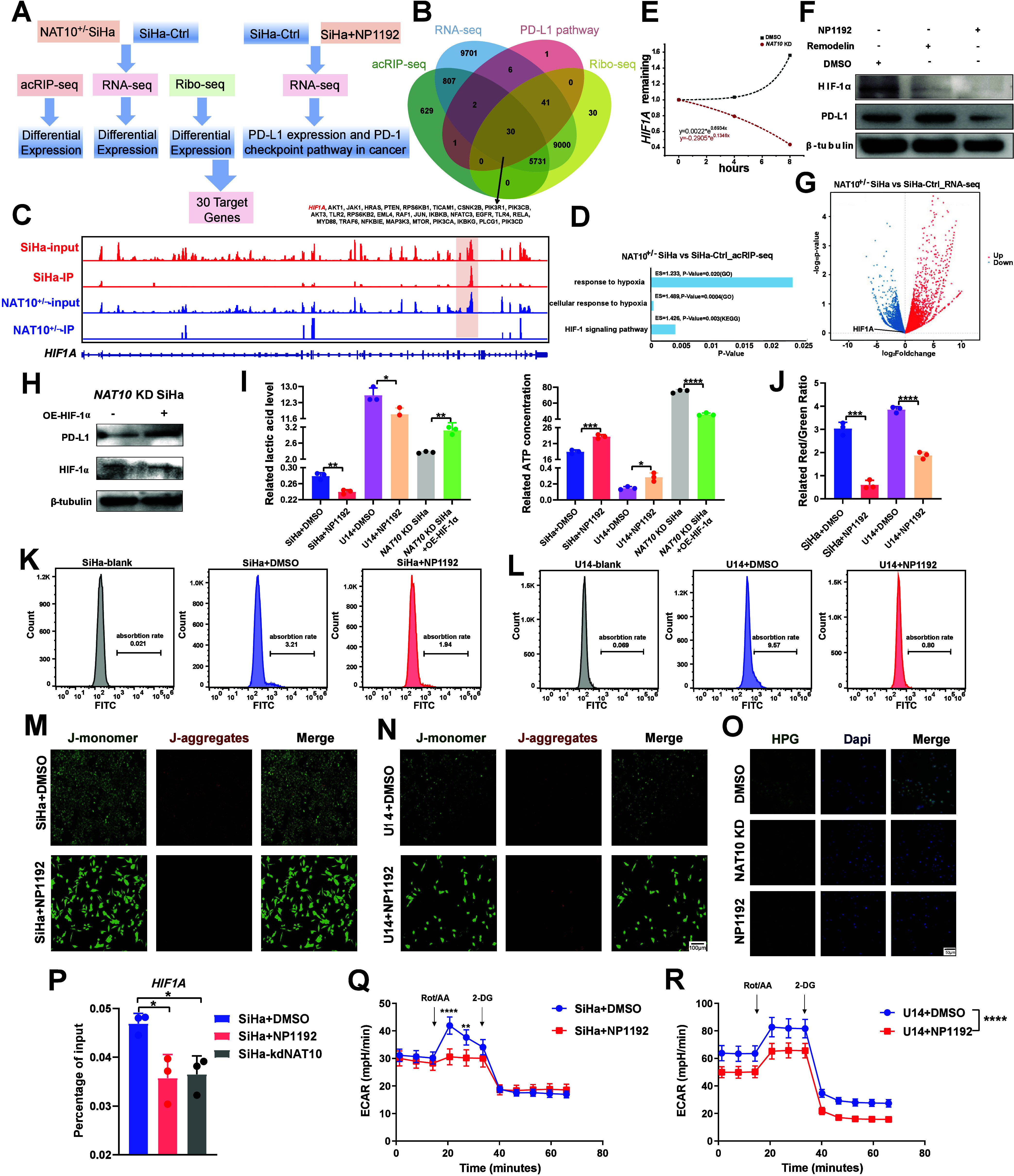
**The NAT10/ac4C-** ***HIF1A*** **axis regulates hypoxic tumor immune evasion by pharmacologically inhibiting HIF-1α-induced hypoxia**. (A–B) Flowchart (A) and Venn diagram (B) showing overlapping genes among RNA-seq, acRIP-seq and Ribo-seq data from NAT10^±^ SiHa cells and the PD-1/PD-L1 immune checkpoint blockade pathway of NP1192-treated SiHa cells. (C) IGV plot showing ac4C acetylation profiles for *HIF1A* mRNA according to acRIP-seq between control and NAT10^±^ SiHa cells. (D) KEGG and GO enrichment pathway bar plots showing three hypoxia-related pathways enriched in the acRIP-seq data compared with those enriched in the NAT10^±^ SiHa cells. (E) Fitted curve of the mRNA stability assay results showing that *HIF1A* mRNA remaining in the control and NAT10^±^ SiHa cells after treatment with actinomycin D for 0, 4, or 8 h. (F) Western blot analysis showing the downregulation of PD-L1 and HIF-1α in SiHa cells upon treatment with Remodelin or NP1192 for 36 h. (G) Volcano plot showing the decreased expression of *HIF1A* mRNA in NAT10^±^ SiHa cells. (H) Western blot analysis showing the upregulation of PD-L1 and HIF-1α in NAT10-knockdown SiHa cells upon OE-HIF-1α. (I, K–L) Effects of different pretreatments for 36 h on lactic acid production and intracellular ATP levels (I), and glucose absorption ratios (K–L) in SiHa and U14 cells. (J, M–N) Representative images from the mitochondrial membrane potential assay with JC-1 in DMSO- and NP1192-treated SiHa or U14 cells after 36 h. J-monomers (green) and J-aggregates (red) are shown (M–N). The related red/green ratios are also shown (J). (O) Representative images from the nascent protein synthesis detection assay in DMSO-, NP1192- or *NAT10*-KD-treated SiHa cells after 36 h. DAPI (blue) and HPG (green) are shown. (P) acRIP-qPCR results showing decreases in the IP/input percentage of SiHa groups treated with NP1192 at a concentration of 20 μM for 36 h and NAT10-knockdown compared with DMSO treatment. (Q–R) Glycolysis rate tests showing the glycolytic capacity of SiHa (Q) and U14 (R) cells after pretreatment with DMSO or NP1192 at a concentration of 20 μM. The data represent the means ± SEM of triplicate samples. *P < 0.05, **P < 0.01, ***P < 0.001, and ****P < 0.0001.

In accordance with current oncological paradigms, intratumoral hypoxia is recognized as a fundamental hallmark of the TME, primarily orchestrated by HIF-1α.^48–51^ Treatment with NP1192 significantly decreased lactate levels—an end-product of glycolysis—in SiHa and U14 cells (Figure 4I), while also reducing glucose uptake upon exposure to a glucose analog, indicating suppressed glycolytic flux (Figure 4K–L). Conversely, the level of ATP production increased markedly, suggesting a shift toward oxidative phosphorylation (Figure 4I). JC-1 fluorescence staining revealed reduced red:green ratios, further supporting enhanced mitochondrial activity (Figure 4J, M–N). *In vivo* ^18^F-FDG-PET-CT metabolic imaging demonstrated a significant decline in glucose uptake in NP1192-treated xenografts compared with anti-PD-L1 antibody-treated controls (Figure 3I). Seahorse extracellular flux analysis showed that although ECAR rose after Rot/AA addition, the glycolytic capacity of NP1192-treated CCa cells remained markedly impaired compared to controls (Figure 4Q–R). Further, we reintroduced HIF-1α into the NAT10-depleted model (*NAT10* KD SiHa) to robustly validate that NAT10 influences tumor immunity through HIF-1α. To this end, we first constructed a plasmid encoding human *HIF1A* for overexpression and successfully transfected it into *NAT10* KD SiHa cells, with efficient overexpression confirmed by Western blot analysis on protein extracts from both groups (Figure 4H). Subsequently, we lysed both the control *NAT10* KD SiHa cells and those overexpressing *HIF1A*, and we assessed their lactate production and ATP levels. The results demonstrated that reintroduction of *HIF1A* significantly increased lactate accumulation while reducing intracellular ATP levels, thereby confirming that NAT10 positively regulates glycolysis via HIF-1α (Figure 4I). These findings underscore NP1192’s efficacy in abrogating HIF-1α-driven glycolysis and hypoxic adaptation.

Moreover, suppression of HIF-1α by NP1192 also resulted in pronounced downregulation of PD-L1, a critical immune checkpoint molecule facilitating tumor immune escape (Figure 4F). The data also revealed that HIF-1α reconstitution in *NAT10* KD SiHa cells led to an upregulation of PD-L1 protein levels (Figure 4H). As a key immune checkpoint molecule, PD-L1 contributes to tumor immune evasion by binding to the PD-1 receptor on T cells, thereby inhibiting T cell activation, proliferation, and cytotoxic function.^52–54^ The above results indicate that NAT10 promotes tumor immune evasion via HIF-1α-mediated PD-L1 up-regulation. This dual inhibition of HIF-1α and PD-L1 suggests that NP1192 effectively disrupts the hypoxia-immunosuppression axis, highlighting its potential to enhance antitumor immunity and inhibit immune evasion within the TME.

### The NAT10 PROTAC NP1192 Attenuates Hypoxia-Induced Glycolysis by Reversing the Effect of 0.1% O_2_-Induced Hypoxia

To further elucidate the inhibitory effects of the NAT10 PROTAC NP1192 on glycolysis induced via HIF-1α activation through NAT10-mediated ac4C modification, we intensified hypoxic conditions by establishing a severely oxygen-deprived microenvironment (0.1% O_2_) in tumor cells and conducted rescue experiments to evaluate alterations in key glycolysis-related parameters. Functionally, NP1192 reversed hypoxia by downregulating HIF-1α expression and suppressing glycolysis. The induction of hypoxia in CCa cells by exposure to a 0.1% aqueous O_2_ environment was confirmed by the accumulation of reactive oxygen species (ROS). Compared with normal conditions, exposure to 0.1% O_2_ resulted in a sharp increase in the ROS content, but treatment with NP1192 reduced ROS generation, indicating partial alleviation of hypoxia (Figure 5H).

**Figure 5:**
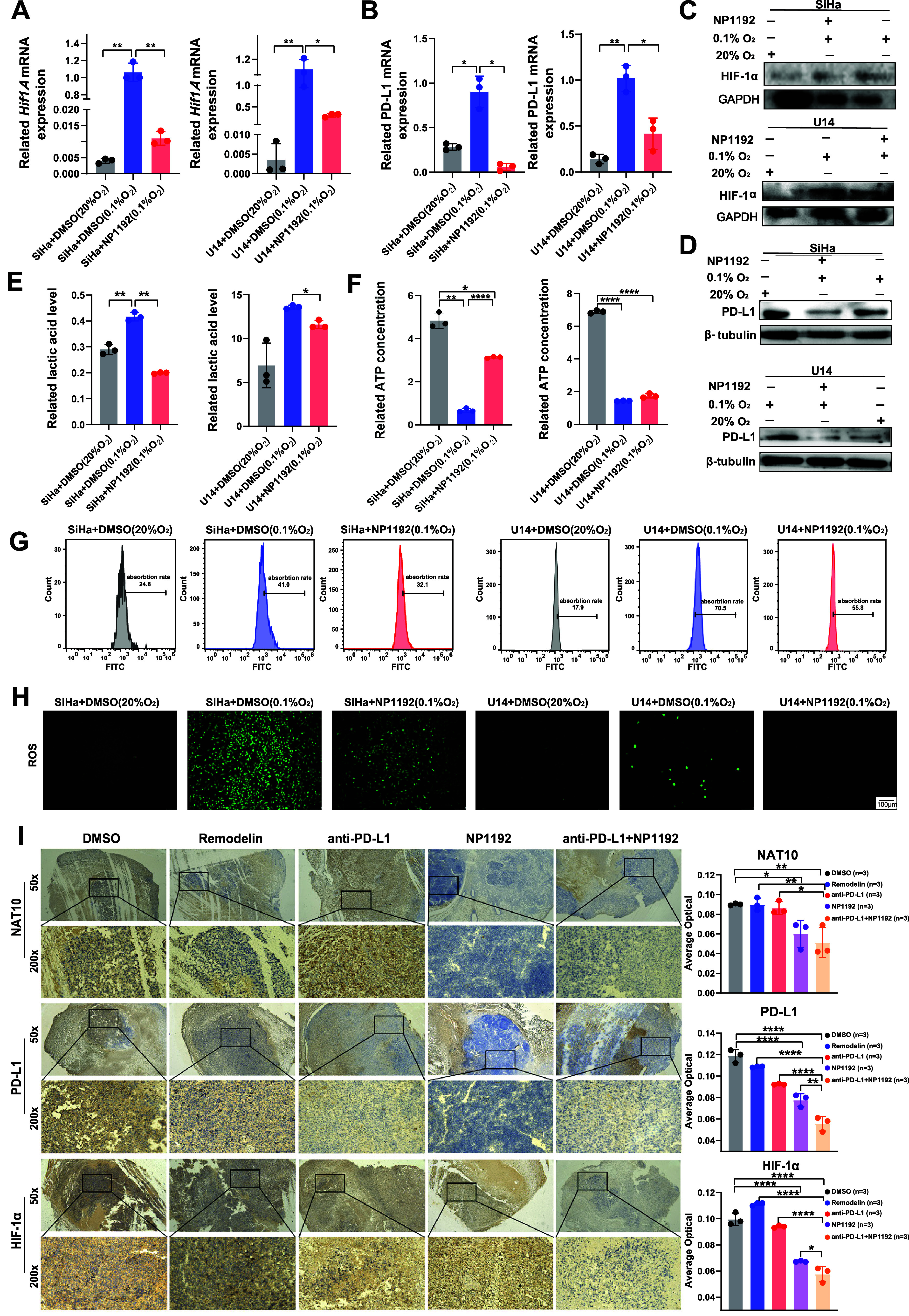
**The NAT10 PROTAC NP1192 dampens hypoxia-induced glycolysis by reversing the effect of 0.1% O**_**2**_ **-induced hypoxia**. (A–B) qPCR analysis of PD-L1 and *HIF1A* revealed mRNA levels after normalization to the levels of the reference gene GAPDH in DMSO- or NP1192-treated SiHa or U14 cells cultured in 0.1% O_2_ or a normal atmosphere; NP1192 was applied at a concentration of 20 μM for 36 h. (C–D) Western blot analysis showing PD-L1 and HIF-1α protein levels in DMSO- or NP1192-treated SiHa cells cultured in 0.1% O_2_ or a normal atmosphere; NP1192 was applied at a concentration of 20 μM for 36 h. (E–G) Effects of DMSO or NP1192 treatment on lactic acid production (E), intracellular ATP levels (F), and glucose absorption ratios (G) in DMSO- or NP1192-treated SiHa and U14 cells cultured in 0.1% O_2_ or a normal atmosphere; NP1192 was applied at a concentration of 20 μM for 36 h. (H) Representative images from the ROS staining assay in DMSO- or NP1192-treated SiHa or U14 cells cultured in 0.1% O_2_ or a normal atmosphere; NP1192 was applied at a concentration of 20 μM for 36 h. ROS (green) are shown. (I) Representative images from IHC experiments showing the expression of NAT10, PD-L1 and HIF-1α in tumor tissues from xenograft model mice subjected to various treatments after treatment for 1 week. The relative average optical densities of the proteins in the different groups were also analyzed. The data represent the means ± SEM of triplicate samples. *P < 0.05, **P < 0.01, ***P < 0.001, and ****P < 0.0001.

Under hypoxic conditions, HIF-1α expression can be amplified, triggering a cascade of signaling events that activate the PD-1/PD-L1 immune checkpoint pathways, ultimately leading to diminished immunogenicity, enhanced tumor cell immune evasion and compromised efficacy of cancer immunotherapy.^55^ Consistent with this observation, hypoxic conditions extensively induced the mRNA and protein expression of hypoxia-related genes, such as *HIF1A* and PD-L1, which could be effectively alleviated by the NP1192 treatment (Figure 5A–D). Similarly, NP1192 markedly suppressed the production of the glycolytic product lactate, interfered with glucose uptake, and enhanced ATP generation under hypoxia (Figure 5E–G). Additionally, an immunohistochemical analysis of two hypoxia-related genes, HIF-1α and PD-L1, revealed significant reductions in their expression levels following combined treatment with NP1192 and anti-PD-L1 blockade, which dampened NAT10 activity *in vivo* (Figure 5I).

### NAT10 Deficiency Induced by Treatment with NP1192 Promotes CD8^+^ T_eff_ Infiltration and Effector Function

To elucidate the mechanisms of NP1192-induced immune modulation, we performed single-cell RNA sequencing (scRNA-seq) on tumor samples from immunocompetent mice treated with DMSO or NP1192. Unsupervised graph-based clustering and visualization through T-distributed stochastic neighbor embedding (t-SNE) revealed six distinct cell clusters, annotated based on canonical markers and gene expression profiles (Figure 6A–B). NP1192 treatment significantly reduced the proportion of immunosuppressive M2 macrophages and myeloid-derived suppressor cells (MDSCs), while increasing the M1 macrophages, suggesting a reversal of the immunosuppressive TME (Figure 6C). These findings were also supported by gene expression changes specific to each cell population (Figure 6G).

**Figure 6:**
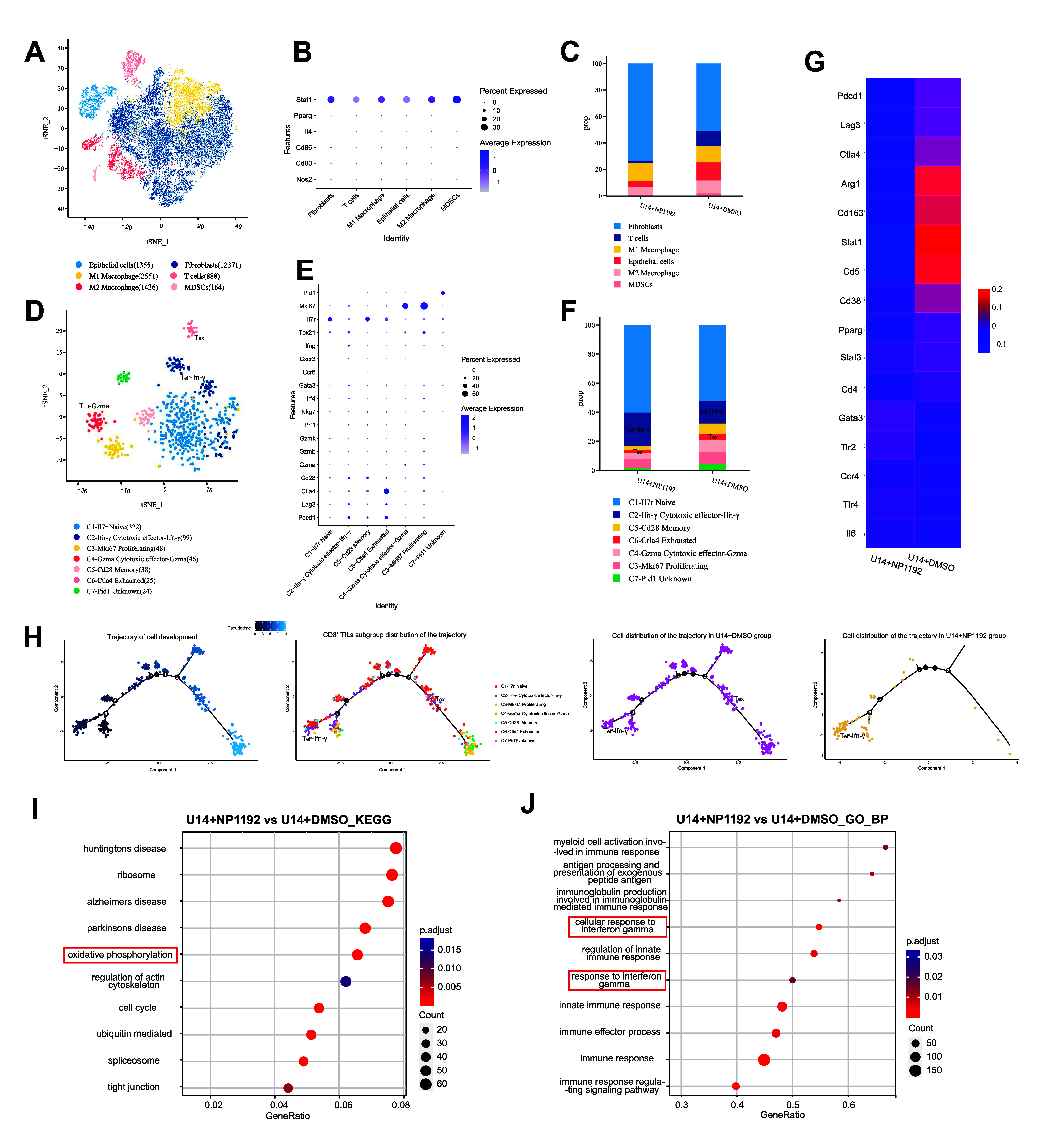
**NAT10 deficiency induced by NP1192 promotes CD8** ^**+**^ **T**_**eff**_ **infiltration and responses**. (A–B) t-SNE plot of TME cells in control and NP1192-treated tumors after 1 week (A). Dot plots of gene expression in each subcluster (B). (C) Bar graph showing the percentage of each subcluster of TME cells. (D–E) t-SNE plot of CD8^+^ TILs subpopulations in control and NP1192-treated tumors after 1 week (D). Dot plots of gene expression in each subcluster (E). (F) Bar plot showing the percentage of each subcluster of CD8^+^ TILs. (G) Expression of marker genes of different immune populations according to the scRNA-seq analysis of control and NP1192-treated tumors. (H) The pseudotime differentiation map shows the trajectory of cell development, individual cell types, and the variation in cell density in each sample and subgroup with trajectory development. (I–J) Maps of the enriched GO and KEGG pathways in single-cell sequencing data showing that oxidative phosphorylation increased with the addition of NP1192 (I). GO enrichment analysis revealed that NP1192 upregulated the response to interferon-gamma-related pathways (J). The data represent the means ± SEM of triplicate samples. *P < 0.05, **P < 0.01, ***P < 0.001, and ****P < 0.0001.

NAT10 has previously been linked to the inhibition of CD8^+^ tumor-infiltrating lymphocyte (TIL) recruitment and function in prostate cancer via the CCL25/CCR9 axis.^56^ To explore the effects of NP1192 on CD8^+^ TILs of CCa, we reclustered these cells and identified seven subpopulations based on gene expression: naive, cytotoxic effector-Ifn-γ, memory, exhausted, cytotoxic effector-Gzma, proliferating, and unknown (Figure 6D). Dot plot of signature genes for each subset further clarified these populations (Figure 6E). Notably, NP1192-treated tumors exhibited fewer exhausted CD8^+^ TILs (T_ex_) and more cytotoxic effector-like CD8^+^ TILs (T_eff_), indicating enhanced immune activation. Ifn-γ-producing EF-like CD8^+^ TILs infiltrate the tumor-associated immune milieu and constitute a considerable population of CD8^+^ TILs, demonstrating the ability of NP1192 to mediate immune-mediated killing programs (Figure 6F). Subsequently, we examined whether targeting NAT10 could influence the developmental trajectory of these CD8^+^ TIL subtypes. Pseudotime analysis revealed that the naive, cytotoxic effector-Ifn-γ and proliferating subtypes accounted for the main proportions in the initial state, whereas the T_ex_ subtypes were maintained in the advanced stage. Concomitantly, the majority of the NP1192-treated cells were sustained at the initial stage, with heightened expression levels of Ifn-γ and Tnf-α in accordance with the cell collections mentioned above. Notably, the proliferating CD8^+^ TIL population displaying the Mki67 signature emerged earlier in the NP1192-treated group than in the DMSO-treated group, which indicated the robust CD8^+^ TIL propagation capacity elicited by NP1192 (Figures 6H and S3C). Additionally, NP1192 upregulated oxidative phosphorylation and numerous immune effector pathways, especially CD8^+^ T_eff_-related “cellular response to interferon gamma” and “response to interferon gamma”, reinforcing its role in enhancing CD8^+^ TIL function and promoting an antitumor immune response (Figure 6I–J).

PD-L1 is known to inhibit dendritic cell maturation, promote regulatory T cells (Tregs), and reduce the cytotoxic activity of natural killer (NK) cells and neutrophils, thus aiding immune evasion. As a result, therapeutic strategies incorporating anti-PD-L1 blockade have been increasingly employed to overcome the formidable challenge of therapeutic resistance in oncological interventions.^57–59^ To evaluate the effects of NP1192 combined with anti-PD-L1 antibody and NP1192 or anti-PD-L1 blockade alone on CD8^+^ TILs, immunoflow cytometry was performed after tumor excision. The combination treatment showed the highest frequency of CD8^+^ TILs, followed by NP1192 monotherapy, indicating substantial therapeutic benefits. CD8^+^ TILs predominantly secrete IFN-γ during the antigen-specific immune response, suggesting the activation of effector TILs. ELISA results confirmed increased IFN-γ production in combination group (Figure 3K), correlating with enhanced activation of F4/80^+^ tumor-associated macrophages (TAMs). Additionally, NP1192 combined with anti-PD-L1 blockade also significantly reduced cancer-promoting Tregs (CD4^+^CD25^+^FOXP3^+^), compared to the anti-PD-L1 or NP1192 alone groups. Concurrent administration of NP1192 and an anti-PD-L1 antibody resulted in a consistent decrease in the proportion of M2 macrophages (CD11b^+^F4/80^+^CD206^+^), indicating a shift toward M1 macrophage polarization. Notably, NP1192 monotherapy also significantly reduced myeloid-derived suppressor cells (MDSCs) (CD11b^+^LY-6G^+^), while its effect was less pronounced in combination treatments (Figure 7A).

**Figure 7:**
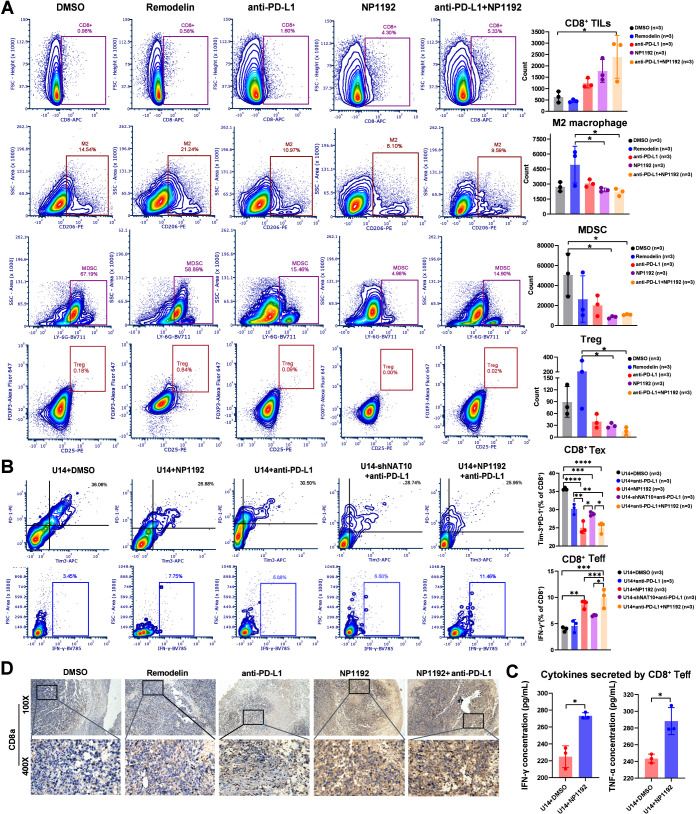
**Synergistically targeting NAT10 with NP1192 enhances the efficacy of anti-PD-L1 blockade therapy by ameliorating immunosuppressive TME**. (A) Immunoflow cytometry assays detecting the counts of multiple immune cell types, including CD8^+^ TILs, M2 macrophages, MDSCs and Treg cells. The gating strategies for selecting the above-mentioned cells and cell counts are also shown. (B–C) Immunoflow cytometry assays and gating strategies for detecting the proportions of two CD8^+^ TIL subtypes: T_eff_ and T_ex_ cells (B). The concentrations of IFN-γ and TNF-α secreted by the control and NP1192-treated coculture groups were also measured (C). (D) Representative images from IHC experiments showing the expression of CD8a in tumor tissues from xenograft model mice subjected to various treatments after treatment for 1 week. The data represent the means ± SEM of triplicate samples. *P < 0.05, **P < 0.01, ***P < 0.001, and ****P < 0.0001.

To further ascertain whether NP1192 exerts analogous modulatory effects on CD8^+^ T-cell subsets *in vitro* as observed *in vivo*, CD8^+^ TILs were isolated from the spleens of immunocompetent mice and then activated with anti-CD3/CD28 and interleukin-2 (IL-2) for 24 h. These activated CD8^+^ T cells were cocultured with U14 cells at a ratio of 1:10, exposed to various treatments for 36 h. In cultures treated with NP1192 and anti-PD-L1 antibody, IFN-γ-producing effector-like T cells (Ifn-γ^+^ CD8^+^ T_eff_ cells) dominated the CD8^+^ T-cell population, whereas terminally T_ex_ cells expressing markers such as PD-1 and Tim-3 were minimally represented, with efficacy even surpassing that of the NAT10-knockdown and anti-PD-L1 group. Additionally, combined NP1192 and anti-PD-L1 treatment resulted in enhanced cytotoxicity of CD8^+^ T cells compared to either agent alone (Figure 7B). At the end point of coculture, we separately harvested the adherent U14 and U14-shNAT10 tumor cells and subsequently performed CFSE-based cytotoxicity,^60,61^ Calcein AM/PI-costaining,^62,63^ and CCK8 assays,^64^ as described in the literatures, to visually and quantitatively assess the NP1192-mediated enhancement of CD8^+^ T cell killing capacity. CFSE-labeled tumor cell assay confirmed the addition of NP1192 or *NAT10* KD separately combined with anti-PD-L1 blockade both induced suppression of U14 cell proliferation mediated by CD8^+^ TILs. The combination of anti-PD-L1 therapy with NP1192 exhibits superior tumoricidal activity compared to NP1192 monotherapy (Figure S3D). Similarly, the confocal images of Calcein AM/PI-costained tumor cells indicated significant cell death in U14 cells cultured with coadministration of anti–PD-L1 antibody and NP1192. U14 cells treated with *NAT10* KD and anti-PD-L1 or NP1192 alone also presented CD8^+^ T-cell-mediated tumor cell killing efficacy (Figure S3E–F). ELISA analysis of the supernatant revealed significantly higher levels of IFN-γ and TNF-α in the NP1192-treated cocultures (Figure 7C), correlating with the changes in gene expression from scRNA sequencing (Figure S3C). Immunohistochemical staining of CD8a in xenograft models confirmed that both NP1192 monotherapy and combination therapy with anti-PD-L1 significantly increased the proportion of CD8^+^ T cells in the TME (Figure 7D). These findings demonstrate that NP1192, when combined with anti-PD-L1 blockade, enhances the CD8^+^ T_eff_ cell population, reprograms the TME, and potentiates antitumor immunity, suggesting its potential for clinical application in cancer immunotherapy.

## Discussion

Harnessing the therapeutic advantages of PROTACs, including their ability to overcome drug resistance, improve target specificity, and reduce off-target toxicity, we developed a series of NAT10-directed PROTAC degraders based on the structure of conventional NAT10 inhibitor Remodelin. Among these, NP1192 exhibited the most potent activity, effectively degrading NAT10 and inhibiting its ac4C acetylation function *in vitro* and *in vivo*. Compared with Remodelin, NP1192 demonstrated superior pharmacokinetics, greater NAT10 depletion, and stronger antiproliferative and anti-invasive effects.

Dysregulated PD-L1 expression, often driven by an immunosuppressive TME, is a key mechanism of resistance to PD-1/PD-L1 blockade.^65–68^ Hypoxia induces PD-L1 via HIF-1α, compromising immunotherapy efficacy.^55^ NP1192 significantly suppressed HIF-1α expression by inhibiting NAT10-mediated ac4C acetylation, leading to reduced PD-L1 protein levels. Mechanistically, NP1192 impaired HIF-1α mRNA stability and translation, thereby disrupting hypoxia-driven glycolysis by reducing glucose uptake and lactate production. *In vivo* immunohistochemistry confirmed marked reductions in HIF-1α and PD-L1 in NP1192-treated and combination therapy groups, underscoring NP1192’s capacity to counteract hypoxia-induced immune evasion and enhance antitumor immunity.

Extensive evidence suggests that hypoxic conditions drive T-cell dysfunction and exhaustion while fostering macrophage polarization toward the immunosuppressive M2 phenotype, thereby reinforcing a protumorigenic niche and diminishing the therapeutic efficacy of immunotherapy.^69,70^ Our previous research revealed that suppression of NAT10 inhibits malignant progression and synergizes with PD-L1 checkpoint blockade by reprogramming glycolytic metabolism and ameliorating immunosuppression in CCa.^9^ The literature also shows that NAT10 is highly expressed in various cancers and regulates the tumor cell cycle, metabolism, and immune microenvironment through ac4C modification. Its regulation of PD-L1 is consistent with the mechanism by which NP1192 inhibits PD-L1 in this study. Additionally, the proposed strategy of targeting NAT10 and research on Remodelin combined with anti-PD-L1 blockade provides a theoretical basis and reference for the development of NP1192 and its application in combination with anti-PD-L1 blockade to overcome immune resistance.^10^ Building upon these insights, we implemented a combinatorial therapeutic approach incorporating NP1192 with anti-PD-L1 antibody therapy. This regimen significantly enhanced intratumoral CD8^+^ TILs infiltration and upregulated IFN-γ secretion—both hallmarks of effective anti-PD-L1 immunotherapy responses.^71,72^ Notably, CD8^+^ T_eff_ cells, characterized by robust cytotoxicity and immunomodulatory functions, were enriched to the greatest extent in the combination group. In contrast, T_ex_ cells, marked by functional impairment and sustained expression of inhibitory receptors, were markedly diminished. Although the transcriptional regulation governing the CD8^+^ T_eff_ and T_ex_ lineages remains incompletely understood, it constitutes a key focus of our ongoing investigations.

In addition, combined NP1192 and anti-PD-L1 therapy induced superior suppression of immunosuppressive cellular subsets, including Tregs and MDSCs, and facilitated macrophage repolarization from the M2 to the M1 phenotype, exceeding the effects of monotherapies. These findings underscore NP1192’s capacity to mitigate hypoxia and glycolytic reprogramming, thereby reshaping the TME and restoring sensitivity to immune checkpoint blockade. High-throughput transcriptomic and single-cell RNA sequencing further corroborated these immunomodulatory effects. NAT10 degradation by NP1192 reprogrammed the immune landscape of xenografted CCa tumors, promoting a notable expansion of M1 macrophages and CD8^+^ T_eff_ cells, coupled with a reduction in MDSCs, M2 macrophages, and T_ex_ cells. These findings were validated by *in vitro* coculture assays and highlight the potential of NP1192 to reverse immune tolerance and promote durable antitumor immunity, affirming its translational promise as an immunotherapeutic agent.

This study also addresses resistance to PD-L1 inhibitors by targeting the NAT10/HIF-1α/PD-L1 axis to reshape the hypoxic TME. The PROTAC degrader NP1192 disrupts ac4C modification of HIF-1α mRNA, suppressing PD-L1 upregulation and reversing immunosuppression. In syngeneic models, NP1192 combined with anti-PD-L1 therapy synergistically inhibits tumor growth, reduces lactate production, and enhances CD8^+^ effector T cell function while decreasing the number of exhausted T cells. These findings establish a dual metabolic-immune strategy to overcome resistance to checkpoint blockade. With potent efficacy *in vitro*, *in vivo*, and across tumor organoids, and a favorable safety profile, NP1192 holds promise for treating patients refractory to immunotherapies by reprogramming both glycolytic metabolism and immune cell dynamics in hypoxic tumors.^73,74^

Despite the robust antineoplastic efficacy demonstrated by NP1192 in both *in vitro* and *in vivo* models, certain limitations remain. For example, its degradation potency necessitates further refinement. To address this issue, structural modifications should be pursued, such as exploring more diverse linkers to investigate their impact on degradation activity. In addition, adopting E3 ligase ligands beyond CRBN, such as VHL, is also a feasible strategy for the future exploration.

## Conclusions

This study underscores the therapeutic promise of NP1192 as an innovative NAT10 degrader with superior efficacy, relative to Remodelin. It demonstrates dual inhibition of hypoxia-driven glycolysis and immunosuppression via NAT10/HIF-1α/PD-L1 axis disruption, achieving superior antitumor efficacy and synergizing with anti-PD-L1 therapy. Notably, the combinatorial regimen of NP1192 with anti-PD-L1 blockade significantly reshapes the immune cell repertoire, especially boosting CD8^+^ T_eff_ function and T_ex_ suppression within the TME. This dual-action strategy holds transformative potential for overcoming metabolic-immune dysfunction in hypoxic tumors, warranting clinical translation for precision immuno-oncology.

## Methods

### Synthesis of NAT10 PROTACs

The detailed synthetic procedures, NMR spectra, and characterization of the **NAT10 PROTACs** as well as other materials and methods are described in the Supporting Information and Methods.

### Cell Culture

The human CCa cell line SiHa and the normal epithelial foreskin fibroblast line HFF-1 were purchased from the Cell Bank of the Chinese Academy of Sciences. Additionally, murine cervical carcinoma cell line U14 (RRID:CVCL_9U56) was purchased from Boster Company. HFF-1 and U14 cells were cultured in Dulbecco’s modified Eagle’s medium (DMEM) (Gibco, USA) supplemented with 10% heat-inactivated fetal bovine serum (FBS; Gibco, USA), while SiHa cells were incubated in RPMI 1640 medium (Gibco, USA) in a humid atmosphere with 5% CO_2_ at 37 °C. For hypoxia exposure, the cells were incubated in a sealed culture tank containing 5% CO_2_ and 0.1% O_2_ (MGC, Japan) for 36 h.

### Animal Procedures

#### Murine Cervical Cancer Cell Line-Derived Mouse Xenograft Model

Female C57BL/6J mice aged 6–8 weeks were purchased from the Laboratory Animal Center of Guangdong Medical Laboratory Animal Center.

##### For Experiment 1 (Generation of Growth Curves and Imaging of Tumors)

U14 cells (1 × 10^7^ cells per mouse, approximately 100–150 μL of cell suspension) were implanted subcutaneously into the lateral lower dorsal surface of each mouse. The tumors were allowed to grow for 7 days, and the mice were then administered one of five treatments (group 1: U14 cells + DMSO; group 2: U14 cells + Remodelin; group 3: U14 cells + anti-PD-L1 antibody; group 4: U14 cells + NP1192; group 5: U14 cells + anti-PD-L1 antibody + NP1192). Each group of mice was assigned according to the principles of randomization and the blinding method. Tumors were measured every 2 days, and tumor volume was calculated via the following equation: volume = (width)^2^ × length/2. Remodelin and NP1192 were injected into the peritumoral area every 2 days at a dose of 25 mg/kg, and the *in vivo* antimouse PD-L1 antibody (low endotoxin) was injected every 3 days at a dose of 2.5 mg/kg. After 1 week of treatment, the mice were euthanized when the total tumor burden approached guidelines and the tumor burden did not exceed 20 mm in diameter, the tumors were removed and weighed, and the tumors and peritumoral tissues were subjected to assays of the immune microenvironment.

##### For Experiment 2 (*In Vivo* Imaging)

A lentiviral vector expressing firefly luciferase was transduced into the U14 cells. After puromycin selection and cell expansion, U14 cells with stable firefly luciferase expression were generated. C57BL/6J mice were randomly divided into the following 5 groups for further experiments: group 1, U14 cells + DMSO; group 2, U14 cells + Remodelin; group 3, U14 cells + anti-PD-L1 antibody; group 4, U14 cells + NP1192; and group 5, U14 cells + anti-PD-L1 antibody + NP1192. After tumor stabilization, the luciferase substrate was injected into the mice every 2 days, and fluorescence values were obtained via a Maestro *in vivo* imaging system.

##### For Experiment 3 (Immunoflow Cytometry)

After the mice were euthanized, the tumor and peritumoral tissues were analyzed with an immunoflow kit (BioLegend, USA). The antibodies used for immunoflow cytometry included antihuman/mouse antibodies specific for CD11b-BV711, F4/80-AF647, CD206-PE, CD86-BV605, CD4-CD4-FITC IL-17-17-CY7/CY7, CD25-PE, FOXP3-AF647, CD4-BV605, CD45-PE/CY7, CD11b-BV711 and LY-6G/LY-6C-BV421 (BioLegend catalog no. 321239, RRID:AB_2860843). All data analyses were performed via GraphPad Prism 9, and the data are presented as the mean ± SEMs.

#### Pharmacokinetic Study in Male SD Rats

Male SD rats (180–220 g) were purchased from Beijing Vital River Laboratory Animal Technology Co., Ltd. Blood samples (0.25 mL) were collected from the tail vein at 0.0833, 0.25, 0.5, 1, 2, 4, 6, 8, and 24 h after oral (10 mg/kg) or intravenous (2 mg/kg) administration of compound NP1192. Drug concentrations in the samples were determined by using liquid chromatography–mass spectrometry (LC-MS/MS). The mobile phase consisted of a mixture of solvent A (acetonitrile) and solvent B (formic acid/ultrapure water, 1:1000, v/v). The flow rate of the mobile phase was 0.3 mL/min, and the injection volume was 10 μL.

#### Evaluate of Acute Toxicity

Six-weeks old C57BL/6J male mice were purchased from Guangzhou Southern Medical University Experimental Animal Technology Development Co. The mice were administered intraperitoneally NP1192 (100, 500, 1000 mg/kg). The survival of mice was monitored for 14 days, consecutively.

### Molecular Docking

Molecular docking studies were performed with the induced fit docking (IFD) module of the Schrödinger software package (Maestro 11.1) according to previous methods. The three-dimensional coordinates of the human NAT10 protein were downloaded from the AlphaFold protein structure database (https://AlphaFold.ebi.ac.uk/entry/Q9H0A0). The protein structure was prepared via the Protein Preparation Wizard by repairing the missing residues, adding hydrogen atoms, removing water molecules, and minimizing the structure. The 3D structure of Remodelin was constructed in Maestro and further optimized with the LigPrep panel. The binding sites in each protein were set as a 30 Å × 30 Å × 30 Å box in the center of Ile629, Ala630, Val631, Gln636, Gly637, Met638, Gly639, Tyr640, Gly641, Ser642, Leu719, Phe722, and Arg725. After the initial Glide docking, the ligand and the side chains within 5.0 Å of the ligand in each initial pose were optimized via Prime refinement. Next, extra-precision (XP) docking was employed to redock Remodelin into the binding site of each receptor. The conformer with the best score was determined and visualized in PyMOL.^75^

### Transfection

Two gRNA sets were designed and subsequently cloned and inserted into the vector LV-U6 > {gRNA-A1}-U6 > {gRNA-A2}-CMV > P2A/Hygro (Cyagen, Guangzhou). The CRISPR–Cas9 gene editing system by Haixing Biosciences (Guangzhou, China) was used to generate a cell line with stable heterozygous knockdown of NAT10 (NAT10^±^ SiHa cells). The sequences of the NAT10 sgRNAs used were as follows: sgRNA-1, ACTGCA­CGGATA­GCAA­GTGG; and sgRNA-2, CCAAA­GGAAG­ATAAT­GCACAA. Approximately 10 single-cell-derived clones were established and confirmed via polymerase chain reaction (PCR). The heterozygous NAT10-knockdown SiHa cell line (NAT10^±^) and negative control (NC) cell line were further confirmed by Western blotting for NAT10.

### Establishment of Drug-Resistant Strains and Detection of RI

We generated Remodelin-resistant (Remodelin^Resist^ SiHa) and NP1192-resistant (NP1192^Resist^ SiHa) SiHa CCa cell lines by applying a stepwise dose escalation strategy, as previously described in the literature.^21^ To quantitatively assess resistance, we calculated the resistance index for each subline following the established methodologies. Briefly, 3000 cells per well were seeded in 96-well plates and treated with increasing concentrations of Remodelin or NP1192 (0, 10, 50, 100, and 150 μM) for 24 h. Following treatment, 10 μL of CCK-8 reagent was added to each well and incubated for an additional 2 h. Absorbance at 490 nm was measured, and the IC_50_ values were calculated using GraphPad Prism 9.0. The RI was defined as the ratio of the IC_50_ in resistant cells to that in parental cells.

### ac4C Dot Blot

RNA was extracted from cells via an RNA extraction kit (TransGen, China) and denatured by heating to 65 °C for 5 min in the presence of 2× RNA denaturation buffer (Solarbio, China) followed by an equal amount of 20x SSC buffer (Beyotime, China). A nylon membrane (Yeasen, China) was used to immobilize the denatured RNA by dotting RNA samples onto the membrane and cross-linking the RNA to the membrane via UV light. The samples were stained with methylene blue and photographed. Furthermore, the membrane was blocked with 5% milk and incubated with an anti-ac4C primary antibody (1:1000, Abcam, US) for 12 h. After the unbound primary antibody was washed away, the membrane was incubated with a secondary antibody (1:6000, Elabscience, China). The membrane was then washed to remove unbound secondary antibodies, and a detection reagent (Bio-Rad, US) was added to visualize the RNA bands via chemiluminescence with a ChemiDoc MP Imaging System (Bio-Rad, US).

### Generation of Patient-Derived Organoids (PDOs)

Organoid culture was performed according to previously established protocols.^24^ Briefly, we procured patient-derived tissue samples provided and technically manipulated by Accurate International Biotechnology Co., Ltd. Subsequently, these samples were subjected to mechanical and enzymatic dissociation to disaggregate the tissue into single cells or small clusters, while preserving cellular integrity. Following this initial step, the isolated cells or tissue fragments were embedded within a 3D ECM scaffold, such as Matrigel or collagen. The ECM scaffold served as a supportive substrate, promoting cellular self-organization and mimicking native tissue architecture. Additionally, a precisely formulated cocktail of growth factors, cytokines, and signaling molecules tailored to the specific tissue of interest was meticulously formulated and added to the culture medium to facilitate organoid growth and maintenance.

### Isolation of CD8^+^ T Cells and Coculture with Tumor Cells

The mice were euthanized by cervical dislocation, and the entire spleen was removed after being moistened/soaked in 75% alcohol. The spleen was placed in a 70 μm mesh sieve positioned over a 50 mL tube. A certain amount of 1× PBS buffer was poured into the sieve, and the spleen was ground with a syringe plunger. The ground cell suspension was then added to 20 mL of 1× PBS and centrifuged at 350 × g for 5 min, after which the supernatant was discarded. Fresh CD8^+^ T cells were extracted according to the instructions of the kit. The day before the CD8^+^ T cells were extracted, the antimouse CD3 antibody was diluted to 5 μg/mL with sterile PBS, and the diluted antibody was added to a 24-well plate and incubated overnight at 4 °C. (Before the antibody was added to the plate, the plate was rinsed with sterile PBS 2–3 times to avoid dry wells). The plate incubated the previous day was removed from the 4 °C refrigerator on the second day, and the coating solution was discarded, followed by washing with sterile PBS 3 times. Amplification culture medium consisting of DMEM + 10% serum + 5 μg/mL antimouse CD28 + IL-2 was prepared. The cell lines used for culture were maintained in the logarithmic growth phase (70–80% confluence). Cell counting was performed, and CCa cells were seeded in a 12-well plate (10^5^ cells/well) and cultured until adherence. T cells were mixed with tumor cells at different effector-to-target ratios (1:10) and then incubated with different drugs for 24 h. Flow cytometry experiments were then performed to detect the CD8^+^ T-cell subtypes. At the end point of coculture, we also separately harvested the adherent U14 and U14-shNAT10 tumor cells and subsequently performed CFSE-based cytotoxicity,^60,61^ Calcein AM/PI-costaining,^62,63^ and CCK8 assays,^64^ as described in the literatures, to visually and quantitatively assess the NP1192-mediated enhancement of CD8^+^ T cell killing capacity.

### Single-Cell Sequencing (scRNA-seq)

Preimplanted CCa tissue samples from C57BL/6J mice were utilized. The tissue samples were sliced into small pieces (approximately 1 mm^3^) in PBS to maintain cell integrity. The tissue blocks were digested via appropriate enzymes (such as collagenase) at 37 °C for 30 min to obtain a cell suspension, which was then filtered through a 70 μm cell filter to remove residual large tissue fragments, yielding a single-cell suspension. The single-cell suspension was adjusted to the appropriate cell concentration according to the experimental requirements to ensure the capture of a sufficient number of single cells. Using a single-cell capture platform such as a microfluidic chip, the adjusted cell suspension was loaded into capture channels. The single-cell capture platform was initiated to capture and separate single cells through microchannels, ensuring capture efficiency and cell integrity. Single cells were successfully captured via lysis buffer, while preserving RNA integrity. Subsequent procedures, including RNA extraction, cDNA synthesis, library construction, sequencing, and data analysis, were conducted following the steps previously described for RNA-seq.

### Quantitative Reverse Transcription Polymerase Chain Reaction (qRT–PCR)

TRIzol reagent (TransGen, China) was used to extract total RNA from SiHa and U14 cells according to the manufacturer’s protocol. HiScript II (Vazyme, China) was used to synthesize cDNA. Fluorescence quantitative PCR was performed with SYBR Green Master Mix (Vazyme, China) on an Applied Biosystems 7500 sequence detection system (Thermo Fisher, US). Consistent thermal cycling conditions were used: 95 °C for 30 s, followed by 40 cycles at 95 °C for 10 s and 60 °C for 30 s. Relative RNA expression levels for each gene were calculated according to the 2^–ΔΔ^ CT method, with GAPDH as the internal control. All sequences of the primers used for *HIF1A* and PD-L1 are listed in Supplementary Table S2.

### Western Blot Analysis

RIPA lysis buffer (Beyotime, China) mixed with 1% protease inhibitor (Sigma, US) was added to the cells, and a sample of total protein was obtained and then loaded onto an 8% or 12% polyacrylamide gel. The gel was placed in a buffer solution, and an electric current was applied. The separated proteins were subsequently transferred from the gel onto a PVDF membrane. Next, the membrane was blocked with 5% milk for 1 h to prevent nonspecific antibody binding. The membranes were subsequently incubated with anti-NAT10 (1:1000, Abcam, USA), anti-PD-L1 (1:1000, Proteintech, China), anti-HIF-1α (1:1000, Abmart, China), and anti-β-tubulin (1:1000, Proteintech, China) primary antibodies for 12 h. Afterward, the membrane was sequentially washed with 1× TBST and incubated for 1 h with an antimouse secondary antibody (1:6000, Elabscience, China) or antirabbit secondary antibody (1:6000, Elabscience, China). The target proteins were detected via chemiluminescence with a ChemiDoc MP Imaging System (Bio-Rad, US).

### Immunohistochemical (IHC) Staining

Tumor tissue samples from C57BL/6J mice were first fixed with formalin and embedded in paraffin. Sections of the tissues were then cut via a microtome and placed on glass slides. The tissue sections were immersed in a xylene solution and gradient concentrations of ethanol for dewaxing and then boiled in EDTA antigen repair solution (pH = 9) at a high temperature for approximately 20 min. The tissue sections were treated with blocking solution (Zsbio, China) for 10 min to prevent nonspecific binding of the following primary antibodies during incubation for 12 h. The primary antibodies used were anti-NAT10 (1:1500, Abcam, USA), anti-PD-L1 (1:1500, Proteintech, China), and anti-HIF-1α (1:500, Abmart, China). The tissue sections were incubated with a secondary antibody (Zsbio, China) for 1 h and DAB enzyme substrate (Zsbio, China), which produced a visible signal. The tissue sections were subsequently counterstained with hematoxylin to visualize the tissue structure. Hydrochloric acid alcohol (1%) was added, and the samples were rinsed with tap water for more than 20 min to reestablish a blue color. Next, the tissue sections were immersed two times in 75% ethanol and dried, and a drop of neutral gum was added to seal the coverslips. An upright microscope and the associated equipment were used to image the sections at magnifications of 50× and 200× (Leica, Germany).

### Cell Counting Kit-8 (CCK-8) Assay

NAT10^±^ SiHa cells and SiHa cells treated with DMSO, Remodelin or NP1192 for 36 h were seeded into the wells of a 96-well microplate at identical densities of 2000 or 3000 cells per well. After the cells adhered to the well surface, 110 μL of a 1:10 mixture of CCK-8 reagent (Meilunbio, China) and RPMI 1640 medium was added to each well. The microplate was then incubated for another 2 h in the incubator, and the mixture was replaced with an equal volume of medium. A microplate reader (BioTek, US) was used to measure the absorption at a wavelength of 450 nm.

### Colony Formation Assay

NAT10^±^ SiHa cells and SiHa cells treated with DMSO, Remodelin or NP1192 for 36 h were seeded into the wells of a 6-well microplate at identical densities of 2000 or 3000 cells per well. The cells were then incubated in the incubator for at least 2 weeks to form colonies. After the incubation period, the cells were fixed with formalin and then stained with crystal violet. The colonies were then counted via a microscope. The number and size of the colonies in the experimental groups were compared.

### Transwell Invasion Assay

The Transwell chamber was assembled by coating the porous membrane insert in the top compartment with Matrigel to form a layer of extracellular matrix (ECM), and the top compartment was placed into a cell culture plate. NAT10^±^ SiHa cells and SiHa cells treated with DMSO, Remodelin or NP1192 for 36 h were collected and resuspended in serum-free medium at a density of 10,000 cells/200 μL of medium. Then, the cell suspension was added to the top compartment of the Transwell chamber, and the plate was placed in the incubator for 12 h. After incubation, the cells on the bottom surface of the membrane were fixed and stained. The cells were visualized under a microscope, and the number of cells that migrated through the membrane was determined via ImageJ software.

### Flow Cytometry Analysis of the Cell Cycle

NAT10^±^ SiHa cells and SiHa cells treated with DMSO, Remodelin or NP1192 for 36 h were harvested and fixed with 70% absolute ethanol for 12 h. The fixed cells were then treated with a solution containing the fluorescent DNA-binding dye PI and RNase A in a light-protected environment (Yeasen, China). The stained cells were analyzed via flow cytometry. The data collected from the flow cytometer were analyzed via FlowJo software. The cell cycle distribution was calculated based on the DNA content of the cells.

### RNA Sequencing (RNA-seq)

Total RNA was extracted from SiHa cells treated with NP1192 or DMSO. Then, RNA-seq was conducted by Oebiotech Company (Shanghai, China) on the Illumina (San Diego, CA, USA) platform. Next, a sequencing quality assessment and genome alignment were performed. The expression of all the genes was quantitatively analyzed by calculating the fragments per kilobase million values, and the DEGs were identified as those with a |log_2_(fold change)| > 1 and a *q* value <0.005. Finally, the functions of the DEGs were investigated via gene set enrichment analysis (GSEA), Gene Ontology (GO) analysis and Kyoto Encyclopedia of Genes and Genomes (KEGG) pathway analysis. Ribo-seq and acRIP-seq analyses were performed according to the manufacturer’s instructions.^9^

### mRNA Stability Assay

SiHa cells and *NAT10-*KD SiHa cells (2 × 10^5^ cells per well) were maintained in six-well plates in RPMI 1640 medium supplemented with 10% FBS. After the cells had adhered to the plate, 5 μM actinomycin D (Yeasen, China) was added, and the plate was incubated for 0, 4, or 8 h. Changes in the *HIF1A* mRNA half-life were subsequently evaluated via qPCR.

### Nascent Protein Synthesis Assay

*NAT10*-KD SiHa cells and SiHa cells treated with DMSO or NP1192 for 36 h (2 × 10^5^ cells per well) were seeded into six-well plates in RPMI 1640 medium supplemented with 10% FBS. When the cell density reached 70–80%, the cells were fixed with methanol for 20 min, dyed with Click-iT Plus OPP Protein Synthesis Assay Kits (Invitrogen; US) according to the manufacturer’s instructions, and stained with DAPI for 20 min at room temperature. Finally, the cells were observed under a confocal laser scanning microscope.

### Lactic Acid Assay, ATP Assay, and 2-NBDG Uptake Assay

SiHa and U14 cells (2 × 10^5^ cells per well) were plated in 6-well plates and cultured in RPMI 1640 medium or DMEM supplemented with 10% FBS and 1% penicillin–streptomycin. After cell adherence, 20 μM NP1192 or DMSO was added and incubated for 36 h, and the assays were performed according to the instruction manuals for the Lactate Assay Kit (Nanjing Jiancheng, China), ATP Assay Kit (Beyotime, China), and 2-NBDG reagent (GlpBio, US). All data analyses were performed via GraphPad Prism 9, and the data are presented as the means ± SEMs.

### Seahorse Assay

SiHa and U14 cells (2 × 10^5^ cells per well) were seeded in six-well plates and cultured in RPMI 1640 medium or DMEM supplemented with 10% FBS and 1% penicillin–streptomycin. After cell adherence, 20 μM NP1192 or DMSO was added, and the mixture was incubated for 36 h. The assay procedure was performed according to the instruction manual for the Seahorse XF Glycolysis Test Kit (Agilent, USA). All data analyses were performed via GraphPad Prism 9, and the data are presented as the means ± SEMs.

### JC-1 Staining Assay

SiHa and U14 cells (2 × 10^5^ cells per well) were seeded in six-well plates and maintained in RPMI 1640 medium or DMEM supplemented with 10% FBS and 1% penicillin–streptomycin. After cell adherence, 20 μM NP1192 or DMSO was added, and the mixture was incubated for 36 h. The assay was performed according to the instruction manual for the mitochondrial membrane potential detection kit (Beyotime, China). All data analyses were performed via GraphPad Prism 9, and the data are presented as the means ± SEMs.

### ROS Staining Assay

SiHa and U14 cells (2 × 10^5^ cells per well) were seeded into 6-well plates and cultured in RPMI 1640 medium or DMEM supplemented with 10% FBS and 1% penicillin–streptomycin. After cell adherence, 20 μmol of NP1192 or DMSO was added and incubated for 36 h, and the assay was performed according to the instruction manual for the ROS Assay Kit (Beyotime, China). All data analyses were performed via GraphPad Prism 9, and the data are presented as the means ± SEMs.

### ELISA

Fresh tumor tissues were harvested from C57BL/6J mice and immersed in PBS. A pulverizer was inserted into each tumor sample, which was run at high speed for 30 s for three cycles to crush the tissue. The tissue lysate was centrifuged at 12000 rpm for 10 min, and the upper layer was collected. Subsequent steps were performed according to the manufacturer’s instructions for the mouse gamma interferon (IFN-γ) ELISA test kit (Jiangsu Meibiao Biotechnology, China). All data analyses were performed via GraphPad Prism 9, and the data are presented as the means ± SEMs.

### Statistical Analysis

All of the statistical analyses were conducted via GraphPad Prism version 9.0. The results are presented as means ± standard errors of the means (SEMs). The gray values, colony formation data, and Transwell statistics were computed with ImageJ software. Group comparisons were evaluated via Student’s *t* test and ANOVA. Specifically, one-way ANOVA was employed for analyses involving three or more groups, ANOVA and Welch’s test were applied for data sets exhibiting heterogeneity in variance, and nonparametric tests were utilized for nonnormally distributed data. Statistical significance was set at a threshold of *P* < 0.05.

### Safety Statement

No unexpected or unusually high safety hazards were encountered.

## Supplementary Material



## Data Availability

The data generated in this study are publicly available in the Gene Expression Omnibus (GEO) at GSE216812 [acRIP-seq].

## Notes

Figure S1. The drug plasma concentration–time curve of NP1192. Figure S2. Body weight changes of mice after administration of NP1192. Figure S3. Additional RNA-seq and scRNA-seq data, as well as CD8^+^ TILs killing ability certification. Table S1. *In vivo* pharmacokinetic properties of NP1192. Table S2. Primers used for qPCR detection. Supporting Information and Methods. The detailed synthetic procedures, NMR spectra, and characterization of the NAT10 PROTACs. (PDF)

## Abbreviations

NAT10: N-acetyltransferase 10
PROTACs: proteolysis-targeting chimeras
UPS: ubiquitin-proteasome system
CCa: cervical cancer
ICB: immune checkpoint blockade
TME: tumor microenvironment
CSCs: cancer stem cells
ac4C: RNA N4-acetylcytidine
IC_50_: half maximal inhibitory concentration
Ribo-seq: ribosome profiling sequencing
acRIP-seq: acetylated RIP sequencing
scRNA-seq: single-cell RNA sequencing
PDOs: patient-derived organoids
ATP: adenosine triphosphate
ECAR: extracellular acidification rate
Treg: regulatory T
HIF-1α: hypoxia-inducible factor 1α
PD-L1: programmed cell death ligand 1
PD-1: programmed cell death 1
HFF-1: human epithelial foreskin fibroblasts
GSEA: gene set enrichment analysis
GO: Gene Ontology
KEGG: Kyoto Encyclopedia of Genes and Genomes
ECAR: extracellular acidification rate
IGV: Integrative Genomics Viewer
Act D: actinomycin D
TIL: tumor-infiltrating lymphocyte
T_eff_: effector CD8^+^ T cells
T_ex_: exhausted CD8^+^ T cells
TAMs: tumor-associated macrophages
MDSCs: myeloid-derived suppressor cells.
