# Supplementary material for: Targeted NAT10 Degradation by PROTAC NP1192 Suppresses Hypoxia-Adaptive Glycolysis and Reinvigorates CD8+ Effector T‑Cell Function for Synergistic Cancer Immunotherapy
Source: ACS Cent Sci. 2025 Sep 16;11(11):2087–107. doi: 10.1021/acscentsci.5c00812 (PMC12670317; doi:10.1021/acscentsci.5c00812)
Supplement: Supplementary file 1 [file oc5c00812_si_001.pdf]

Targeted NAT10 degradation by PROTAC NP1192 suppresses hypoxia-adaptive glycolysis and reinvigorates CD8$^+$ effector T-cell function for synergistic cancer immunotherapy

Keyi Ao[1,2,†], Zhiqiang Sun[3,†], Yi Hao[4,†], Jiaqi Qin[1,2,†], Chenglong Xu[3], Xiuli Wen[4], Zichao Yang[3], Li Li[1,2], Shaoyan Gan[1], Xiaona Chen[1,2], Xin Li[1,2,†,*], Jian Zhang[5,6,7,†,*], Jianjun Chen[3,†,*], Xia Guo[1,2,†,*]

[1]Shenzhen Key Laboratory of Viral Oncology, Department of Science and Innovation, Shenzhen Hospital, Southern Medical University, Shenzhen, Guangdong 518100, P. R. China

[2]Shenzhen School of Clinical Medicine, Southern Medical University, Shenzhen, Guangdong 510515, P. R. China

[3]Guangdong Provincial Key Laboratory of New Drug Screening, NMPA Key Laboratory for Research and Evaluation of Drug Metabolism, School of Pharmaceutical Sciences, Southern Medical University, Guangzhou, Guangdong 510515, P. R. China

[4]Department of Ultrasound, South China Hospital, Medical School, Shenzhen University, Shenzhen, Guangdong 518116, P. R. China

---

[†] These authors contributed equally to this work.

[*] Correspondence: Xia Guo (Ministry of Science and Innovation, Shenzhen Hospital, Southern Medical University, Shenzhen, Guangdong 518100, P. R. China, +86-0755-23360150, myshow0504@smu.edu.cn (lead contact)); Jianjun Chen (School of Pharmaceutical Sciences, Southern Medical University, Guangzhou, Guangdong 510515, P. R. China, +86-20-62789423, jchen21@smu.edu.cn); Jian Zhang (Department of Human Cell Biology and Genetics, School of Medicine, Southern University of Science and Technology, Shenzhen, Guangdong 518055, P. R. China, +86-0755-88018036, zhangjian@sustech.edu.cn); Xin Li (Ministry of Science and Innovation, Shenzhen Hospital, Southern Medical University, Shenzhen, Guangdong 518100, P. R. China, +86-0755-23360061, xinli268@gmail.com).

[5]Department of Human Cell Biology and Genetics, School of Medicine, Southern University of Science and Technology, Shenzhen, Guangdong 518055, P. R. China
[6]Joint Laboratory of Guangdong-Hong Kong Universities for Vascular Homeostasis and Diseases, SUSTech Homeostatic Medicine Institute, School of Medicine, Southern University of Science and Technology, Shenzhen, Guangdong 518055, P. R. China
[7]Clinical Research Center, the First People's Hospital of Foshan (The Affiliated Foshan Hospital of Southern University of Science and Technology), School of Medicine, Southern University of Science and Technology, Shenzhen, Guangdong, 528000, P. R. China

Supplementary Figure S1

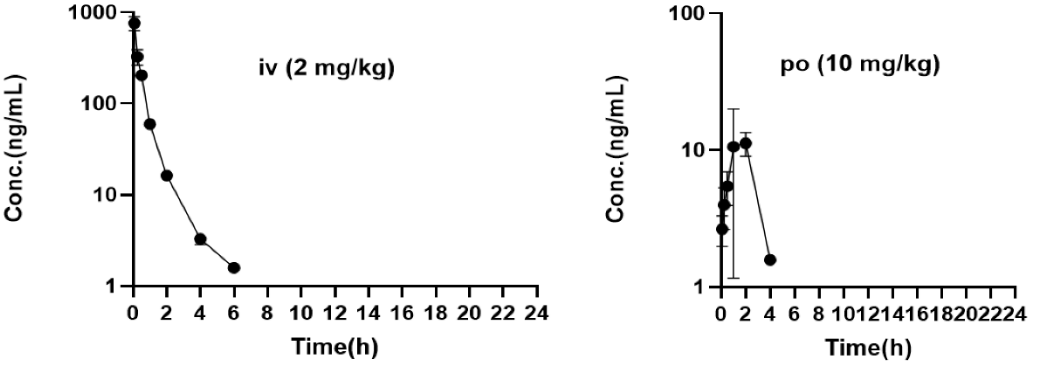

Figure S1. The drug plasma concentration-time curve of NP1192.

Supplementary Table S1

Table S1. *In vivo* pharmacokinetic properties of NP1192.

PK parameters	i.v. administration (2 mg/kg, n = 3)	p.o. administration (10 mg/kg, n = 3)
$^{\alpha}AUC_{(0-t)}$ (ng/mL·h)	365±42.6	24.7±1.11
$AUC_{(0-\infty)}$ (ng/mL·h)	367±43.0	29.5±1.59
$MRT_{(0-t)}$ (h)	0.529±0.0268	1.56±0.358
$MRT_{(0-\infty)}$ (h)	0.570±0.0206	2.85±0.862
$t_{1/2}$ (h)	0.967±0.214	2.40±1.15
T_{max} (h)	0.0833±0.00	1.67±0.577
CL (L/h/kg)	5.503±0.66	

V_z (L/kg)	7.597±1.391	
C_{max} (ng/mL)	759±134	13.9±6.42
F (%)		1.35 %

$^{\alpha}$AUC, area under the curve; CL, plasma clearance; C_{max}, maximum plasma concentration; MRT, mean residence time; $t_{1/2}$, half-life; T_{max}, time of maximum plasma concentration.

Supplementary Figure S2

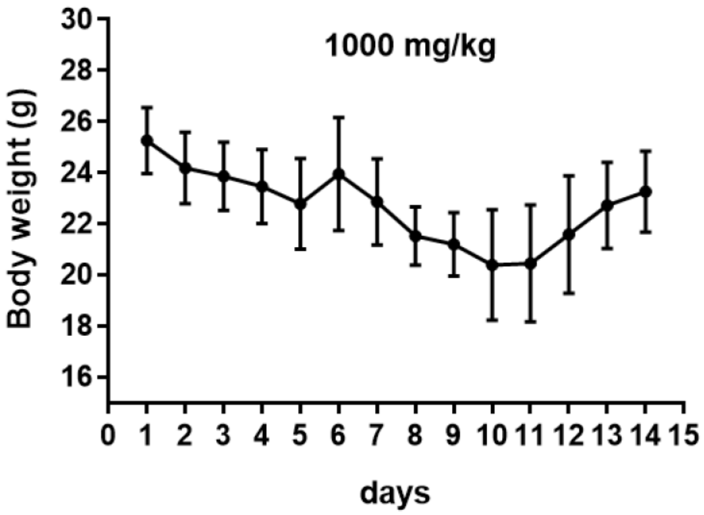

Figure S2. Body weight changes of mice (n = 5) after administration of NP1192.

Supplementary Figure S3

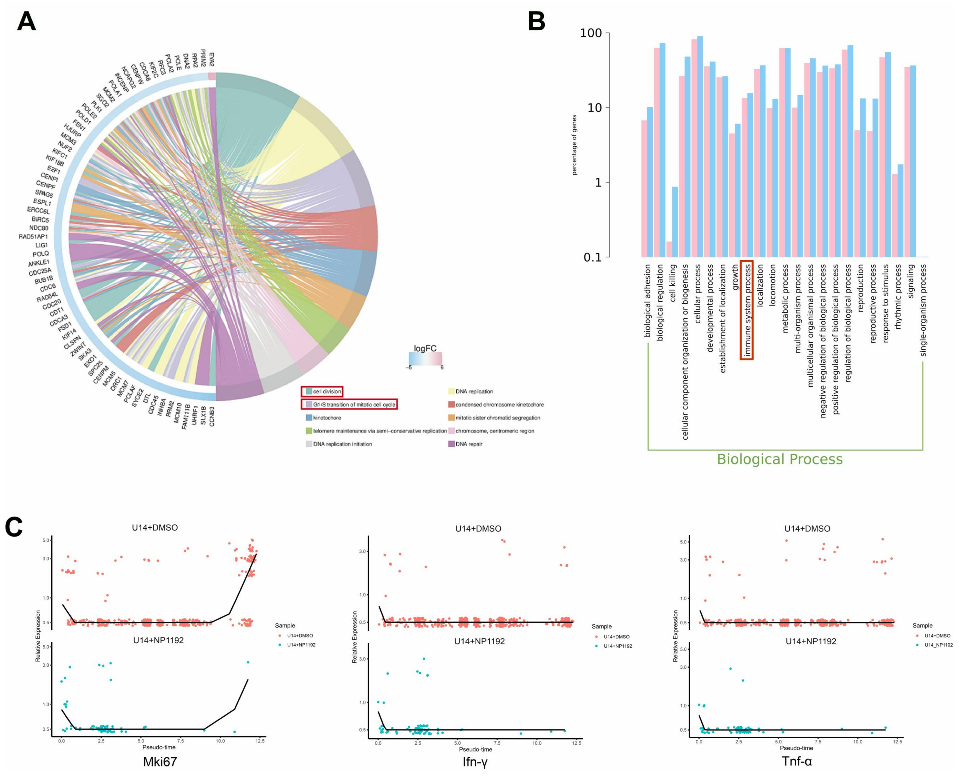

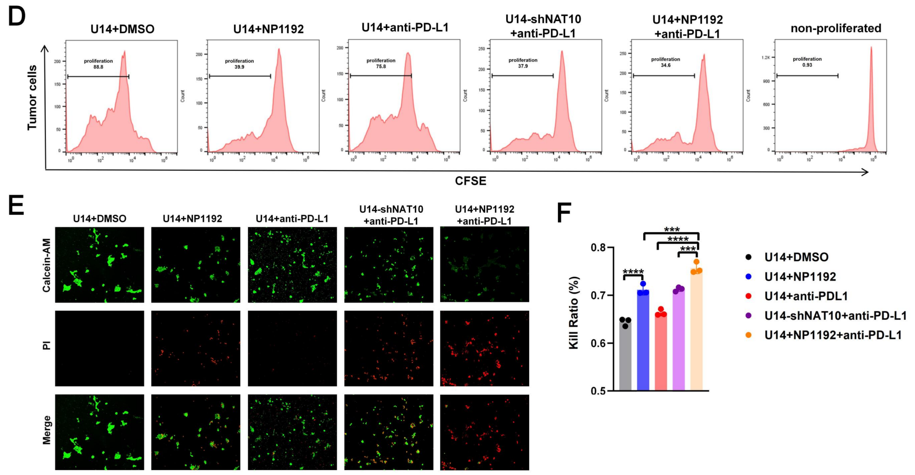

Figure S3. **Additional RNA-seq and scRNA-seq data, as well as $CD8^+$ TILs killing ability certification.** (A) GO analysis showing the enriched biological processes, including the enrichment of immune system processes and cytotoxicity-related pathways, in the transcriptome of cells treated with NP1192 at a concentration of 20 μM for 36 h. (B) Alterations in related GO terms, namely, cell division and G1/S transition of the mitotic cell cycle, according to the transcriptome sequencing data from SiHa cells treated with NP1192 at a concentration of 20 μM for 36 h. (C) Map of alterations in 3 genes (Mki67, Ifn-γ and Tnf-α) in tumor samples from the control and NP1192-treated groups during the differentiation trajectory. (D) $CD8^+$ TILs isolated from C57BL/6J mice were cocultured with CFSE-labeled U14 or U14-shNAT10 with different treatments for 36 h. Histograms show representative U14 and U14-shNAT10 cells proliferation as assessed by flow cytometny. The numbers in the plots indicate the frequency of proliferating U14 and U14-shNAT10 cells. (E) CLSM images of Calcein AM/Pl costaining of U14 and U14-shNAT10 cells after diferent treatments for 36 h. (F) $CD8^+$ T cell killing assay of U14 and U14-shNAT10 cells. After eliminating the suspended $CD8^+$ T cells, the absorbance of the remaining U14 and U14-shNAT10 cells was detected by CCK-8, and the killing ratio was calculated.

Supplementary Table S2

Table S2. Primers used for qPCR detection.

Primer	Sequence (5'-3')
GAPDH Forward	GACAGTCAGCCGCATCTTCT
GAPDH Reverse	TTAAAAGCAGCCCTGGTGAC
HIF1A Forward	GAACGTCGAAAAGAAAAGTCTCG
HIF1A Reverse	CCTTATCAAGATGCGAACTCACA
CD274 Forward	GGCACAGTTGCCGATTACAGA
CD274 Reverse	CTGCTGAACTTCACTCTCAGG
HIF1A acRIP-qPCR Forward	TTCTTCAGGAAAGGTGGCGT
HIF1A acRIP-qPCR Reverse	TGGTCAGTGTGCTTCAGAACA

Supplementary Materials and Methods

NAT10 PROTAC synthesis and NMR spectra

Unless specified otherwise, all materials were obtained from commercial sources and used without purification. Reactions were monitored by thin layer chromatography (TLC). The dynamic adsorbent silica gel F-254 was used, and spots were detected at 254 nm and 365 nm. Flash column chromatography separations were performed on silica gel (100-200 mesh). ^{1}H NMR (400 MHz) and ^{13}C NMR (101 MHz) spectra were recorded on a Bruker Avance 400 MHz NMR spectrometer with DMSO and $CDCl_3$ as the solvents. Chemical shifts are reported in ppm, and all coupling constants are reported in hertz. The purity of all final compounds (>95%) was tested via HPLC on a Shimadzu LC-20A HPLC system equipped with a UV detector. HPLC was conducted using a 5 µm C-18 column (4.6 × 150 mm) at 40 °C and a flow rate of 1 mL/min. The HPLC gradient consisted of solvent A (water) and solvent B (methanol): 0-1 min 30% B, 1-15 min 30-90% B (linear gradient), and 15-25 min 90% B.

Scheme S1. Synthesis of target compounds **TP1–TP20.**

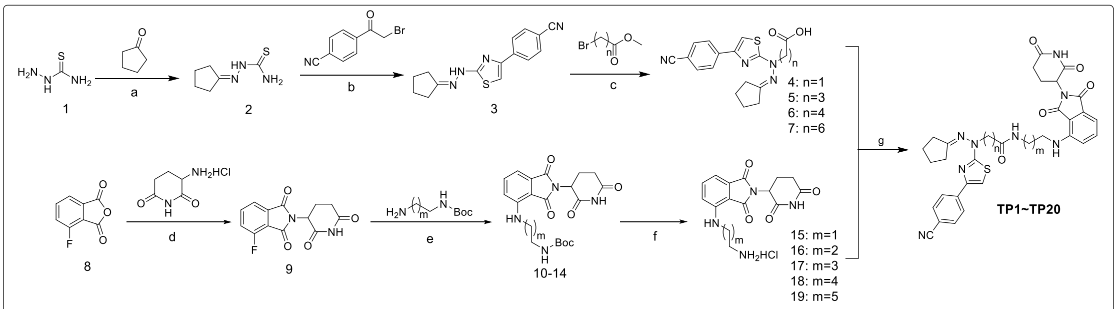

Reagents and conditions: (a) isopropanol, 82 °C; 75 %; (b) isopropanol, rt; 45 %; (c) K_2CO_3, DMF, 80 °C; NaOH, MeOH, rt; 65-80.4 %; (d) CH_3COOH, CH_3COOK, 120 °C, 5 h, 60 %;(e) DIPEA, DMF, 90 °C, 4 h, 38.2-50 %; (f) 4M HCl-dioxane solution, DCM, rt, 2 h, 62.1 %-75.7 %; (g) EDCI, HOBT, TEA, DMF, rt, 4 h, 55-61 %.

2-Cyclopentylidenehydrazine-1-carbothioamide (2)

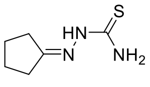

Thiosemicarbazide (1 g, 10.97 mmol) and cyclopentanone (0.92 g, 10.97 mmol) were dissolved in 30 mL of isopropanol, and the mixture was refluxed for 12 h. After the reaction was complete, the precipitate was filtered to produce intermediate 2 (1.44 g, 75% yield). White solid.

4-(2-(2-Cyclopentylidenehydrazineyl)thiazol-4-yl)benzonitrile (3)

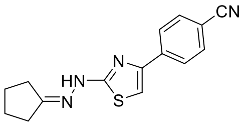

Scheme S2. Synthesis of target compounds **TP21–TP32.**

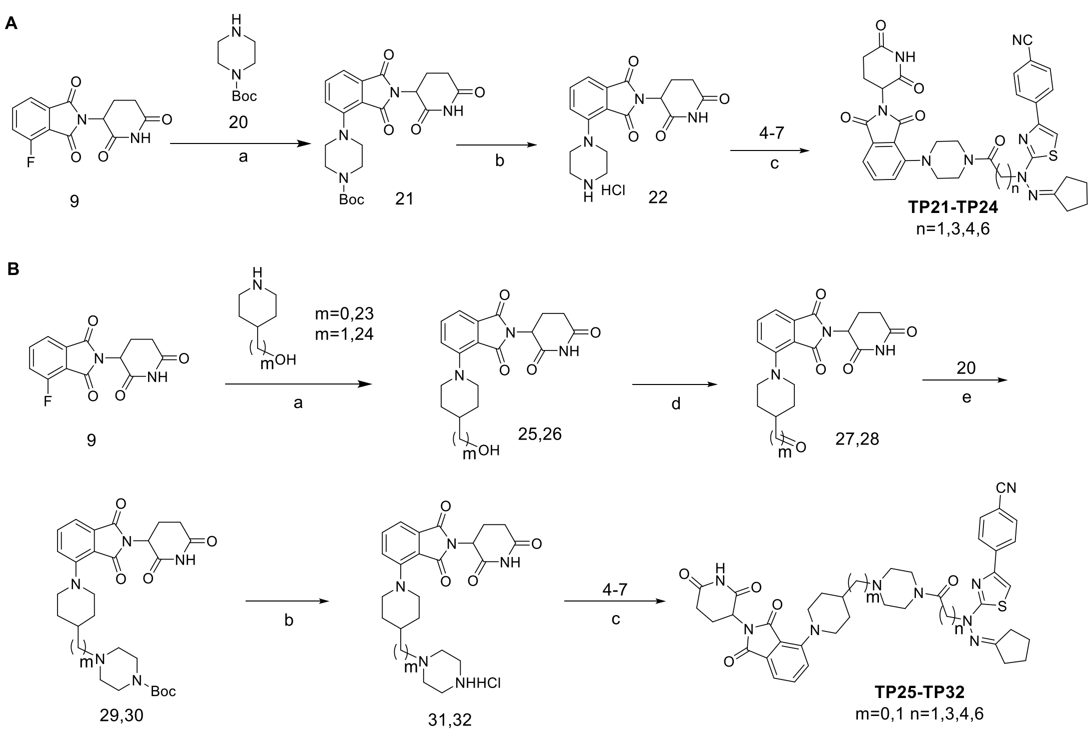

Reagents and conditions: (a) DIPEA, DMF, 90 °C, 4 h, 52.6-71.8 %; (b) 4M HCl-dioxane solution, DCM, rt, 2 h, 62.1%-76.9%; (c) HATU, TEA, DMF, rt, 4 h, 31.3-49.7 %; (d) Dess-Martin periodinane, DCM, rt, 2 h, 50-80 %; (e) $NaBH_3CN$, TEA, CH_3COOH, DCM/MeOH (1/1), rt, 3 h, 52-55 %

2-Cyclopentylidenehydrazine-1-carbothioamide (2)

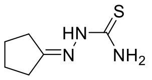

Thiosemicarbazide (1 g, 10.97 mmol) and cyclopentanone (0.92 g, 10.97 mmol) were dissolved in 30 mL of isopropanol, and the mixture was refluxed for 12 h. After the reaction was complete, the precipitate was filtered to produce intermediate 2 (1.44 g, 75% yield). White solid.

4-(2-(2-Cyclopentylidenehydrazineyl)thiazol-4-yl)benzonitrile (3)

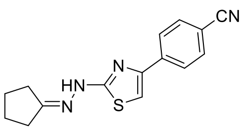

Intermediate 2 (1 g, 6.36 mmol) and 4-(2-bromoacetyl)benzonitrile (1.42 g, 6.36 mmol) were dissolved in 30 mL of isopropanol, and the mixture was stirred at room temperature for 12 h. After the reaction was complete, the precipitate was filtered to yield intermediate 3 (1.09 g, 45% yield). White solid. ^{1}H NMR (400 MHz, Chloroform-d) δ 12.13 (s, 1H), 7.91–7.84 (m, 2H), 7.80 (dd, J = 8.4, 2.1 Hz, 2H), 6.91 (s, 1H), 2.66 (t, J = 7.4 Hz, 2H), 2.56 (t, J = 7.4 Hz, 2H), 1.96 (q, J = 6.9 Hz, 2H), 1.89 (q, J = 7.0 Hz, 2H).

7-(1-(4-(4-Cyanophenyl)thiazol-2-yl)-2-cyclopentylidenehydrazineyl)heptanoic acid (7)

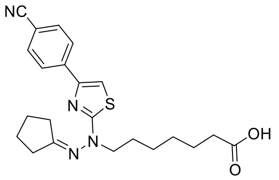

Intermediate 3 (1 g, 3.54 mmol), methyl 7-bromoheptanoate (1.19 g, 5.31 mmol) and potassium carbonate (1.47 g, 10.62 mmol) were dissolved in 5 mL of DMF, and the mixture was stirred at room temperature for 12 h. After the reaction was complete, the mixture was extracted with ethyl acetate. The organic layer was collected, evaporated under reduced pressure, and purified by column chromatography with petroleum ether/ethyl acetate (10:1) to give a white oil intermediate. The intermediate was dissolved in 36 mL of methanol, and a sodium hydroxide solution (10.0 equiv., dissolved in 4 mL of H_2O) was added. The reaction mixture was stirred at room temperature for 1 h. After the reaction was complete, the solvent was concentrated in vacuo, and then water and 3 M HCl were added to adjust the pH of the solution to 4-6. The residue was extracted with ethyl acetate and evaporated under reduced pressure to obtain compound 7 as a white oil (65.0% yield).

General procedure for the synthesis of compounds **4, 5** and **6**

These compounds were prepared in a similar manner as compound 7 but using appropriate esters to replace 7-bromoheptanoate to obtain a white oil (67.1-80.4% yield).

2-(2,6-Dioxopiperidin-3-yl)-4-fluoroisoindoline-1,3-dione (9)

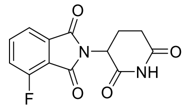

3-Fluorophthalic anhydride (5 g, 30.1 mmol), 3-aminopiperidine-2,6-dione hydrochloride (4.96 g, 30.1 mmol) and potassium acetate (8.87 g, 90.31 mmol) were dissolved in 50 mL of acetic acid, and the mixture was refluxed for 5 h. After the reaction was complete, the mixture was poured into ice water. The precipitate was filtered to yield intermediate 9 (6 g, 60.3% yield). Gray solid. ^{1}H NMR (400 MHz, DMSO-d6) δ 11.15 (s, 1H), 7.96 (td, J = 7.9, 4.4 Hz, 1H), 7.83–7.70 (m, 2H), 5.17 (dd, J = 12.9, 5.4 Hz, 1H), 2.90 (ddd, J = 17.2, 13.8, 5.4 Hz, 1H), 2.67–2.52 (m, 2H), 2.07 (ddt, J = 12.9, 5.6, 2.9 Hz, 1H).

4-((3-Aminopropyl)amino)-2-(2,6-dioxopiperidin-3-yl)isoindoline-1,3-dione hydrochloride (16)

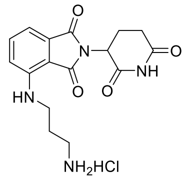

Intermediate 9 (1 g, 3.62 mmol), tert-butyl (3-aminopropyl)carbamate (0.76 g, 4.34 mmol) and DIPEA (935.81 mg, 7.24 mmol) were dissolved in 5 mL of DMF, and the mixture was stirred at 90 °C for 4 h. After the reaction was complete, the mixture was diluted with water and extracted with ethyl acetate. The organic layer was washed

with brine, dried over anhydrous Na_2SO_4, and concentrated in vacuo to provide the crude product, which was purified by column chromatography with petroleum ether/ethyl acetate (2:1) to produce the yellow solid intermediate 11. The intermediate (1.0 mmol) was dissolved in dichloromethane, and 4 M HCl-dioxane (3.0 mmol) was added with stirring at 0 °C. The resulting mixture was stirred for 2 h. After the reaction was complete, the precipitate was collected by filtration and dried to obtain yellow–green solid compound 16 (68.4% yield). ^{1}H NMR (400 MHz, DMSO-d6) δ 11.10 (s, 1H), 7.97 (s, 2H), 7.61 (t, *J* = 7.8 Hz, 1H), 7.17 (d, *J* = 8.6 Hz, 1H), 7.06 (d, *J* = 7.0 Hz, 1H), 6.76 (s, 1H), 5.06 (dd, *J* = 12.8, 5.4 Hz, 1H), 3.43 (t, *J* = 6.8 Hz, 2H), 2.87 (hept, *J* = 6.1, 5.2 Hz, 3H), 2.58 (td, *J* = 15.0, 14.4, 4.1 Hz, 2H), 2.05 (ddt, *J* = 12.8, 7.4, 4.4 Hz, 1H), 1.86 (p, *J* = 6.9 Hz, 2H).

tert-Butyl 4-(2-(2,6-dioxopiperidin-3-yl)-1,3-dioxoisoindolin-4-yl)piperazine-1-carboxylate (21)

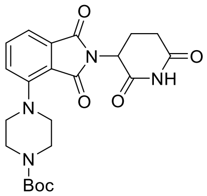

Intermediate 9 (1 g, 3.62 mmol), tert-butyl piperazine-1-carboxylate (809.15 mg, 4.34 mmol) and DIPEA (935.81 mg, 7.24 mmol) were dissolved in 5 mL of DMF, and the mixture was stirred at 90 °C for 4 h. After the reaction was complete, the mixture was diluted with water and extracted with ethyl acetate. The organic layer was washed with brine, dried over anhydrous Na_2SO_4, and concentrated in vacuo to provide intermediate 21 (1.3 g, 71.8% yield). Yellow solid.

2-(2,6-Dioxopiperidin-3-yl)-4-(piperazin-1-yl)isoindoline-1,3-dione hydrochloride (22)

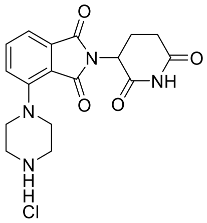

Intermediate 21 (1.0 mmol) was dissolved in dichloromethane, and 4 M HCl-dioxane (3.0 mmol) was added with stirring at 0 °C. The resulting mixture was stirred for 2 h. After the reaction was complete, the solvent was concentrated in vacuo to provide intermediate 22 **(**76.9% yield). Yellow solid. ^{1}H NMR (400 MHz, DMSO-*d*$_6$) δ 11.10 (s, 1H), 7.76 (t, *J* = 7.8 Hz, 1H), 7.47–7.39 (m, 2H), 5.11 (dd, *J* = 12.7, 5.4 Hz, 1H), 3.57 (s, 4H), 3.26 (s, 4H), 2.92–2.85 (m, 1H), 2.64–2.53 (m, 2H), 2.07–2.00 (m, 1H).

General procedure for the synthesis of compounds **25** and **26**

These compounds were prepared in a similar manner as compound 21 but piperidin-4-ol and piperidin-4-ylmethanol were used to replace tert-butyl piperazine-1-carboxylate to obtain a yellow solid (52.6-58.7% yield).

2-(2,6-Dioxopiperidin-3-yl)-4-(4-oxopiperidin-1-yl)isoindoline-1,3-dione (27)

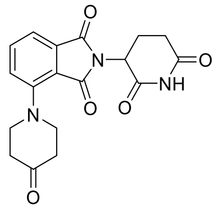

Intermediate 25 (1 g, 2.80 mmol) was dissolved in 20 mL of dichloromethane, and Dess–Martin periodinane (5.60 mmol) was added with stirring at 0 °C. After the reaction was complete, aqueous solutions of saturated sodium bicarbonate and sodium thiosulfate were added, and the mixture was extracted with dichloromethane. The organic layer was washed with brine, dried over anhydrous Na_2SO_4, and concentrated in vacuo to afford the crude product, which was purified by column chromatography with petroleum ether/ethyl acetate (1:1) to produce intermediate 27. Yellow solid (51% yield).

tert-Butyl 4-(1-(2-(2,6-dioxopiperidin-3-yl)-1,3-dioxoisoindolin-4-yl)piperidin-4-yl)piperazine-1-carboxylate (29)

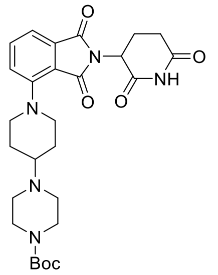

Intermediate 27 (1 g, 2.81 mmol) and tert-butyl piperazine-1-carboxylate (2.10 g, 11.26 mmol) were dissolved in dichloromethane, and 2 drops of acetic acid and $NaBH_3CN$ (884.2 mg, 14.07 mmol) were added. The mixture was stirred at room temperature for 3 h. The reaction mixture was extracted with dichloromethane. The organic layer was washed with brine, dried over anhydrous Na_2SO_4, and concentrated in vacuo to afford the crude product, which was purified by column chromatography with dichloromethane/methanol (50:1) to yield intermediate 29. Yellow solid (52% yield).

2-(2,6-Dioxopiperidin-3-yl)-4-(4-(piperazin-1-yl)piperidin-1-yl)isoindoline-1,3-dione hydrochloride (31)

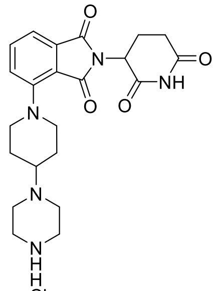

Intermediate 31 was prepared in a similar manner as compound 22 but intermediate 29 was used to replace intermediate 21 to obtain a yellow solid (68.7% yield).

2-(2,6-Dioxopiperidin-3-yl)-4-(4-(piperazin-1-ylmethyl)piperidin-1-yl)isoindoline-1,3-dione hydrochloride (31)

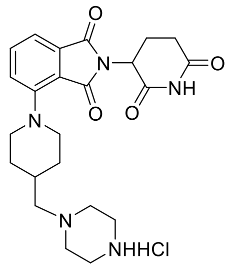

Intermediate 32 was prepared in a similar manner as intermediate 31 but intermediate 26 was used to replace intermediate 25 to obtain a yellow solid (62.1% yield).

5-(1-(4-(4-Cyanophenyl)thiazol-2-yl)-2-cyclopentylidenehydrazineyl)-N-(3-((2-(2,6-dioxopiperidin-3-yl)-1,3-dioxoisoindolin-4-yl)amino)propyl)pentanamide (TP12, NP1149)

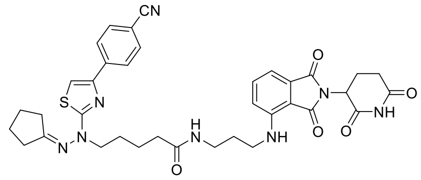

Intermediates 6 (40.0 mg, 0.10 mmol) and 16 (42.2 mg, 0.12 mmol) were dissolved in DMF, and triethylamine (42.3 mg, 0.42 mmol), EDCI (22.1 mg, 0.12 mmol) and HOBT (15.5 mg, 0.12 mmol) were added to the reaction solution. The mixture was stirred at room temperature for 4 h. After the reaction was complete, the mixture was extracted with ethyl acetate. The organic layer was concentrated in vacuo, and the residue was purified by column chromatography with dichloromethane/methanol (50:1) to yield compound NP1149. Yellow solid (57% yield). ^{1}H NMR (400 MHz, Chloroform-*d*) δ 8.72 (s, 1H), 7.93 (d, *J* = 8.0 Hz, 2H), 7.64 (d, *J* = 8.0 Hz, 2H), 7.47 (t, *J* = 7.8 Hz, 1H), 7.08 (d, *J* = 7.0 Hz, 1H), 6.98 (s, 1H), 6.82 (d, *J* = 8.5 Hz, 1H), 6.35 (t, *J* = 5.9 Hz, 1H), 5.93 (t, *J* = 6.1 Hz, 1H), 4.93 (dd, *J* = 12.1, 5.3 Hz, 1H), 3.93 (t, *J* = 6.7 Hz, 2H), 3.25 (dq, *J* = 13.0, 6.4 Hz, 4H), 2.89–2.80 (m, 1H), 2.80–2.69 (m, 2H), 2.63–2.55 (m, 2H), 2.51–2.42 (m, 2H), 2.27–2.21 (m, 2H), 2.14–2.07 (m, 1H), 1.93 (s, 1H), 1.86 (d, *J* = 5.5 Hz, 4H), 1.76–1.67 (m, 5H). ^{13}C NMR (101 MHz, Chloroform-*d*) δ 183.4, 173.1, 172.3, 171.4, 169.4, 168.6, 167.5, 149.7, 146.5, 139.1, 136.1, 132.4, 126.2, 116.5, 111.5, 110.4, 110.0, 106.8, 52.1, 48.8, 40.0, 36.9, 36.0, 33.8, 31.5, 31.4, 29.2, 26.0, 24.9, 24.2, 23.1, 22.7. RT = 15.52 min.

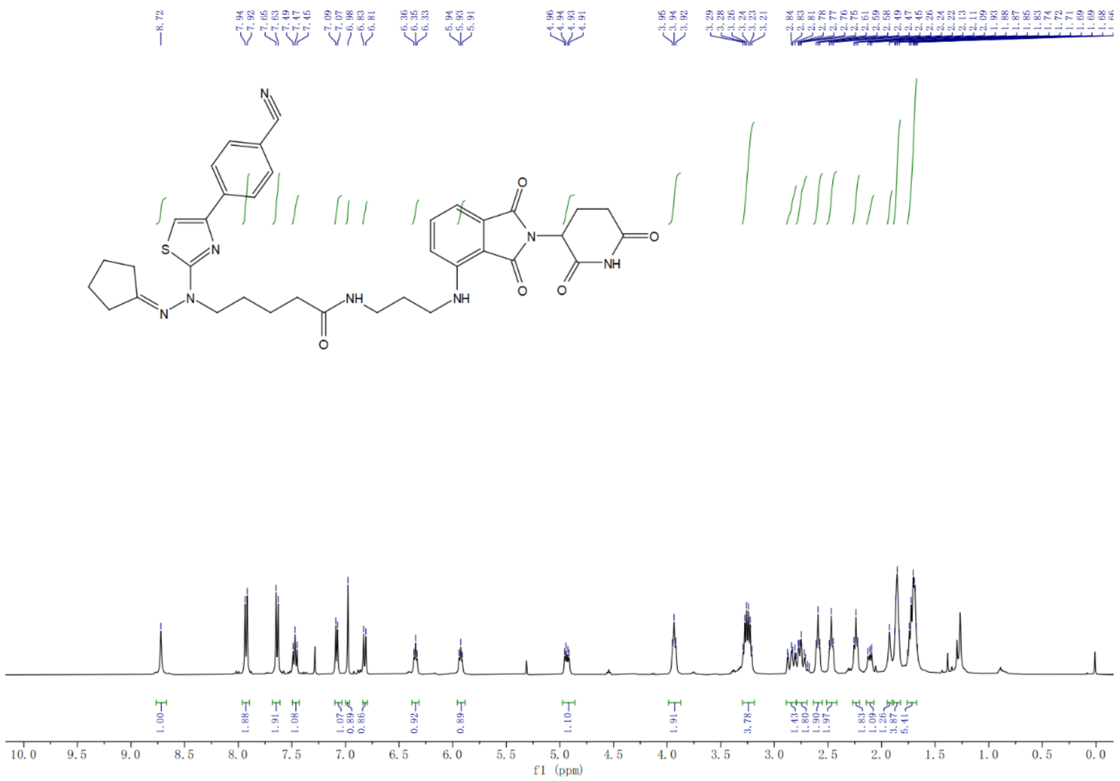

^{1}H NMR spectrum of compound NP1149.

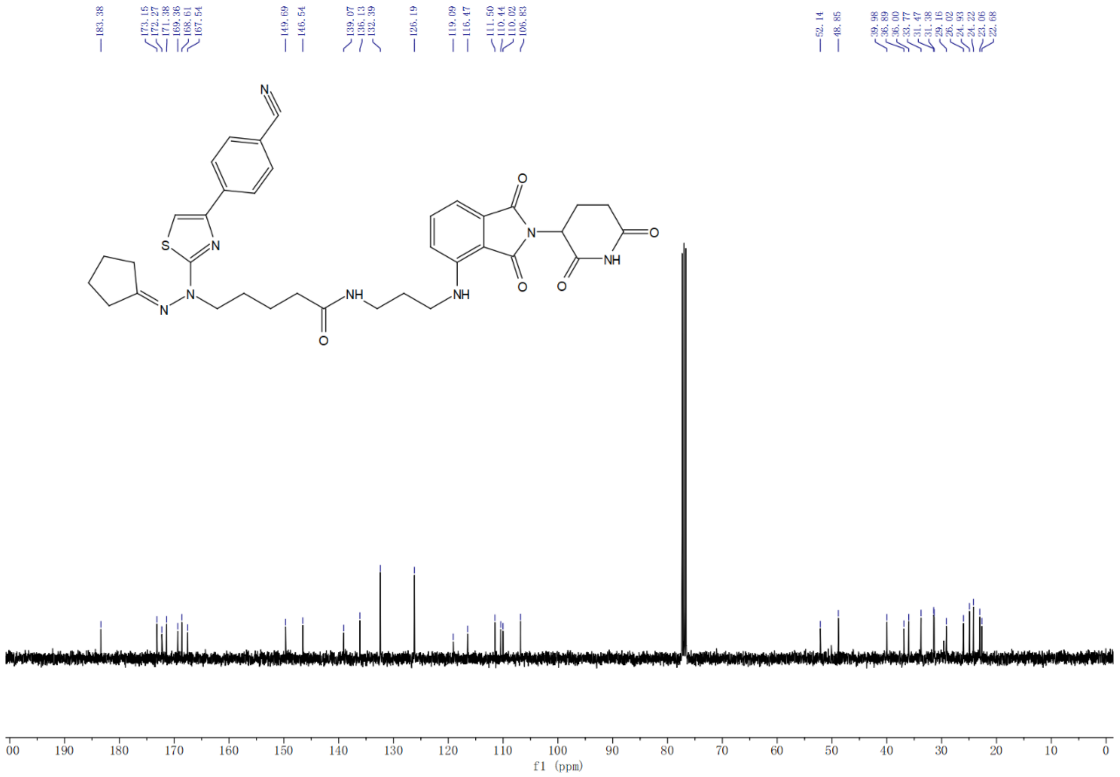

^{13}C NMR spectrum of compound NP1149.

4-(2-(2-Cyclopentylidene-1-(7-(4-(2-(2,6-dioxopiperidin-3-yl)-1,3-dioxoisoindolin-4-yl)piperazin-1-yl)-7-oxoheptyl)hydrazineyl)thiazol-4-yl)benzonitrile (TP24, NP1192)

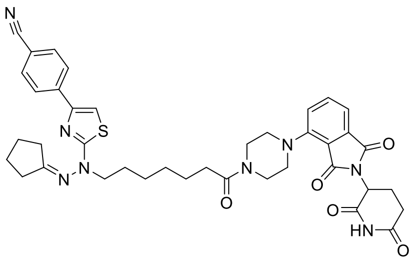

Intermediates 7 (0.5 g, 1.22 mmol) and 22 (0.51 g, 1.34 mmol) were dissolved in DMF, and triethylamine (0.49 g, 4.87 mmol) and HATU (0.56 g, 1.46 mmol) were added to the reaction solution. The mixture was stirred at room temperature for 4 h. After the reaction was complete, the mixture was extracted with ethyl acetate. The organic layer was concentrated in vacuo, and the residue was purified by column chromatography with dichloromethane/methanol (50:1) to yield compound NP1192 (35.3% yield). Yellow solid. ^{1}H NMR (400 MHz, Chloroform-*d*) δ 8.37 (s, 1H), 7.92 (d, *J* = 8.2 Hz, 2H), 7.67–7.62 (m, 3H), 7.46 (d, *J* = 7.2 Hz, 1H), 7.15 (d, *J* = 8.3 Hz, 1H), 6.94 (s, 1H), 4.99 (dd, *J* = 12.1, 5.4 Hz, 1H), 4.20 (s, 2H), 3.95–3.80 (m, 2H), 3.74 (dd, *J* = 9.4, 5.0 Hz, 2H), 3.67 (q, *J* = 7.4, 6.3 Hz, 2H), 3.27 (t, *J* = 5.1 Hz, 2H), 2.95–2.74 (m, 3H), 2.38 (t, *J* = 7.5 Hz, 2H), 2.14 (dd, *J* = 11.7, 5.5 Hz, 1H), 1.87 (p, *J* = 4.6 Hz, 2H), 1.81–1.74 (m, 4H), 1.69 (s, 4H), 1.46 (qt, *J* = 9.1, 4.5 Hz, 6H). ^{13}C NMR (101 MHz, Chloroform-*d*) δ 174.6, 171.7, 170.9, 168.2, 167.1, 166.6, 149.8, 139.3, 135.8, 134.1, 132.3, 126.2, 123.3, 119.2, 118.0, 116.4, 110.3, 106.2, 54.2, 51.9, 50.2, 49.2, 45.6, 41.3, 33.0, 31.4, 29.6, 29.0, 26.3, 25.8, 24.9, 22.6. RT = 15.03 min.

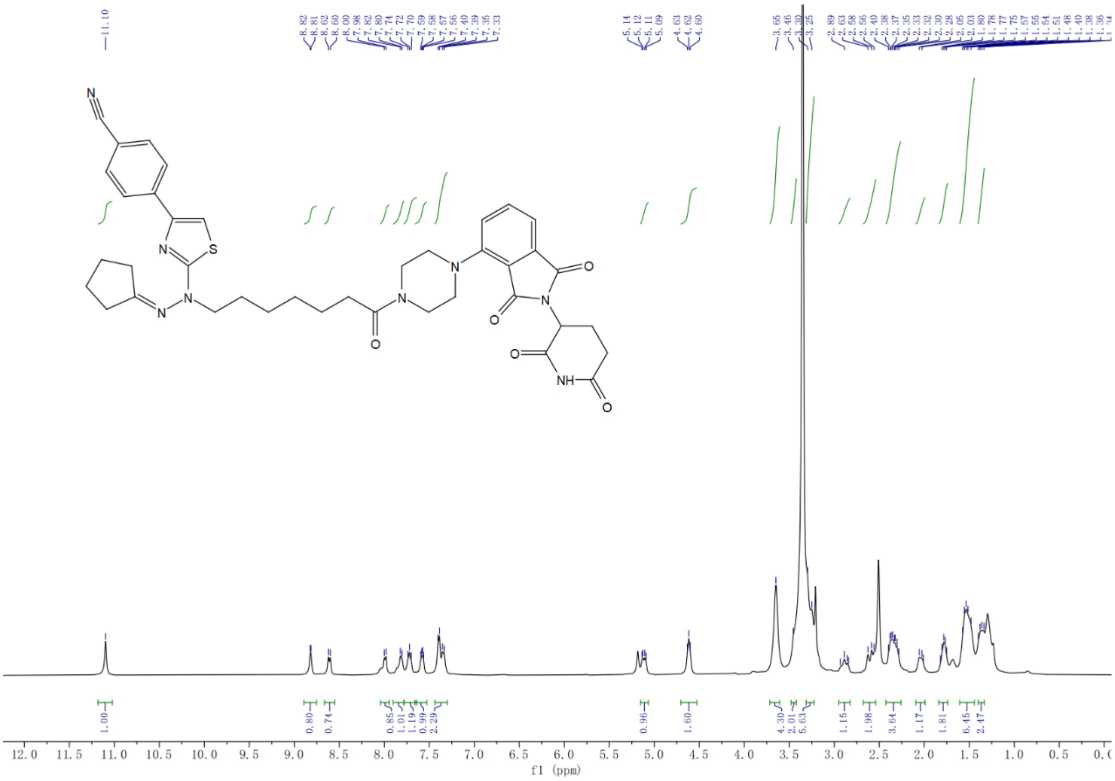

^{1}H NMR spectrum of compound NP1192.

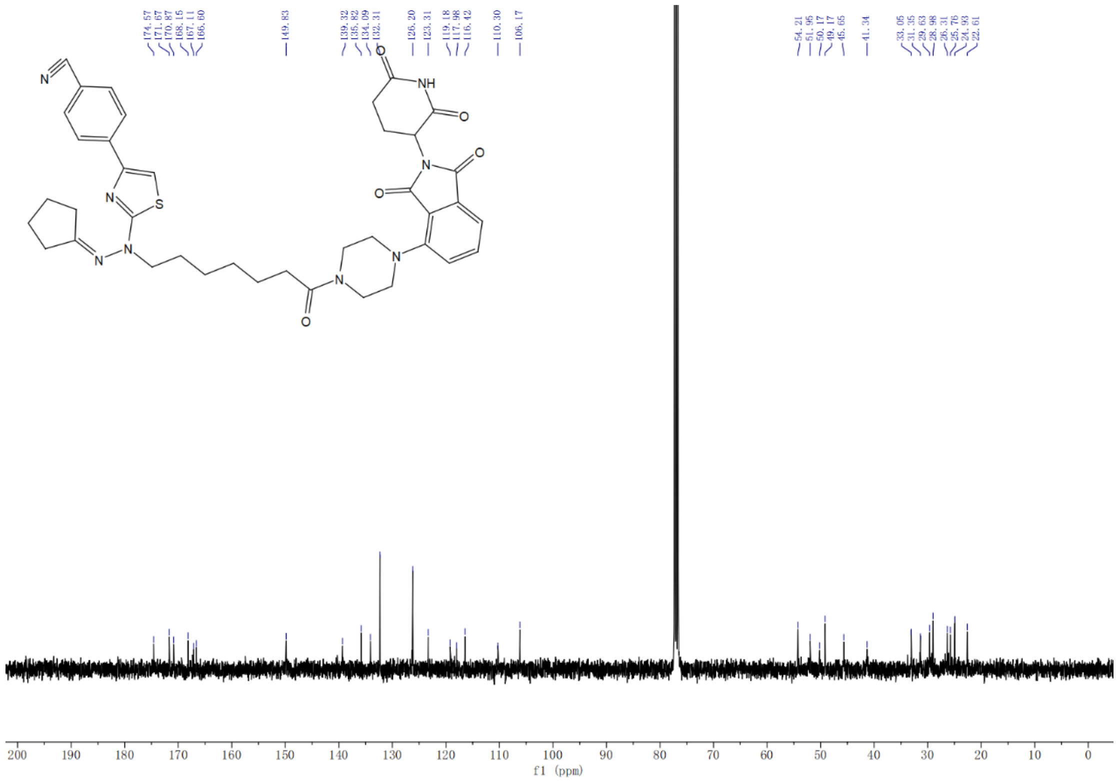
^{13}C NMR spectrum of NP1192.

General procedure for the synthesis of compounds **TP1–11** and **TP13–20**

These compounds were prepared in a similar manner as NP1149 but using the appropriate acid and amine to replace intermediates 6 and 16 to obtain a yellow solid (55.0-61.0% yield).

2-(1-(4-(4-Cyanophenyl)thiazol-2-yl)-2-cyclopentylidenehydrazineyl)-N-(2-((2-(2,6-dioxopiperidin-3-yl)-1,3-dioxoisoindolin-4-yl)amino)ethyl)acetamide (TP1)

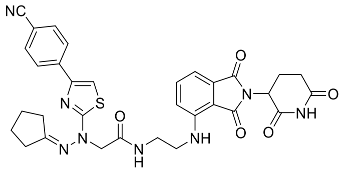

^{1}H NMR (400 MHz, Chloroform-*d*) δ 8.96 (s, 1H), 7.80 (d, *J* = 8.0 Hz, 2H), 7.54 (d, *J* = 8.0 Hz, 2H), 7.47 (d, *J* = 6.2 Hz, 1H), 7.33 (t, *J* = 8.0 Hz, 1H), 7.07 (s, 1H), 6.99 (d, *J* = 7.1 Hz, 1H), 6.78 (d, *J* = 8.5 Hz, 1H), 6.17 (d, *J* = 5.9 Hz, 1H), 4.96–4.85 (m, 1H), 4.52–4.34 (m, 2H), 3.57 (tq, *J* = 13.8, 7.2 Hz, 2H), 3.37 (h, *J* = 7.0, 6.3 Hz, 2H), 2.80 (dt, *J* = 24.0, 11.4 Hz, 3H), 2.63–2.44 (m, 4H), 2.08 (d, *J* = 5.7 Hz, 1H), 1.82 (d, *J* = 7.1 Hz, 4H). ^{13}C NMR (101 MHz, Chloroform-*d*) δ 183.4, 171.9, 171.7, 169.6, 169.1, 168.6, 167.4, 149.4, 146.5, 138.4, 136.0, 132.3, 126.0, 116.5, 111.8, 110.5, 110.0, 108.2, 57.5, 48.8, 42.2, 38.7, 33.9, 31.5, 31.4, 25.0, 24.2, 22.6. RT = 15.85 min.

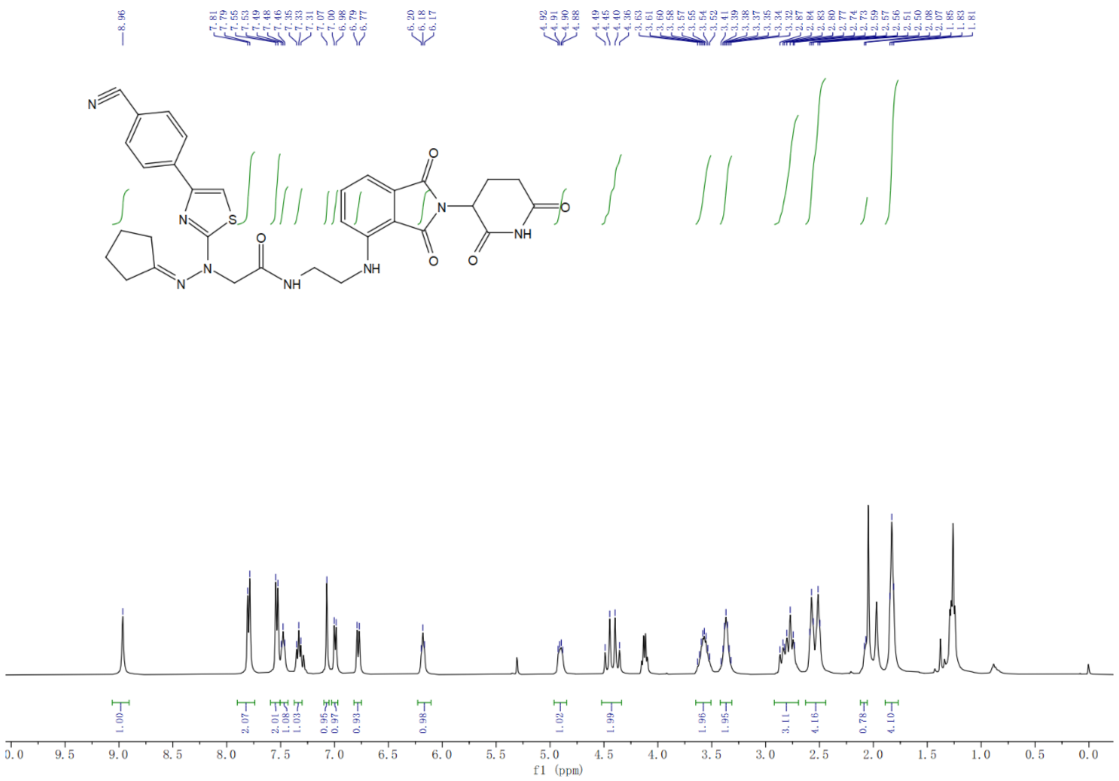

^{1}H NMR spectrum of compound TP1.

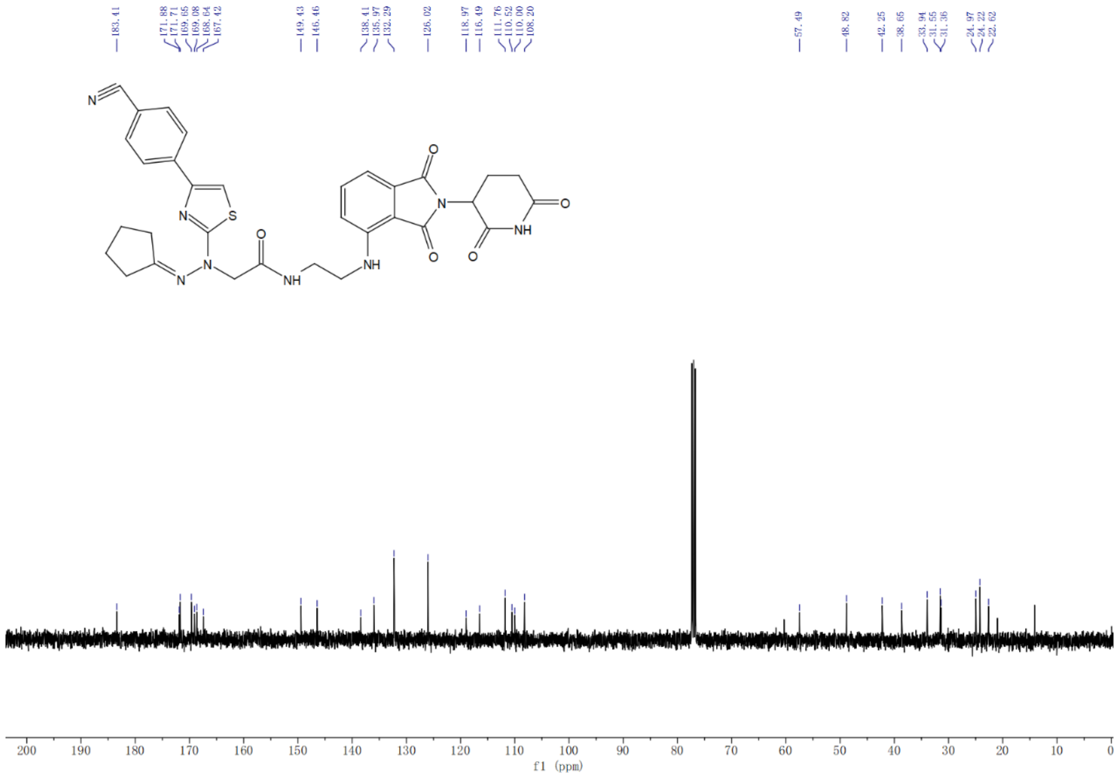

^{13}C NMR spectrum of compound TP1.

2-(1-(4-(4-Cyanophenyl)thiazol-2-yl)-2-cyclopentylidenehydrazineyl)-N-(3-((2-(2,6-dioxopiperidin-3-yl)-1,3-dioxoisoindolin-4-yl)amino)propyl)acetamide (TP2)

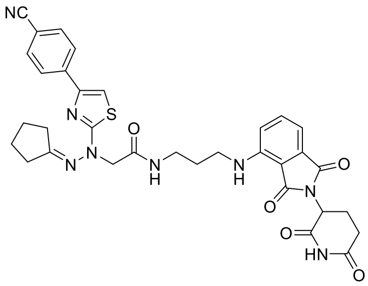

^{1}H NMR (400 MHz, Chloroform-*d*) δ 8.60 (s, 1H), 7.86 (d, *J* = 8.1 Hz, 2H), 7.60 (d, *J* = 8.7 Hz, 2H), 7.36 (t, *J* = 7.9 Hz, 1H), 7.15 (d, *J* = 6.4 Hz, 1H), 7.10–6.99 (m, 2H), 6.68 (d, *J* = 8.5 Hz, 1H), 6.19 (d, *J* = 6.1 Hz, 1H), 4.97–4.88 (m, 1H), 4.43 (s, 2H), 3.42 (q, *J* = 6.7 Hz, 2H), 3.15 (q, *J* = 6.8 Hz, 2H), 2.90–2.67 (m, 3H), 2.57 (dt, *J* = 20.1, 7.2 Hz, 4H), 2.17–2.09 (m, 1H), 1.90–1.83 (m, 4H), 1.78 (t, *J* = 7.0 Hz, 2H). ^{13}C NMR (101 MHz, Chloroform-d) δ 183.0, 172.4, 171.4, 171.1, 169.3, 168.6, 167.5, 149.7, 146.4, 138.4, 135.9, 132.4, 126.1, 118.9, 116.3, 111.4, 110.8, 110.0, 108.0, 57.8, 48.9, 39.7, 36.6, 34.1, 31.5, 31.4, 29.3, 25.0, 24.2, 22.7. RT = 14.83 min.

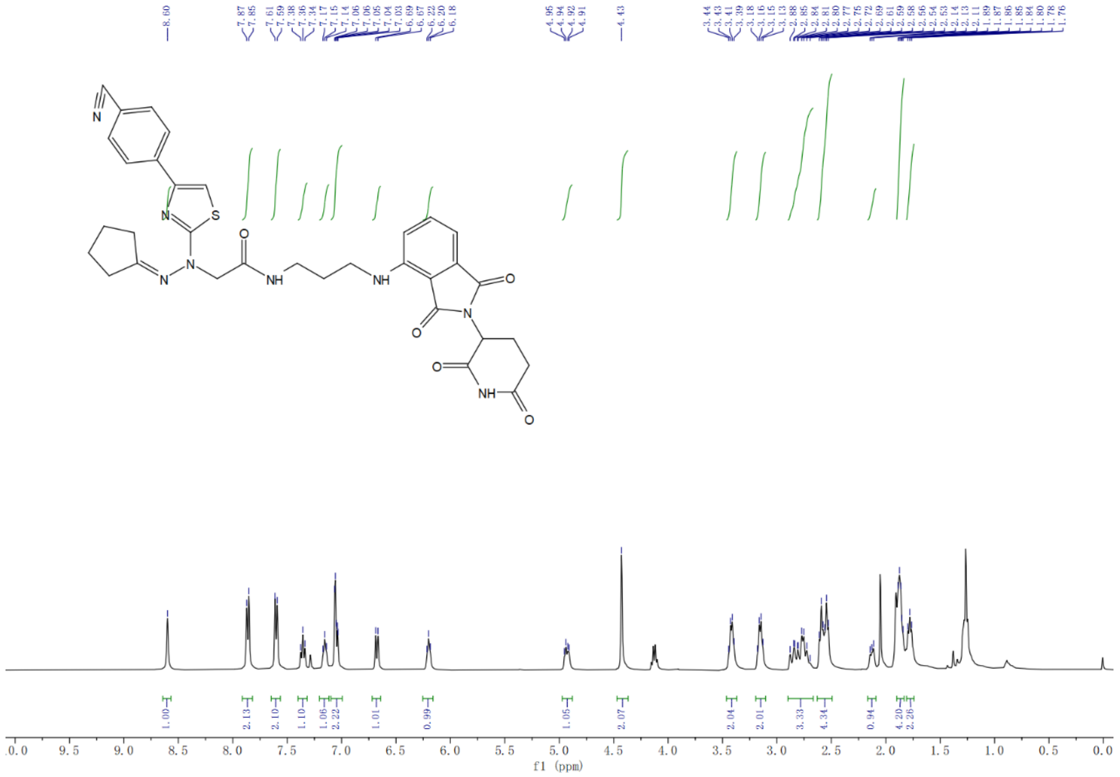

^{1}H NMR spectrum of compound TP2.

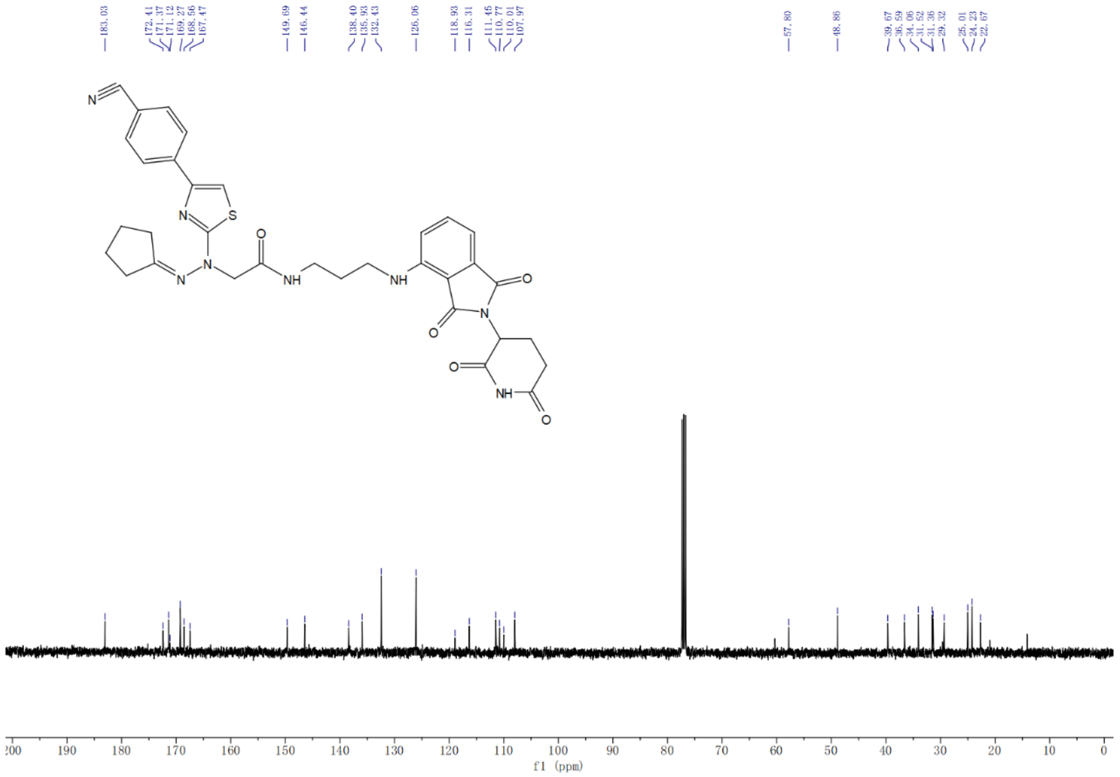

^{13}C NMR spectrum of compound TP2.

2-(1-(4-(4-Cyanophenyl)thiazol-2-yl)-2-cyclopentylidenehydrazineyl)-N-(4-((2-(2,6-dioxopiperidin-3-yl)-1,3-dioxoisoindolin-4-yl)amino)butyl)acetamide (TP3)

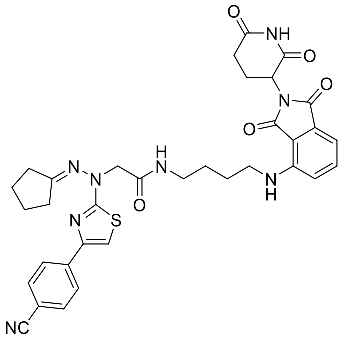

^{1}H NMR (400 MHz, Chloroform-*d*) δ 8.56 (s, 1H), 7.87 (d, *J* = 8.0 Hz, 2H), 7.57 (d, *J* = 8.0 Hz, 2H), 7.45 (t, *J* = 7.8 Hz, 1H), 7.08 (d, *J* = 3.1 Hz, 2H), 7.02 (d, *J* = 6.3 Hz, 1H), 6.70 (d, *J* = 8.5 Hz, 1H), 5.97 (d, *J* = 5.8 Hz, 1H), 4.95 (dd, *J* = 11.8, 5.3 Hz, 1H), 4.43 (d, *J* = 4.0 Hz, 2H), 3.34 (p, *J* = 6.4 Hz, 2H), 3.05 (q, *J* = 6.8 Hz, 2H), 2.92–2.71 (m, 3H), 2.57 (dt, *J* = 21.7, 7.3 Hz, 4H), 2.19–2.08 (m, 1H), 1.94–1.86 (m, 4H), 1.53 (dq, *J* = 15.6, 7.7 Hz, 4H). ^{13}C NMR (101 MHz, Chloroform-*d*) δ 183.1, 172.3, 171.3, 169.4, 169.0, 168.6, 167.5, 149.7, 146.5, 138.4, 136.1, 132.3, 132.3, 126.1, 116.4, 111.5, 110.7, 109.7, 108.1, 57.6, 48.9, 42.1, 38.7, 34.1, 31.5, 31.4, 26.8, 26.2, 25.0, 24.2, 22.7. RT = 16.90 min.

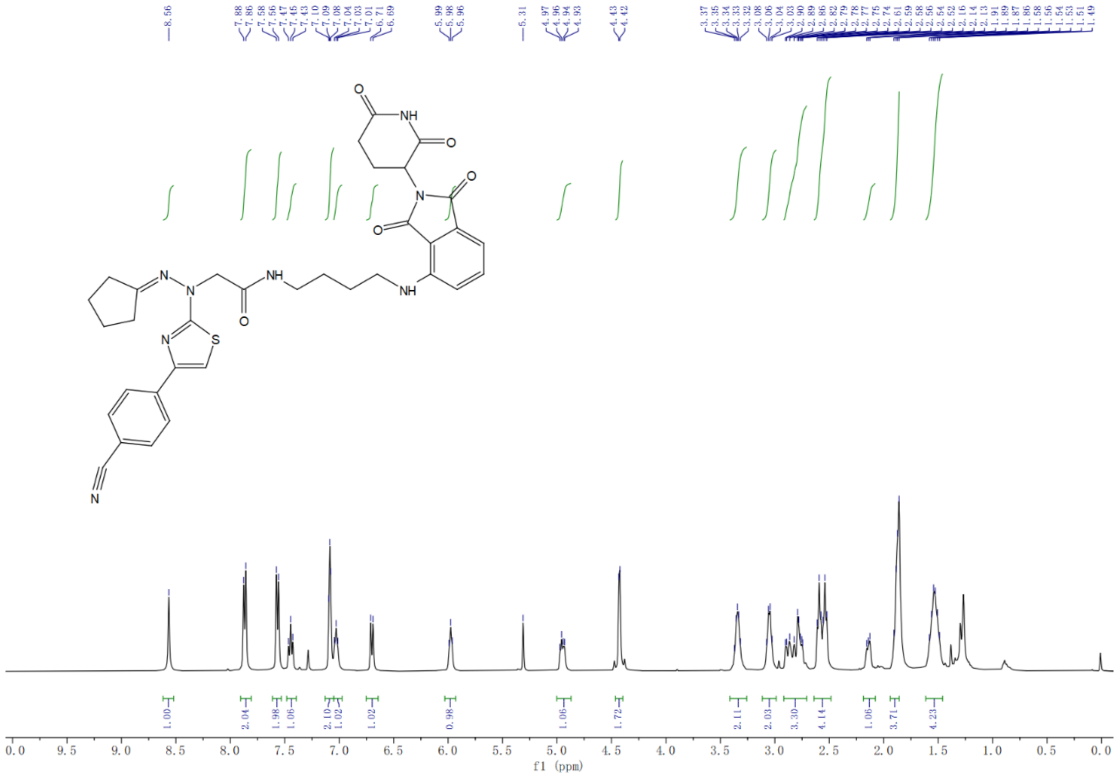

^{1}H NMR spectrum of compound TP3.

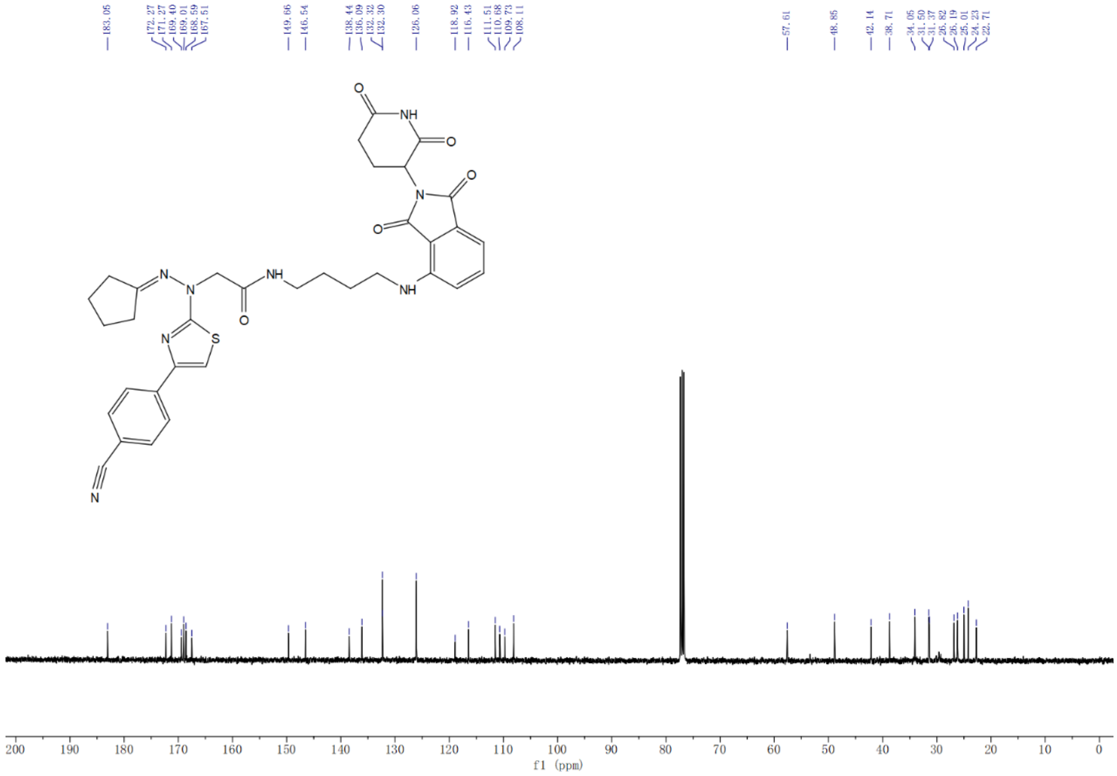

^{13}C NMR spectrum of compound TP3.

2-(1-(4-(4-Cyanophenyl)thiazol-2-yl)-2-cyclopentylidenehydrazineyl)-N-(5-((2-(2,6-dioxopiperidin-3-yl)-1,3-dioxoisoindolin-4-yl)amino)pentyl)acetamide (TP4)

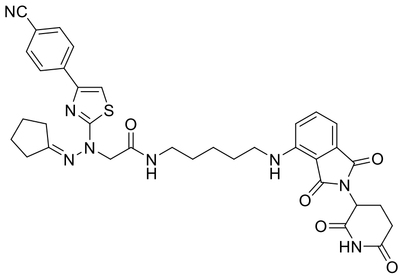

^{1}H NMR (400 MHz, Chloroform-*d*) δ 8.24 (s, 1H), 7.91 (d, *J* = 8.2 Hz, 2H), 7.64 (d, *J* = 8.7 Hz, 2H), 7.49 (t, *J* = 8.0 Hz, 1H), 7.15–7.06 (m, 2H), 6.98 (d, *J* = 6.1 Hz, 1H), 6.76 (d, *J* = 8.5 Hz, 1H), 6.09 (d, *J* = 5.9 Hz, 1H), 4.94 (dd, *J* = 12.1, 5.5 Hz, 1H), 4.43 (s, 2H), 3.32 (q, *J* = 6.7 Hz, 2H), 3.08 (q, *J* = 6.7 Hz, 2H), 2.94–2.73 (m, 3H), 2.58 (dt, *J* = 21.0, 7.3 Hz, 4H), 2.16 (dd, *J* = 13.2, 5.9 Hz, 1H), 1.94–1.83 (m, 4H), 1.69 (s, 2H), 1.52 (dq, *J* = 14.9, 7.5 Hz, 4H). ^{13}C NMR (101 MHz, Chloroform-*d*) δ 182.6, 172.5, 171.0, 169.4, 168.8, 168.3, 167.5, 149.7, 146.7, 138.5, 136.1, 132.4, 126.1, 116.4, 111.5, 110.9, 109.9, 107.9, 57.8, 48.8, 42.3, 39.0, 34.1, 31.5, 31.4, 29.2, 28.7, 25.0, 24.2, 22.7. RT = 15.55 min.

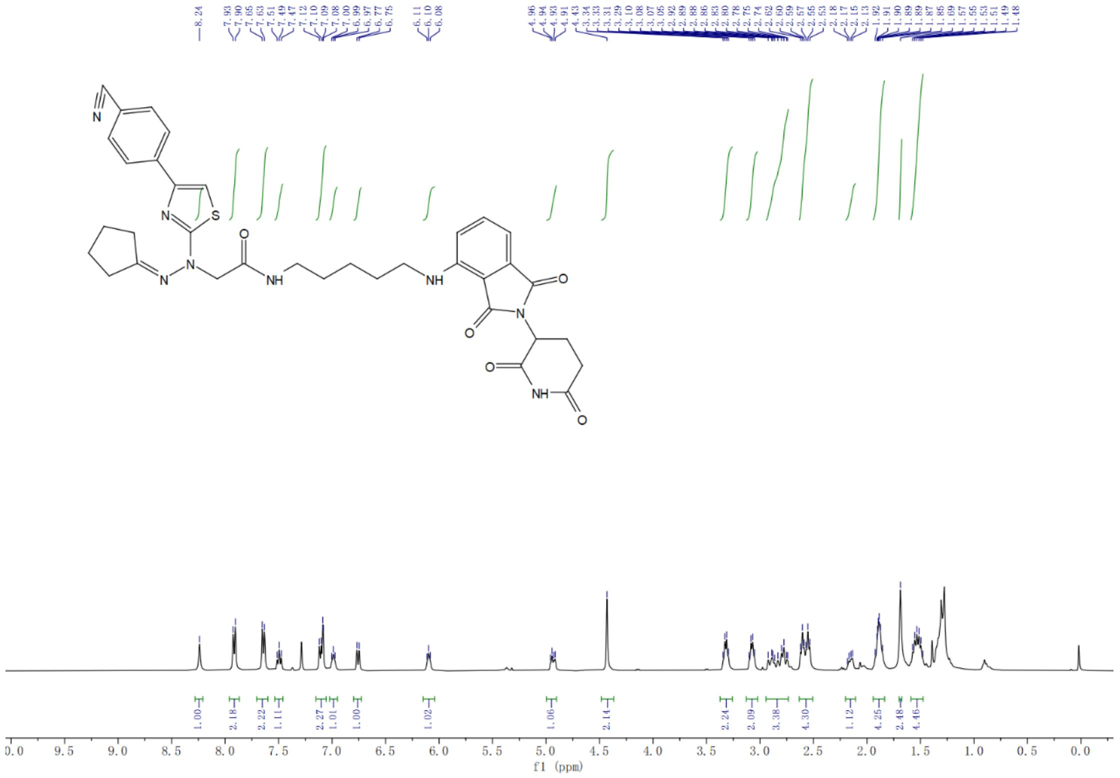

^{1}H NMR spectrum of compound TP4.

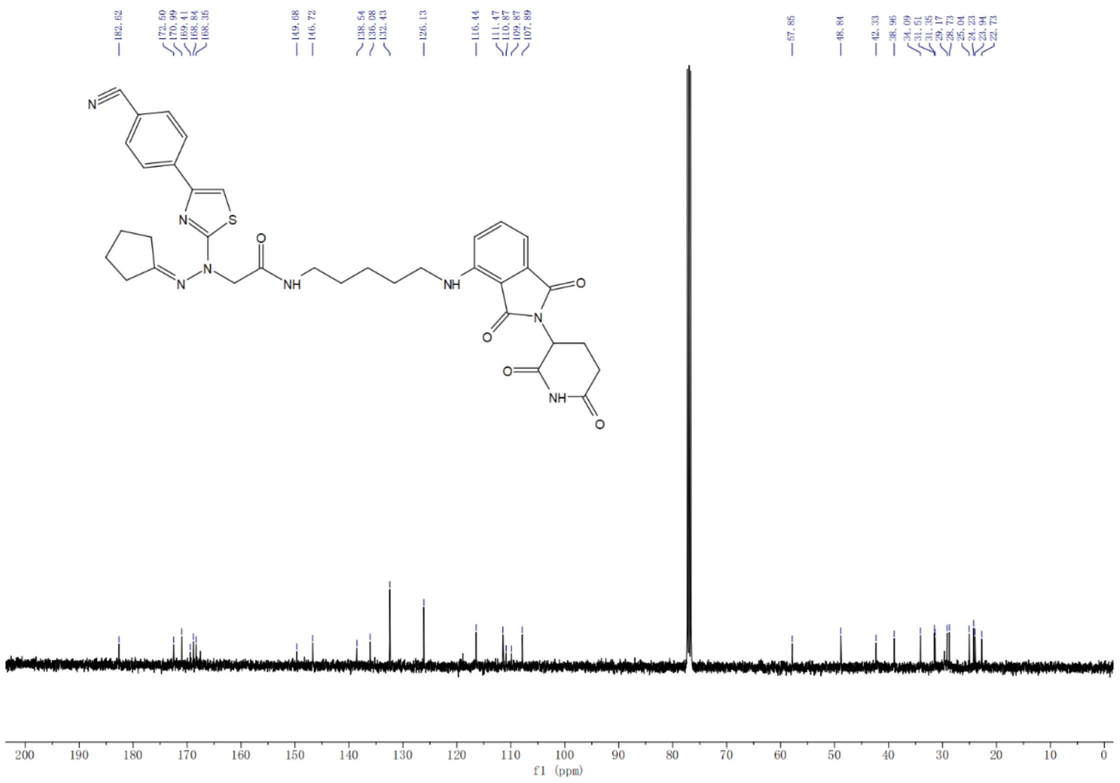

^{13}C NMR spectrum of compound TP4.

2-(1-(4-(4-Cyanophenyl)thiazol-2-yl)-2-cyclopentylidenehydrazineyl)-N-(6-((2-(2,6-dioxopiperidin-3-yl)-1,3-dioxoisoindolin-4-yl)amino)hexyl)acetamide (TP5)

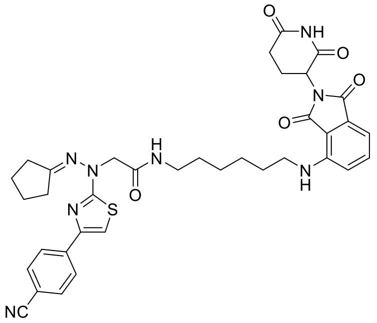

^1H NMR (400 MHz, Chloroform-*d*) δ 8.50 (s, 1H), 7.91 (d, *J* = 8.1 Hz, 2H), 7.64 (d, *J* = 8.0 Hz, 2H), 7.49 (t, *J* = 7.9 Hz, 1H), 7.09 (d, *J* = 7.5 Hz, 2H), 6.95 (d, *J* = 6.0 Hz, 1H), 6.81 (d, *J* = 8.6 Hz, 1H), 6.12 (d, *J* = 5.7 Hz, 1H), 4.94 (dd, *J* = 11.9, 5.5 Hz, 1H), 4.42 (s, 2H), 3.29 (q, *J* = 6.6 Hz, 2H), 3.12 (q, *J* = 6.7 Hz, 2H), 2.92–2.71 (m, 3H), 2.57 (dt, *J* = 20.7, 7.3 Hz, 4H), 2.20–2.09 (m, 1H), 1.92–1.82 (m, 6H), 1.47 (d, *J* = 7.4 Hz, 4H). ^{13}C NMR (101 MHz, Chloroform-*d*) δ 182.5, 172.5, 171.2, 169.5, 168.8, 168.5, 167.6, 149.7, 146.8, 138.6, 136.1, 132.4, 126.2, 116.5, 111.4, 110.8, 109.8, 107.9, 57.8, 48.8, 42.4, 39.1, 34.1, 31.5, 31.4, 29.3, 29.0, 26.4, 26.3, 25.0, 24.2, 22.7. RT = 18.25 min.

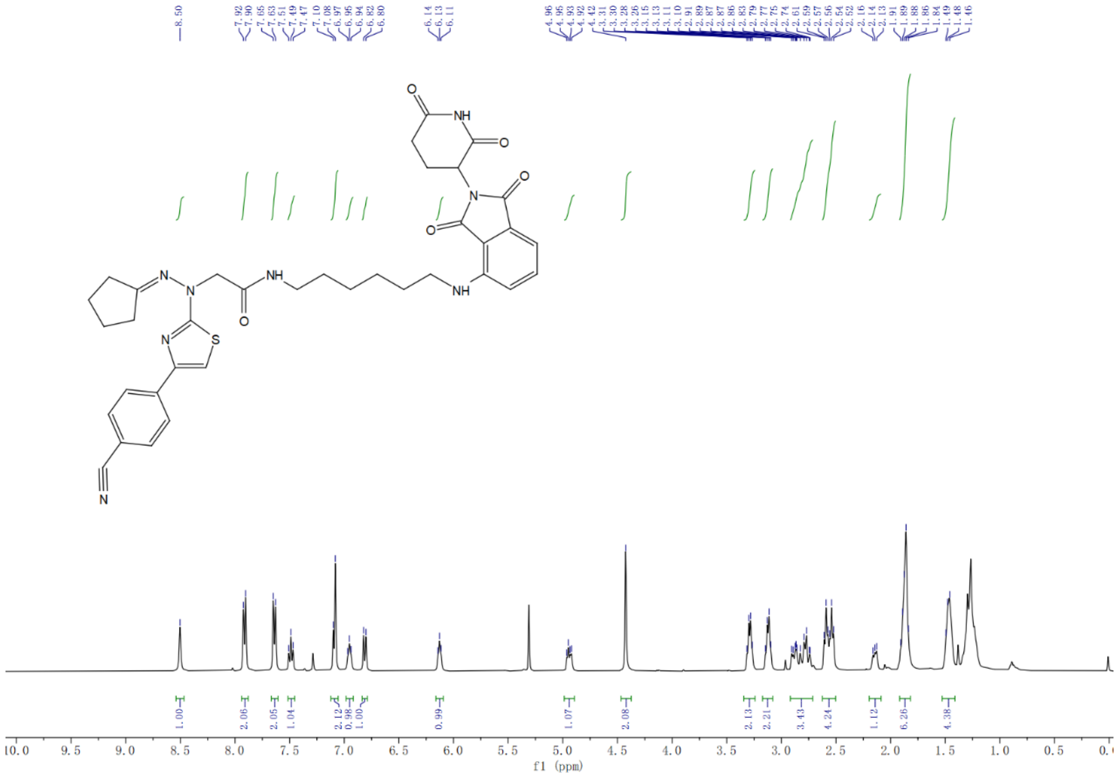

^{1}H NMR spectrum of compound TP5.

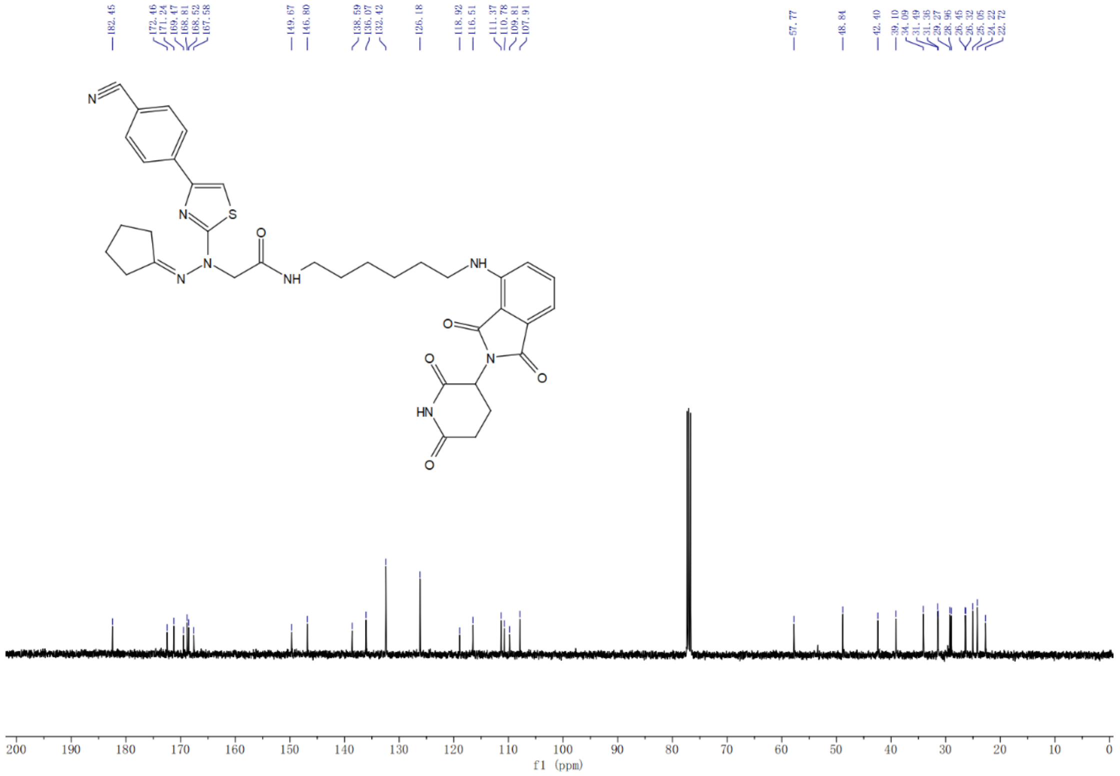

^{13}C NMR spectrum of compound TP5.

4-(1-(4-(4-Cyanophenyl)thiazol-2-yl)-2-cyclopentylidenehydrazineyl)-N-(2-((2-(2,6-dioxopiperidin-3-yl)-1,3-dioxoisoindolin-4-yl)amino)ethyl)butanamide (TP6)

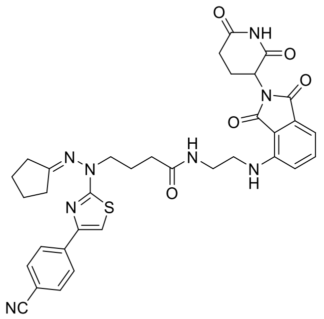

^{1}H NMR (400 MHz, Chloroform-*d*) δ 8.58 (s, 1H), 7.89 (d, *J* = 8.0 Hz, 2H), 7.63 (d, *J* = 8.0 Hz, 2H), 7.47 (t, *J* = 7.9 Hz, 1H), 7.09 (d, *J* = 7.2 Hz, 1H), 6.96 (d, *J* = 2.1 Hz, 1H), 6.91 (d, *J* = 8.6 Hz, 1H), 6.47 (d, *J* = 6.1 Hz, 1H), 6.35 (d, *J* = 5.4 Hz, 1H), 4.92 (dd, *J* = 12.1, 5.4 Hz, 1H), 3.98 (t, *J* = 7.1 Hz, 2H), 3.40 (s, 4H), 2.92–2.70 (m, 3H), 2.58 (d, *J* = 6.8 Hz, 2H), 2.47 (d, *J* = 7.2 Hz, 2H), 2.26 (t, *J* = 7.3 Hz, 2H), 2.16–2.08 (m, 1H), 2.01 (q, *J* = 7.4 Hz, 2H), 1.86 (d, *J* = 5.9 Hz, 2H), 1.80 (s, 2H). ^{13}C NMR (101 MHz, Chloroform-*d*) δ 183.3, 173.0, 172.5, 171.3, 169.3, 168.6, 167.4, 149.7, 146.6, 139.1, 136.2, 132.4, 126.3, 116.6, 111.9, 110.6, 110.2, 106.9, 52.0, 48.9, 42.0, 39.0, 33.8, 33.5, 31.5, 31.4, 24.9, 24.2, 22.7, 22.7. RT = 15.14 min.

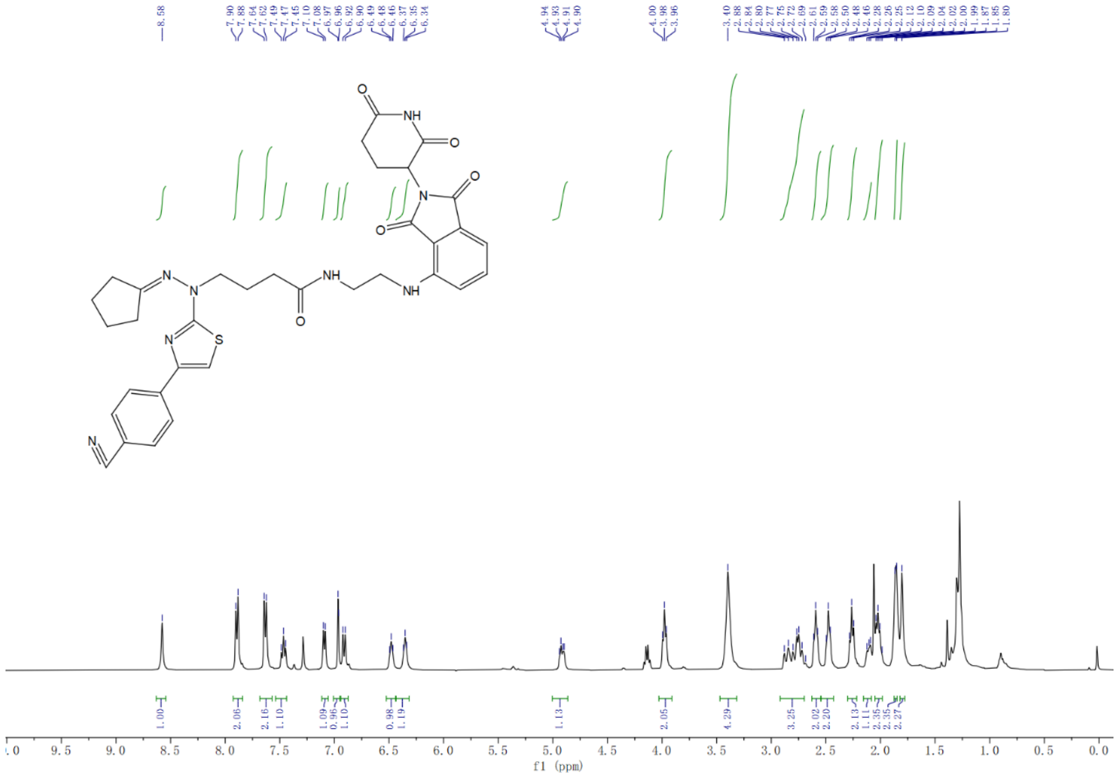

^{1}H NMR spectrum of compound TP6.

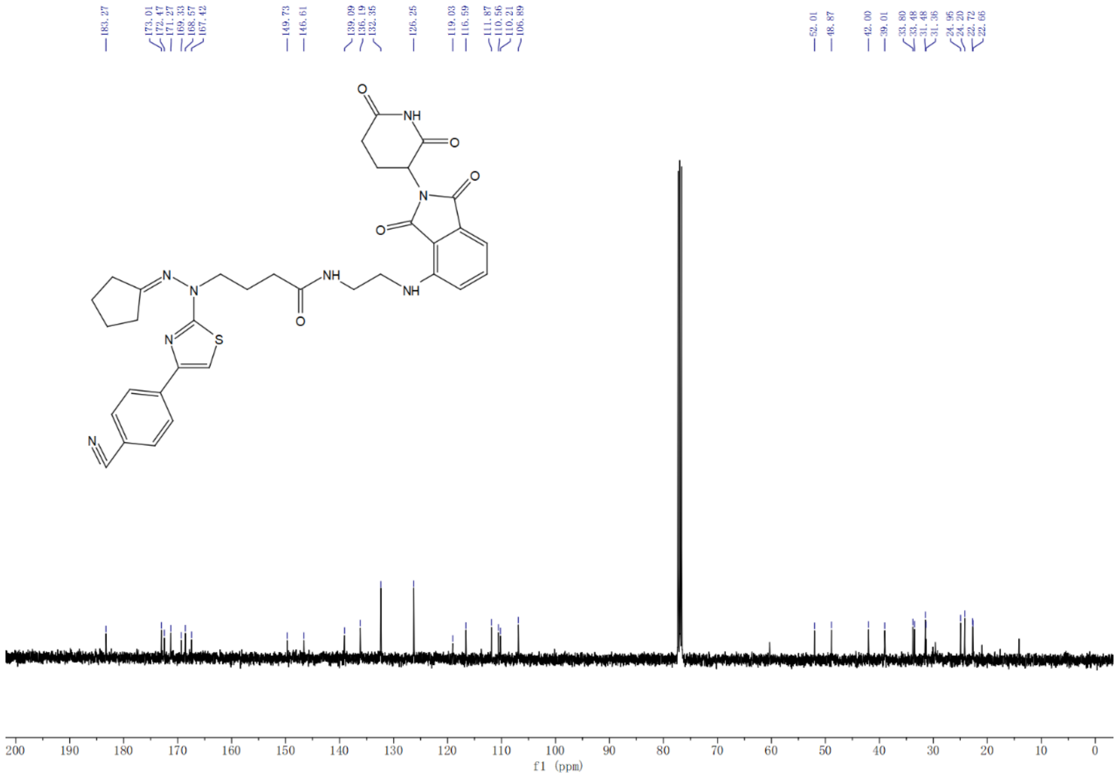

^{13}C NMR spectrum of compound TP6.

4-(1-(4-(4-Cyanophenyl)thiazol-2-yl)-2-cyclopentylidenehydrazineyl)-N-(3-((2-(2,6-dioxopiperidin-3-yl)-1,3-dioxoisoindolin-4-yl)amino)propyl)butanamide (TP7, NP1171)

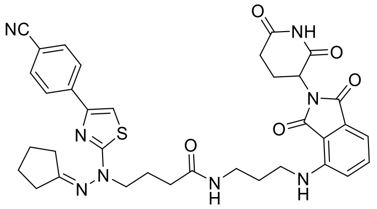

^{1}H NMR (400 MHz, Chloroform-*d*) δ 8.73 (s, 1H), 7.90 (d, *J* = 8.1 Hz, 2H), 7.63 (d, *J* = 8.1 Hz, 2H), 7.46 (t, *J* = 7.9 Hz, 1H), 7.07 (d, *J* = 7.3 Hz, 1H), 6.97 (s, 1H), 6.81 (d, *J* = 8.6 Hz, 1H), 6.34 (d, *J* = 6.1 Hz, 1H), 6.23 (t, *J* = 6.1 Hz, 1H), 5.02–4.88 (m, 1H), 3.98 (t, *J* = 7.0 Hz, 2H), 3.27 (dq, *J* = 25.8, 6.6 Hz, 4H), 2.92–2.68 (m, 3H), 2.59 (d, *J* = 7.0 Hz, 2H), 2.47 (d, *J* = 7.2 Hz, 2H), 2.26 (t, *J* = 7.3 Hz, 2H), 2.15–2.09 (m, 1H), 2.02 (t, *J* = 7.0 Hz, 2H), 1.86 (d, *J* = 5.7 Hz, 4H), 1.75 (p, *J* = 6.8 Hz, 2H). ^{13}C NMR (101 MHz, Chloroform-*d*) δ 183.5, 172.7, 172.4, 171.4, 168.6, 149.7, 146.5, 139.1, 136.1, 132.3, 126.3, 116.4, 111.5, 110.5, 110.0, 106.9, 52.0, 48.8, 40.0, 37.0, 33.8, 33.6, 31.5, 31.4, 29.2, 24.9, 24.2, 22.8, 22.7. RT = 15.37 min.

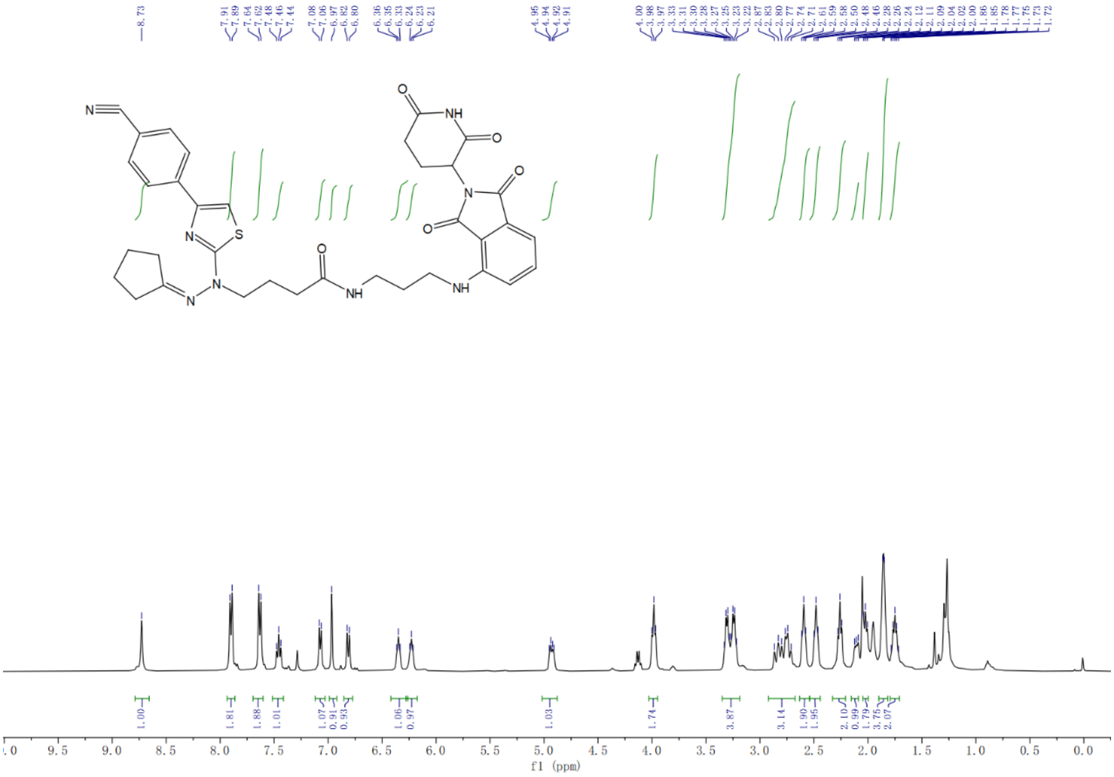

^{1}H NMR spectrum of compound NP1171.

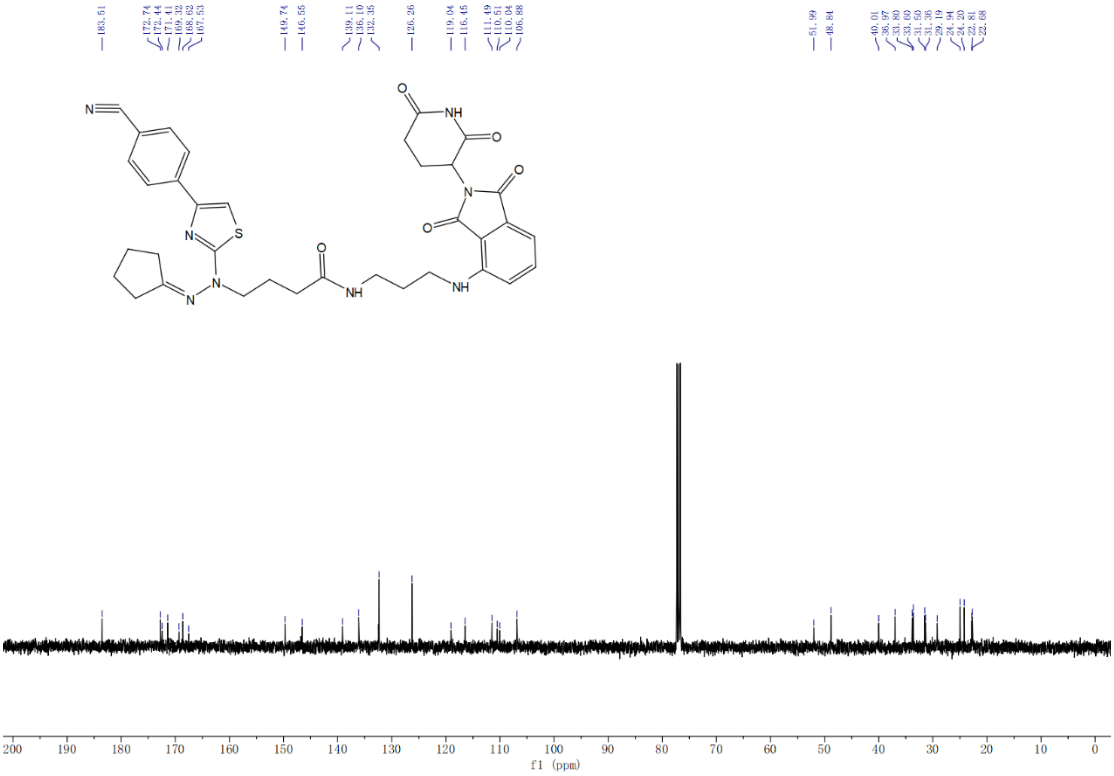

^{13}C NMR spectrum of compound NP1171.

4-(1-(4-(4-Cyanophenyl)thiazol-2-yl)-2-cyclopentylidenehydrazineyl)-N-(4-((2-(2,6-dioxopiperidin-3-yl)-1,3-dioxoisoindolin-4-yl)amino)butyl)butanamide (TP8)

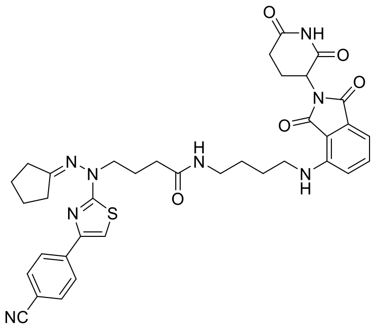

^{1}H NMR (400 MHz, Chloroform-*d*) δ 8.63 (s, 1H), 7.91 (d, *J* = 8.0 Hz, 2H), 7.64 (d, *J* = 8.0 Hz, 2H), 7.48 (t, *J* = 7.9 Hz, 1H), 7.08 (d, *J* = 7.1 Hz, 1H), 6.97 (s, 1H), 6.84 (d, *J* = 8.6 Hz, 1H), 6.18 (d, *J* = 6.0 Hz, 1H), 6.00 (d, *J* = 6.3 Hz, 1H), 4.93 (dd, *J* = 11.8, 5.3 Hz, 1H), 3.98 (t, *J* = 7.1 Hz, 2H), 3.23 (q, *J* = 6.7 Hz, 4H), 2.94–2.71 (m, 3H), 2.58 (d, *J* = 7.1 Hz, 2H), 2.47 (d, *J* = 7.3 Hz, 2H), 2.24 (t, *J* = 7.3 Hz, 2H), 2.16 – 2.08 (m, 1H), 2.04–1.98 (m, 2H), 1.86 (d, *J* = 5.4 Hz, 4H), 1.64–1.58 (m, 2H), 1.52 (t, *J* = 7.7 Hz, 2H). ^{13}C NMR (101 MHz, Chloroform-*d*) δ 183.2, 172.4, 172.3, 171.3, 169.5, 168.6, 167.5, 149.7, 146.7, 139.2, 136.1, 132.3, 126.3, 116.6, 111.5, 110.5, 109.9, 106.9, 52.0, 48.8, 42.1, 38.9, 33.8, 33.7, 31.5, 31.4, 27.0, 26.4, 24.9, 24.2, 22.8, 22.7. RT = 15.57 min.

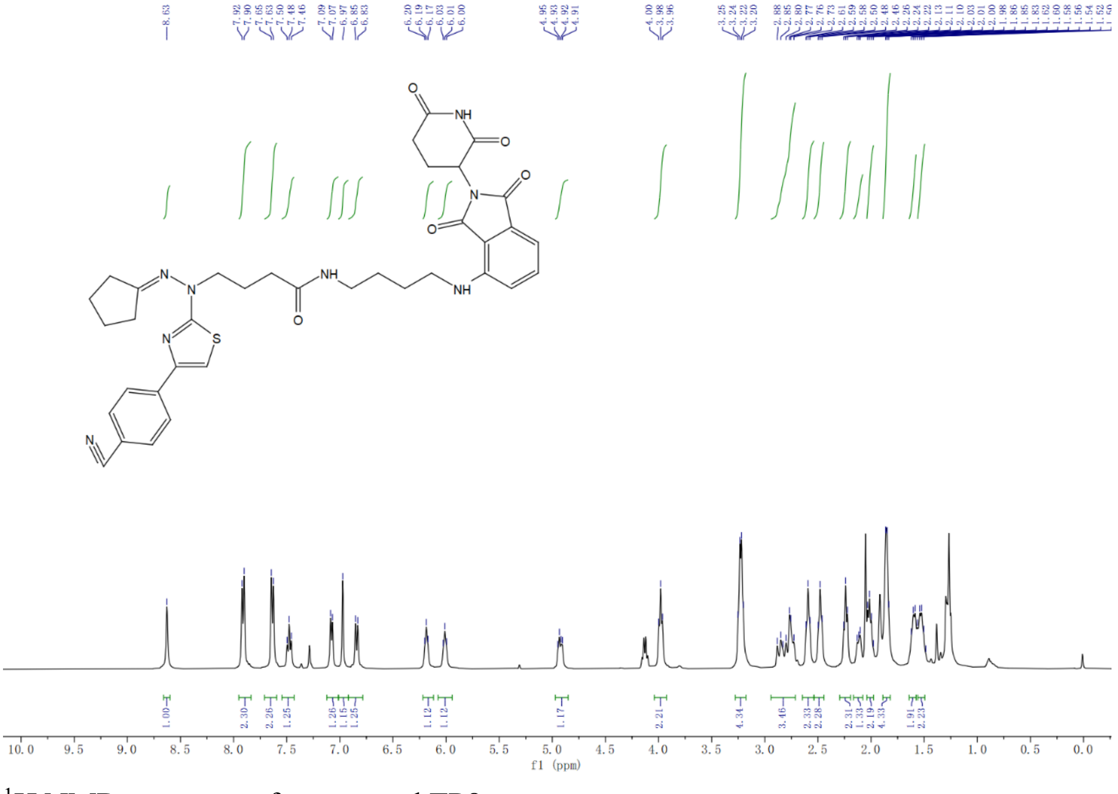

^{1}H NMR spectrum of compound TP8.

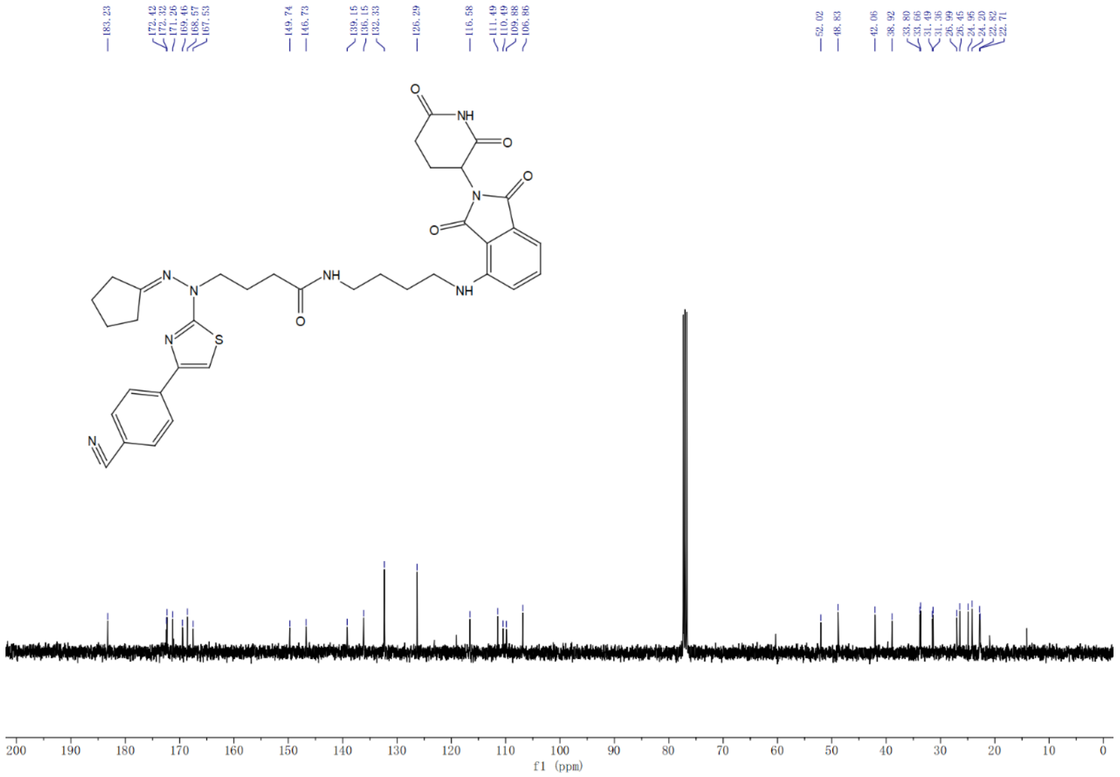

^{13}C NMR spectrum of compound TP8.

4-(1-(4-(4-Cyanophenyl)thiazol-2-yl)-2-cyclopentylidenehydrazineyl)-N-(5-((2-(2,6-dioxopiperidin-3-yl)-1,3-dioxoisoindolin-4-yl)amino)pentyl)butanamide (TP9)

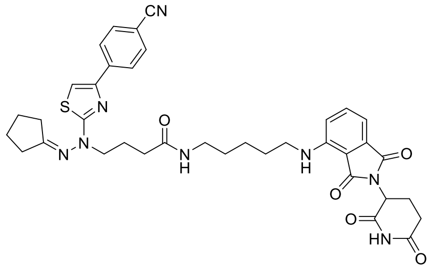

^{1}H NMR (400 MHz, Chloroform-*d*) δ 8.48 (s, 1H), 7.93 (d, *J* = 8.1 Hz, 2H), 7.66 (d, *J* = 8.2 Hz, 2H), 7.50 (t, *J* = 8.0 Hz, 1H), 7.10 (d, *J* = 7.2 Hz, 1H), 6.97 (s, 1H), 6.86 (d, *J* = 8.5 Hz, 1H), 6.20 (d, *J* = 6.0 Hz, 1H), 5.87 (d, *J* = 6.1 Hz, 1H), 4.93 (dd, *J* = 12.0, 5.6 Hz, 1H), 3.99 (t, *J* = 7.2 Hz, 2H), 3.22 (dq, *J* = 14.3, 6.9 Hz, 4H), 2.91–2.69 (m, 3H), 2.60 (d, *J* = 6.9 Hz, 2H), 2.48 (d, *J* = 7.3 Hz, 2H), 2.24 (t, *J* = 7.2 Hz, 2H), 2.18–2.08 (m, 1H), 2.02 (t, *J* = 7.2 Hz, 2H), 1.91–1.83 (m, 4H), 1.80 (s, 2H), 1.63 (p, *J* = 7.4 Hz, 2H), 1.47 (p, *J* = 6.5, 5.8 Hz, 2H). ^{13}C NMR (101 MHz, Chloroform-*d*) δ 183.4, 172.4, 172.2, 171.1, 169.5, 168.4, 167.5, 149.8, 146.8, 139.2, 136.1, 132.3, 126.3, 119.0, 116.6, 111.4, 110.5, 109.9, 106.8, 52.0, 48.8, 42.4, 39.2, 33.8, 33.7, 31.5, 31.4, 29.3, 28.7, 25.0, 24.2, 24.1, 22.8, 22.7. RT = 15.93 min.

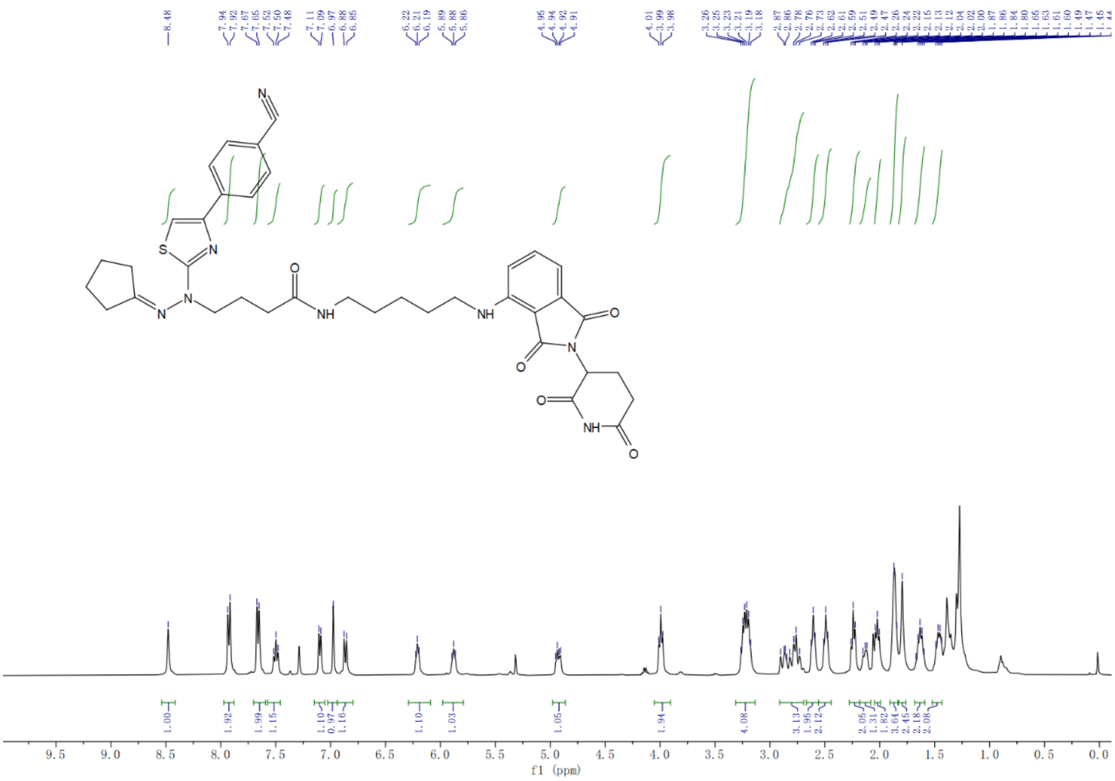

^{1}H NMR spectrum of compound TP9.

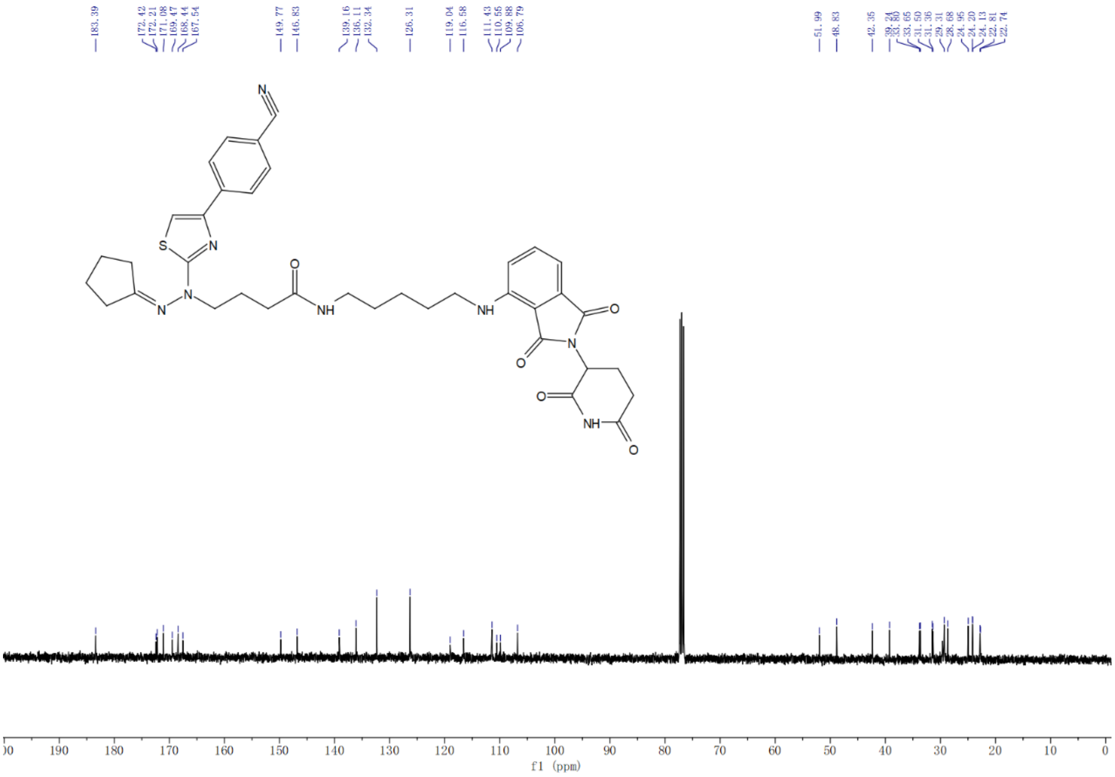

^{13}C NMR spectrum of compound TP9.

4-(1-(4-(4-Cyanophenyl)thiazol-2-yl)-2-cyclopentylidenehydrazineyl)-N-(6-((2-(2,6-dioxopiperidin-3-yl)-1,3-dioxoisoindolin-4-yl)amino)hexyl)butanamide

(TP10)

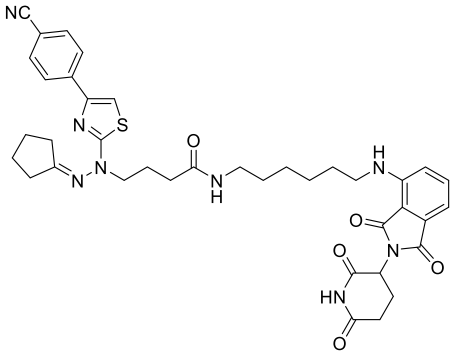

^{1}H NMR (400 MHz, Chloroform-*d*) δ 8.43 (s, 1H), 7.93 (d, *J* = 8.1 Hz, 2H), 7.66 (d, *J* = 8.1 Hz, 2H), 7.50 (t, *J* = 7.9 Hz, 1H), 7.10 (d, *J* = 7.3 Hz, 1H), 6.98 (s, 1H), 6.89 (t, *J* = 7.8 Hz, 1H), 6.21 (d, *J* = 5.9 Hz, 1H), 5.84 (t, *J* = 5.8 Hz, 1H), 4.93 (dd, *J* = 12.0, 5.5 Hz, 1H), 3.99 (t, *J* = 7.0 Hz, 2H), 3.22 (dq, *J* = 25.1, 6.7 Hz, 4H), 2.92–2.72 (m, 3H), 2.60 (d, *J* = 7.0 Hz, 2H), 2.48 (d, *J* = 7.2 Hz, 2H), 2.24 (t, *J* = 7.2 Hz, 2H), 2.16–2.10 (m, 1H), 2.02 (t, *J* = 7.1 Hz, 2H), 1.91–1.75 (m, 8H), 1.66–1.59 (m, 2H), 1.43 (d, *J* = 6.9 Hz, 2H). ^{13}C NMR (101 MHz, Chloroform-*d*) δ 183.3, 172.4, 172.2, 171.1, 169.5, 168.4, 167.6, 149.8, 146.9, 139.2, 136.1, 132.4, 132.3, 126.3, 119.0, 116.6, 111.4, 110.5, 109.8, 106.8, 52.0, 48.8, 42.4, 39.4, 33.8, 31.4, 31.4, 30.1, 29.6, 29.5, 29.0, 26.5, 25.0, 24.2, 22.8, 22.7. RT = 18.51 min.

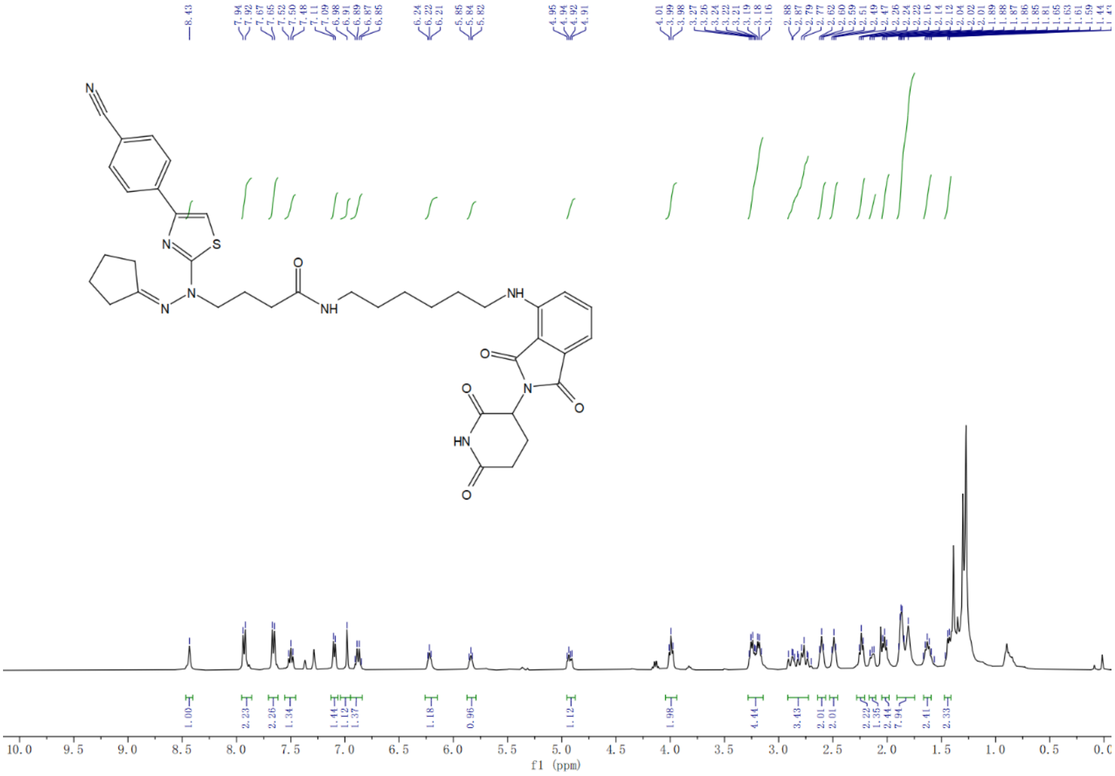

^{1}H NMR spectrum of compound TP10.

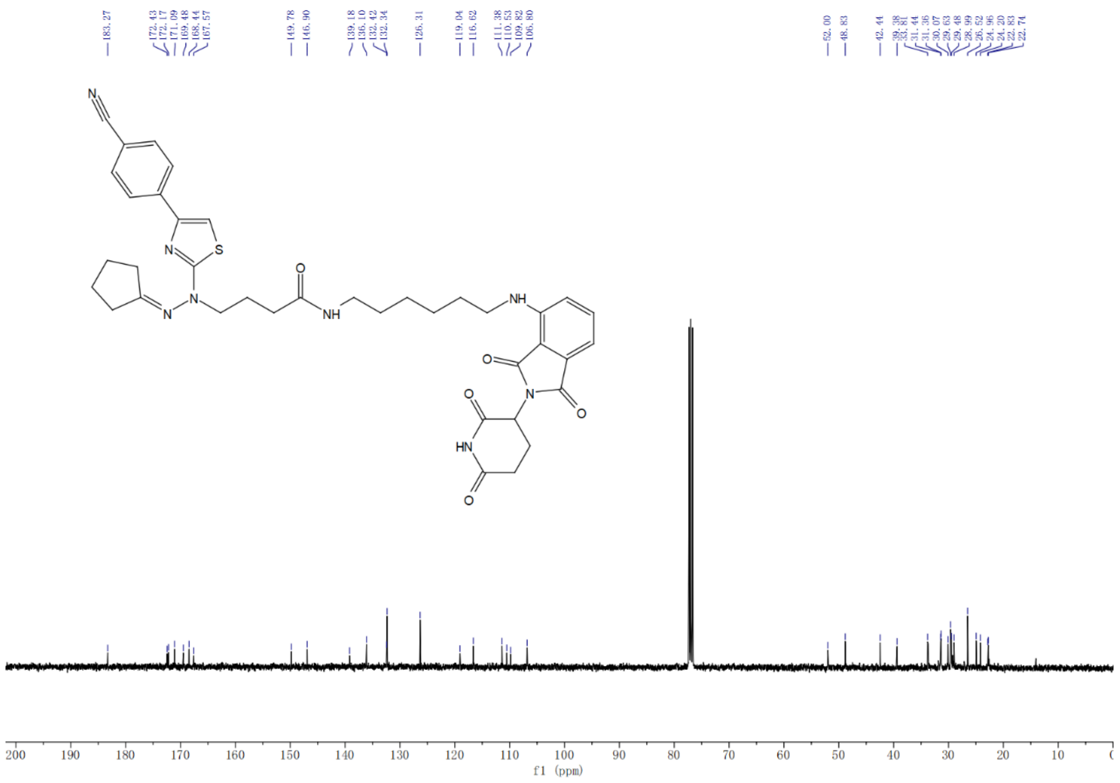

^{13}C NMR spectrum of compound TP10.

5-(1-(4-(4-Cyanophenyl)thiazol-2-yl)-2-cyclopentylidenehydrazineyl)-N-(2-((2-(2,6-dioxopiperidin-3-yl)-1,3-dioxoisoindolin-4-yl)amino)ethyl)pentanamide (TP11)

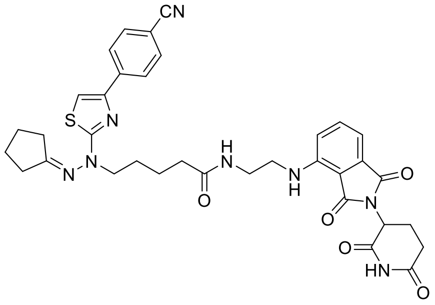

^{1}H NMR (400 MHz, Chloroform-*d*) δ 8.77 (s, 1H), 7.92 (d, *J* = 8.0 Hz, 2H), 7.64 (d, *J* = 8.0 Hz, 2H), 7.48 (t, *J* = 7.8 Hz, 1H), 7.09 (d, *J* = 7.1 Hz, 1H), 6.97 (s, 1H), 6.93 (d, *J* = 8.6 Hz, 1H), 6.35 (d, *J* = 5.8 Hz, 1H), 6.25 (d, *J* = 5.8 Hz, 1H), 4.93 (dd, *J* = 11.8, 5.4 Hz, 1H), 3.91 (t, *J* = 6.7 Hz, 2H), 3.37 (s, 4H), 2.88–2.70 (m, 3H), 2.63–2.53 (m, 2H), 2.51–2.43 (m, 2H), 2.22 (t, *J* = 6.8 Hz, 2H), 2.13–2.07 (m, 1H), 1.91 (s, 1H), 1.86 (q, *J* = 4.2 Hz, 4H), 1.68 (dt, *J* = 8.7, 4.5 Hz, 3H). ^{13}C NMR (101 MHz, Chloroform-*d*) δ 183.4, 173.5, 172.2, 171.4, 169.4, 168.8, 167.5, 149.7, 146.7, 139.1, 136.2, 132.4, 126.2, 116.7, 111.9, 110.4, 110.1, 106.8, 52.3, 48.9, 42.0, 38.9, 35.8, 33.8, 31.5, 31.4, 26.0, 24.9, 24.2, 23.0, 22.7. RT = 15.43 min.

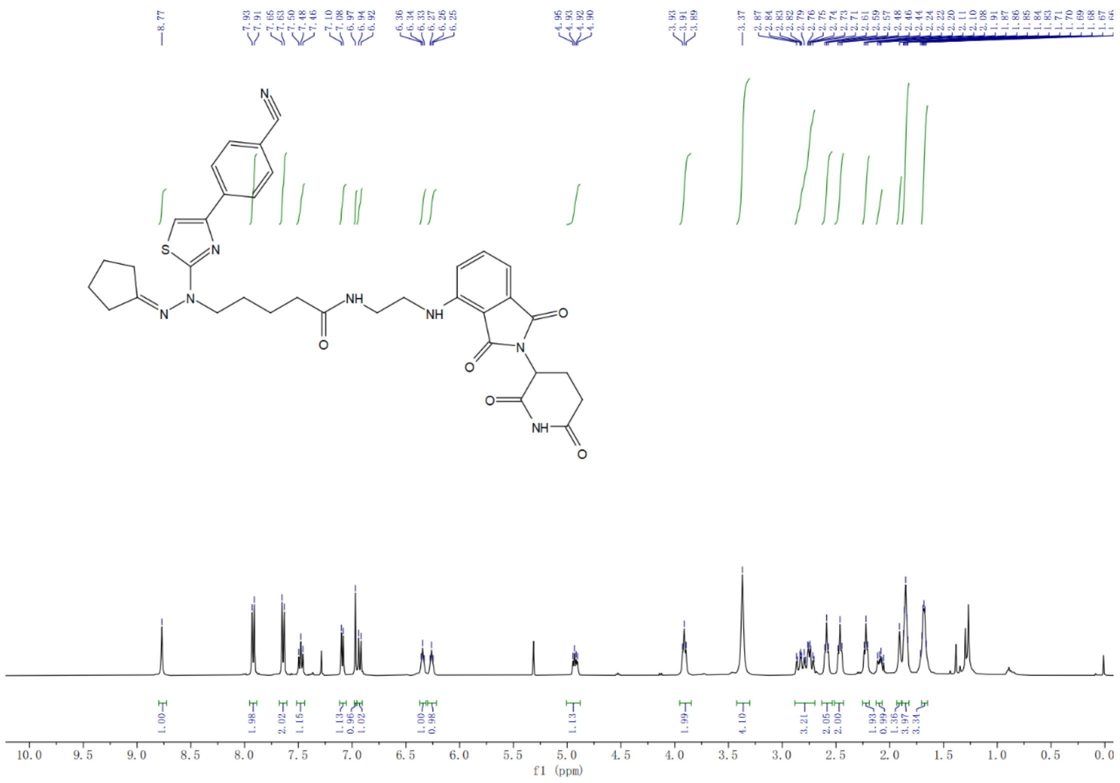

^{1}H NMR spectrum of compound TP11.

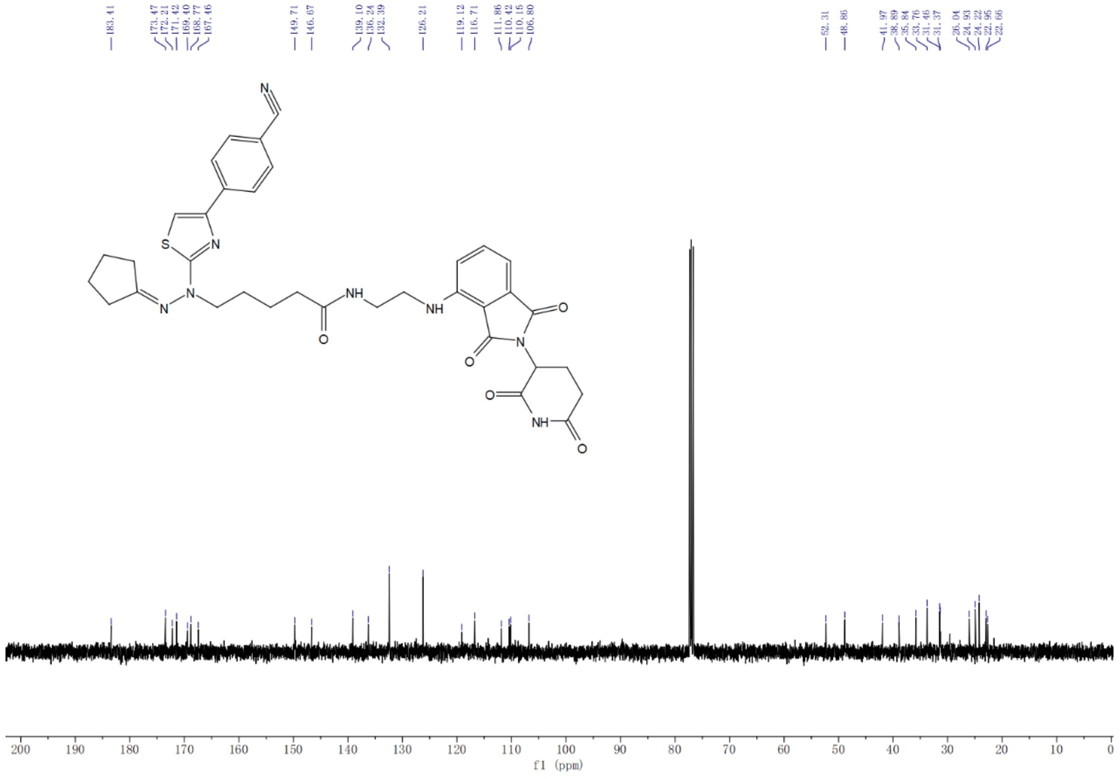

^{13}C NMR spectrum of compound TP11.

5-(1-(4-(4-Cyanophenyl)thiazol-2-yl)-2-cyclopentylidenehydrazineyl)-N-(4-((2-(2,6-dioxopiperidin-3-yl)-1,3-dioxoisoindolin-4-yl)amino)butyl)pentanamide

(TP13)

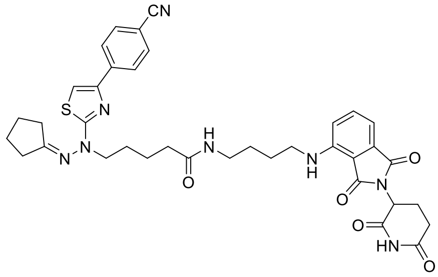

^{1}H NMR (400 MHz, Chloroform-*d*) δ 8.58 (s, 1H), 7.93 (d, *J* = 8.0 Hz, 2H), 7.65 (d, *J* = 8.1 Hz, 2H), 7.49 (t, *J* = 7.9 Hz, 1H), 7.09 (d, *J* = 7.1 Hz, 1H), 6.98 (s, 1H), 6.86 (d, *J* = 8.7 Hz, 1H), 6.19 (t, *J* = 5.8 Hz, 1H), 5.73 (t, *J* = 6.0 Hz, 1H), 4.93 (dd, *J* = 11.8, 5.3 Hz, 1H), 3.93 (t, *J* = 6.5 Hz, 2H), 3.22 (dq, *J* = 13.3, 6.4 Hz, 4H), 2.91–2.82 (m, 1H), 2.76 (tt, *J* = 10.1, 3.9 Hz, 2H), 2.60 (d, *J* = 7.1 Hz, 2H), 2.46 (d, *J* = 7.1 Hz, 2H), 2.22 (t, *J* = 6.9 Hz, 2H), 2.16–2.08 (m, 1H), 1.88 (d, *J* = 13.8 Hz, 4H), 1.69 (s, 4H), 1.60 (q, *J* = 7.1, 6.5 Hz, 2H), 1.52 (q, *J* = 7.8 Hz, 2H). ^{13}C NMR (101 MHz, Chloroform-*d*) δ 183.2, 172.8, 172.3, 171.2, 169.5, 168.6, 167.5, 149.7, 146.8, 139.1, 136.2, 132.4, 126.2, 116.6, 111.5, 110.4, 109.9, 106.8, 52.2, 48.8, 42.1, 38.8, 36.1, 33.8, 31.5, 31.4, 27.0, 26.4, 26.0, 24.9, 24.2, 23.1, 22.7. RT = 15.77 min.

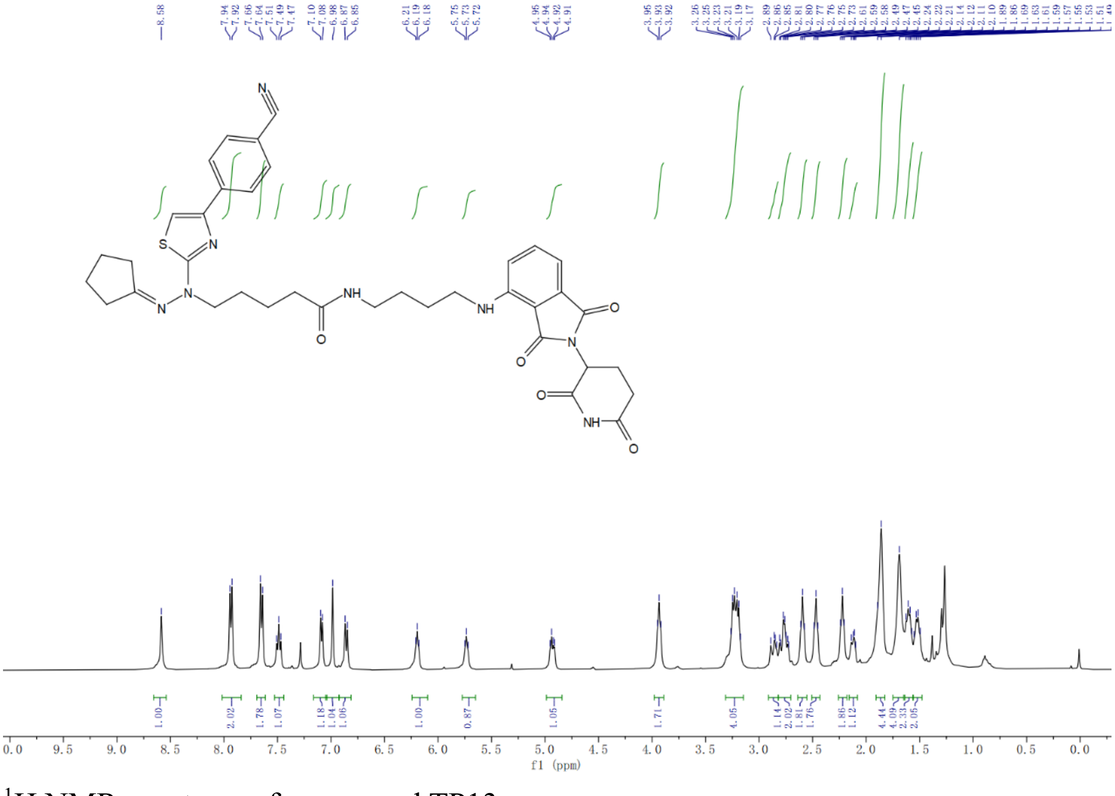

^{1}H NMR spectrum of compound TP13.

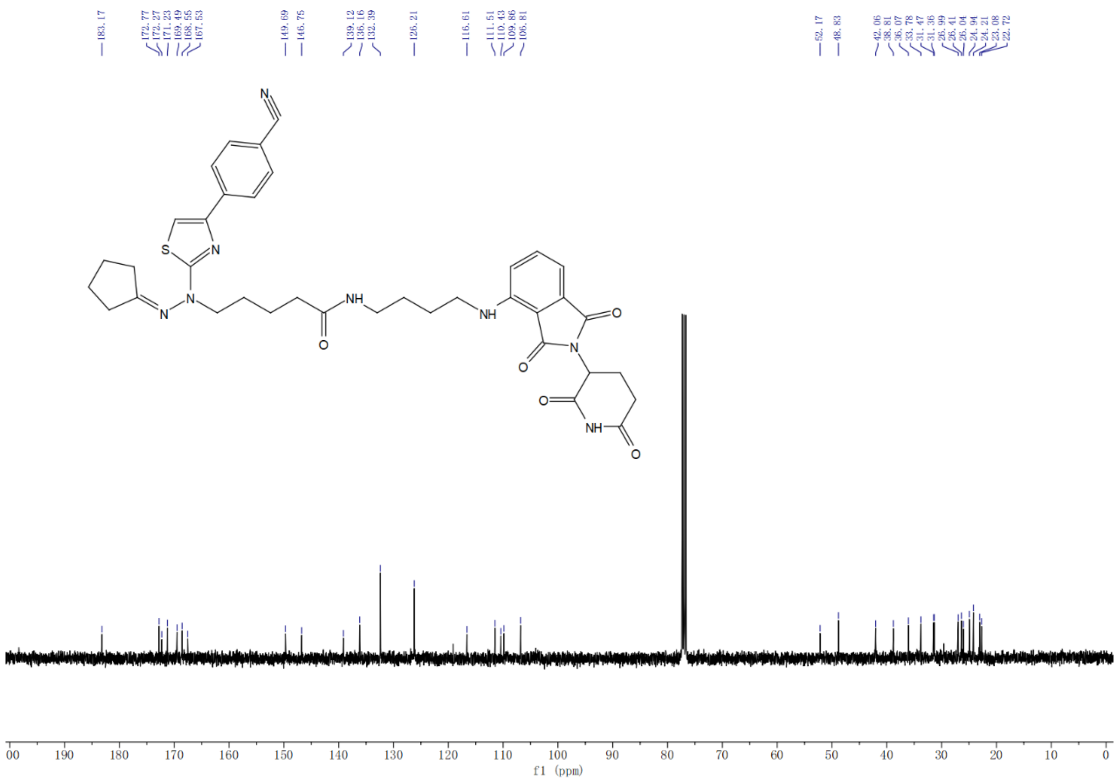

^{13}C NMR spectrum of compound TP13.

5-(1-(4-(4-Cyanophenyl)thiazol-2-yl)-2-cyclopentylidenehydrazineyl)-N-(5-((2-(2,6-dioxopiperidin-3-yl)-1,3-dioxoisoindolin-4-yl)amino)pentyl)pentanamide (TP14)

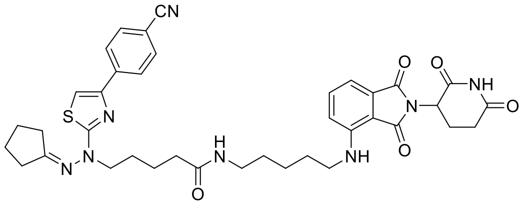

^{1}H NMR (400 MHz, Chloroform-*d*) δ 8.54 (s, 1H), 7.94 (d, *J* = 8.1 Hz, 2H), 7.66 (d, *J* = 8.0 Hz, 2H), 7.49 (t, *J* = 8.0 Hz, 1H), 7.09 (d, *J* = 7.2 Hz, 1H), 6.98 (s, 1H), 6.86 (d, *J* = 8.5 Hz, 1H), 6.21 (t, *J* = 5.8 Hz, 1H), 5.63 (d, *J* = 6.3 Hz, 1H), 4.93 (dd, *J* = 12.0, 5.4 Hz, 1H), 3.93 (d, *J* = 7.0 Hz, 2H), 3.21 (dp, *J* = 27.0, 6.8 Hz, 4H), 2.81 (dq, *J* = 35.3, 12.3 Hz, 3H), 2.59 (d, *J* = 6.7 Hz, 2H), 2.46 (d, *J* = 7.2 Hz, 2H), 2.21 (d, *J* = 6.5 Hz, 2H), 2.16–2.09 (m, 1H), 1.86 (s, 4H), 1.75–1.67 (m, 4H), 1.62 (d, *J* = 7.3 Hz, 2H), 1.50–1.41 (m, 2H), 1.37 (d, *J* = 8.3 Hz, 2H). ^{13}C NMR (101 MHz, Chloroform-*d*) δ 183.3, 172.7, 172.3, 171.2, 169.5, 168.5, 167.6, 149.7, 146.8, 139.1, 136.1, 132.4, 126.2, 116.6, 111.4, 110.4, 109.8, 106.8, 52.2, 48.8, 42.3, 39.1, 36.1, 33.8, 31.5, 31.4, 29.3, 28.7, 26.0, 24.9, 24.2, 24.1, 23.1, 22.7. RT = 16.10 min.

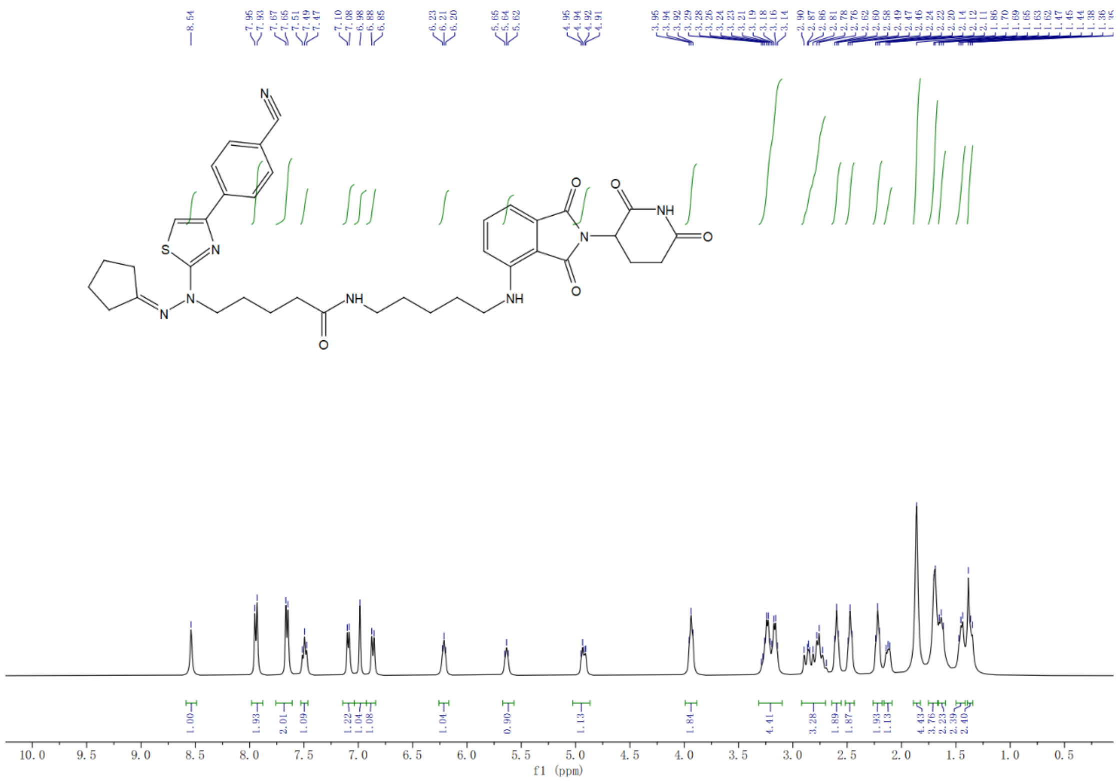

^{1}H NMR spectrum of compound TP14.

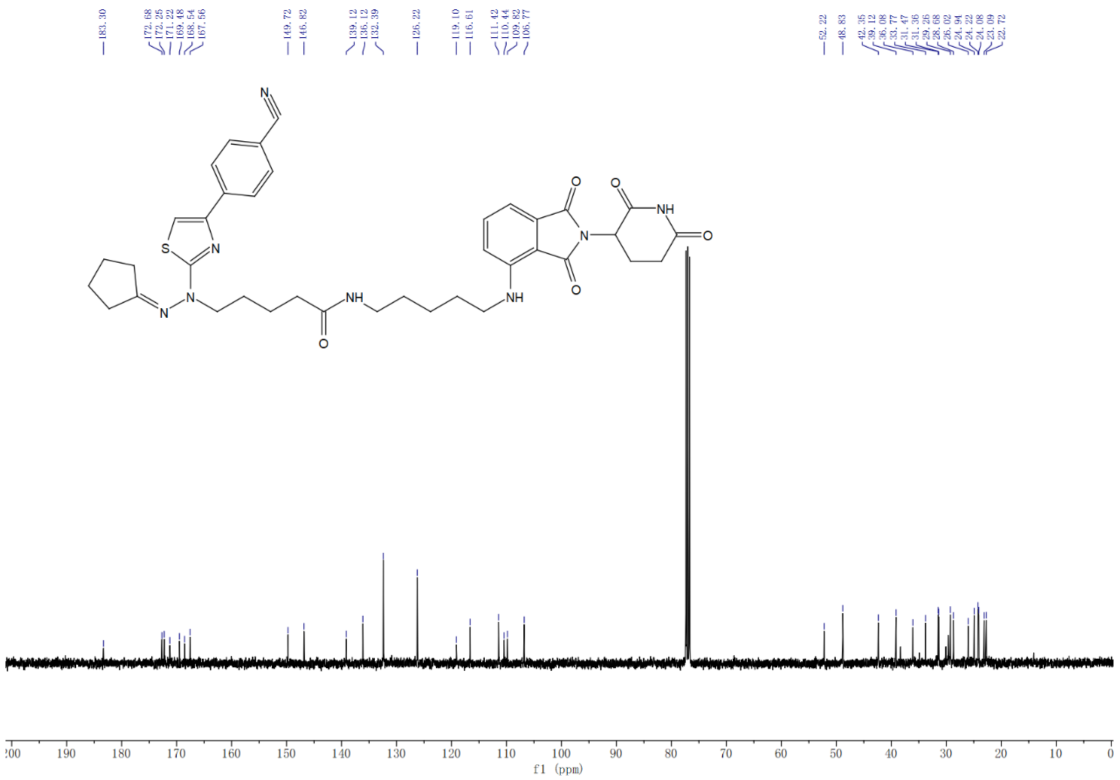

^{13}C NMR spectrum of compound TP14.

5-(1-(4-(4-Cyanophenyl)thiazol-2-yl)-2-cyclopentylidenehydrazineyl)-N-(6-((2-(2,6-dioxopiperidin-3-yl)-1,3-dioxoisoindolin-4-yl)amino)hexyl)pentanamide

(TP15)

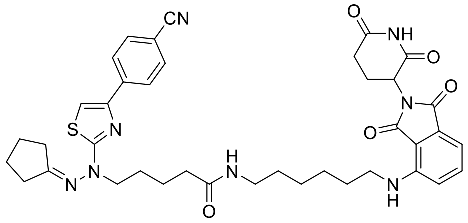

^{1}H NMR (400 MHz, Chloroform-*d*) δ 8.38 (s, 1H), 7.95 (d, *J* = 7.9 Hz, 2H), 7.67 (d, *J* = 8.0 Hz, 2H), 7.50 (t, *J* = 7.9 Hz, 1H), 7.10 (d, *J* = 7.1 Hz, 1H), 6.99 (s, 1H), 6.88 (d, *J* = 8.6 Hz, 1H), 6.23 (s, 1H), 5.56 (s, 1H), 5.00–4.87 (m, 1H), 3.93 (d, *J* = 7.9 Hz, 2H), 3.25 (q, *J* = 6.8 Hz, 2H), 3.16 (q, *J* = 7.1 Hz, 2H), 2.89 (d, *J* = 15.3 Hz, 1H), 2.77 (p, *J* = 14.0 Hz, 2H), 2.61 (d, *J* = 7.3 Hz, 2H), 2.47 (d, *J* = 7.3 Hz, 2H), 2.23 (d, *J* = 7.0 Hz, 2H), 2.16–2.07 (m, 1H), 1.87 (s, 4H), 1.79 (s, 2H), 1.67 (d, *J* = 24.1 Hz, 6H), 1.46–1.37 (m, 4H). ^{13}C NMR (101 MHz, Chloroform-*d*) δ 183.0, 172.6, 172.3, 171.1, 169.5, 168.4, 167.6, 149.7, 146.9, 139.1, 136.1, 132.4, 126.2, 116.6, 111.4, 110.5, 109.8, 106.8, 52.2, 48.8, 42.5, 39.3, 36.1, 33.8, 31.5, 31.4, 29.6, 29.5, 29.0, 26.5, 26.0, 25.0, 24.2, 23.1, 22.7. RT = 18.82 min.

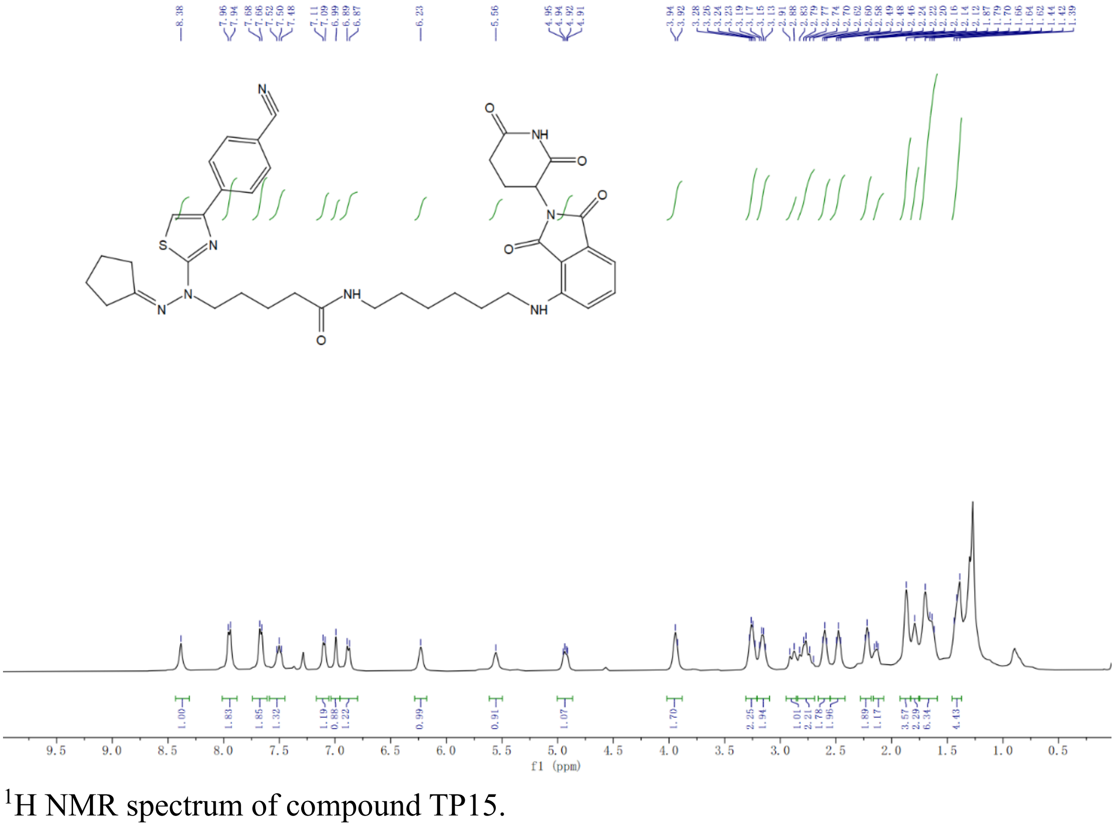

^{1}H NMR spectrum of compound TP15.

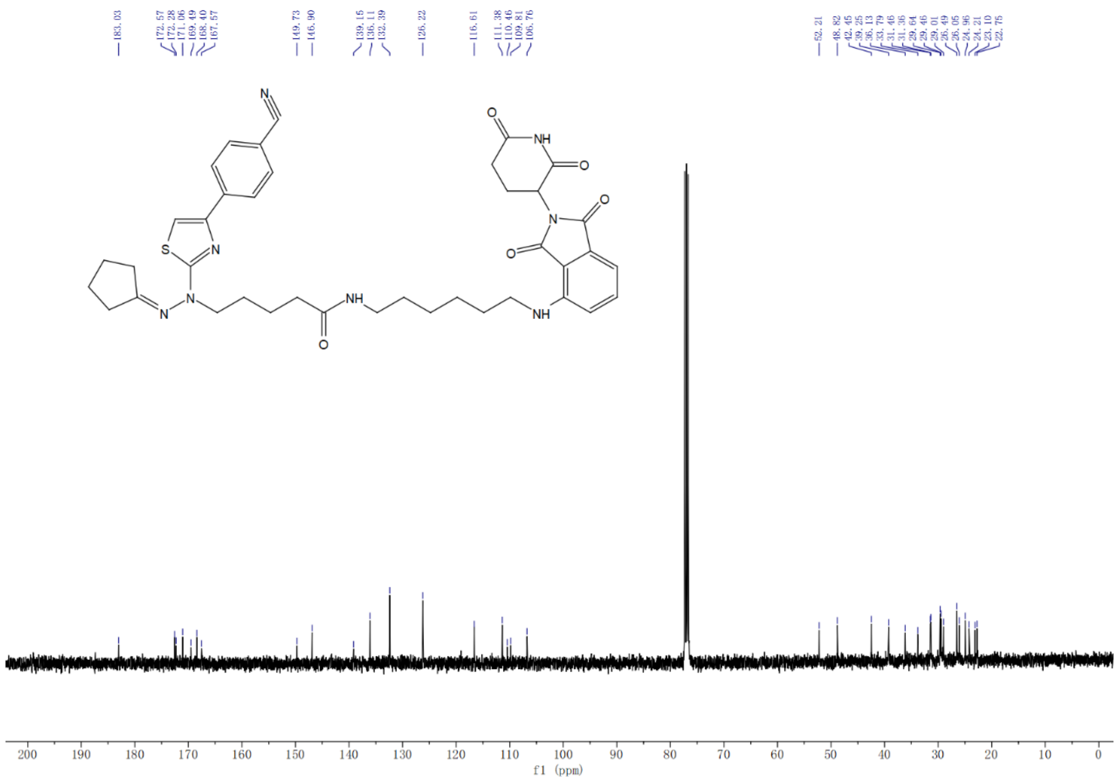

^{13}C NMR spectrum of compound TP15.

7-(1-(4-(4-Cyanophenyl)thiazol-2-yl)-2-cyclopentylidenehydrazineyl)-N-(2-((2-(2,6-dioxopiperidin-3-yl)-1,3-dioxoisoindolin-4-yl)amino)ethyl)heptanamide (TP16)

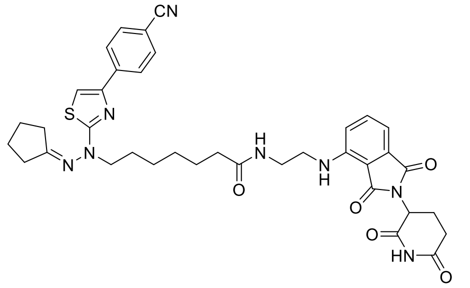

^{1}H NMR (400 MHz, Chloroform-*d*) δ 8.42 (s, 1H), 7.95 (d, *J* = 8.1 Hz, 2H), 7.66 (d, *J* = 8.0 Hz, 2H), 7.51 (t, *J* = 7.8 Hz, 1H), 7.12 (d, *J* = 7.2 Hz, 1H), 7.03–6.96 (m, 2H), 6.40 (d, *J* = 5.9 Hz, 1H), 5.98 (d, *J* = 5.2 Hz, 1H), 4.94 (dd, *J* = 12.0, 5.3 Hz, 1H), 3.89 (t, *J* = 7.5 Hz, 2H), 3.48 (d, *J* = 9.9 Hz, 4H), 2.92–2.75 (m, 3H), 2.65–2.57 (m, 2H), 2.52–2.43 (m, 2H), 2.14 (q, *J* = 7.7 Hz, 3H), 1.88 (d, *J* = 6.9 Hz, 4H), 1.63 (q, *J* = 8.0 Hz, 4H), 1.35 (t, *J* = 4.4 Hz, 4H). ^{13}C NMR (101 MHz, Chloroform-*d*) δ 183.0, 173.7, 172.2, 171.1, 169.4, 168.5, 167.4, 149.8, 146.7, 139.2, 136.3, 132.3, 126.3, 116.7, 111.9, 110.4, 106.6, 53.0, 48.9, 42.0, 39.0, 36.3, 33.7, 31.5, 31.4, 29.6, 26.6, 26.4, 25.4, 24.9, 24.2, 22.7. RT = 16.15 min.

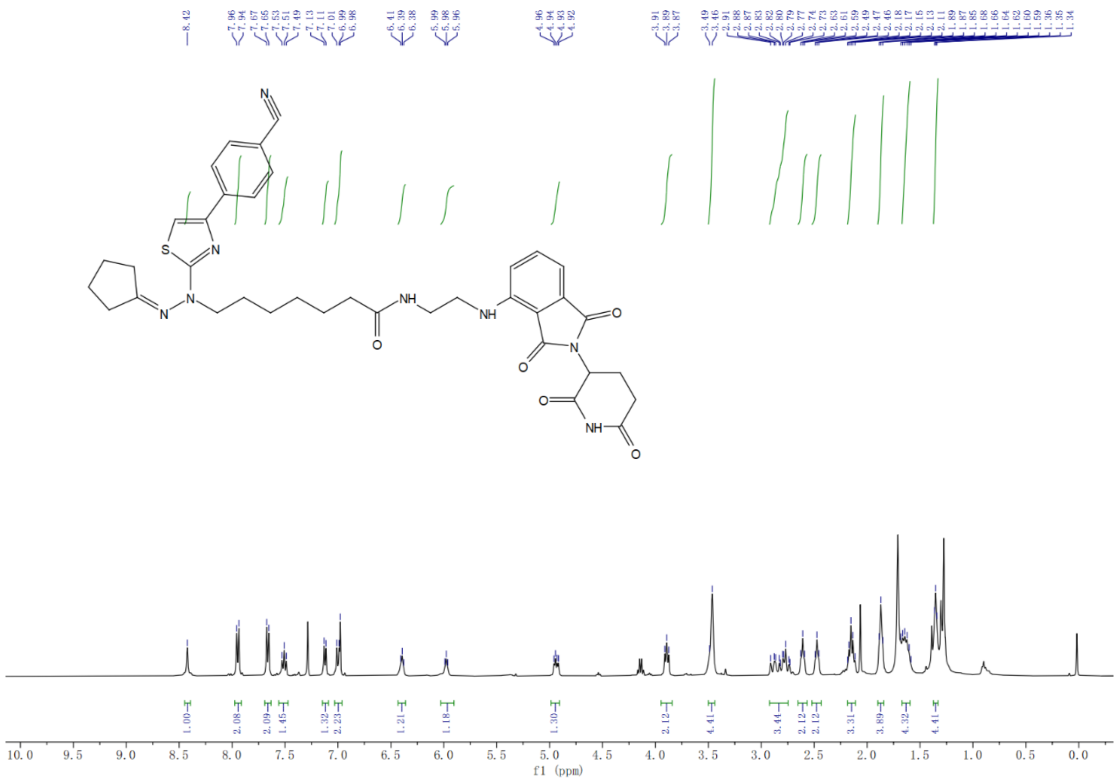

^{1}H NMR spectrum of compound TP16.

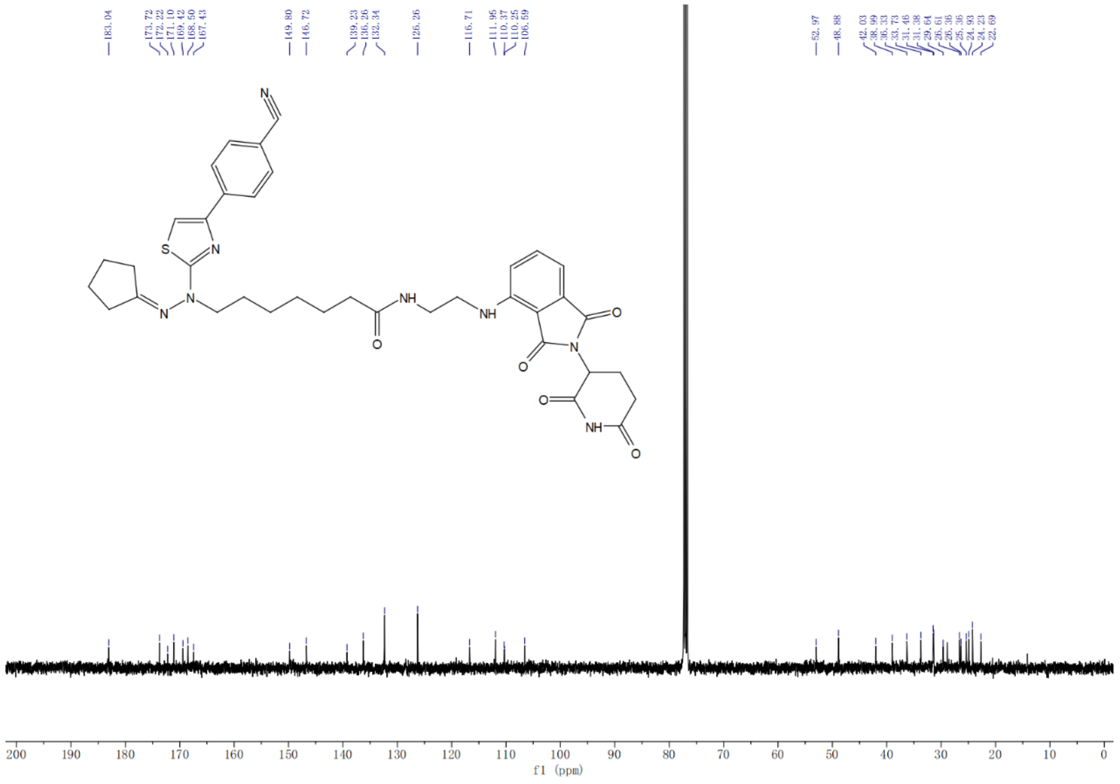

^{13}C NMR spectrum of compound TP16.

7-(1-(4-(4-Cyanophenyl)thiazol-2-yl)-2-cyclopentylidenehydrazineyl)-N-(3-((2-(2,6-dioxopiperidin-3-yl)-1,3-dioxoisoindolin-4-yl)amino)propyl)heptanamide

(TP17)

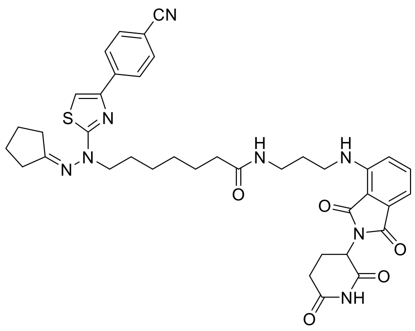

^{1}H NMR (400 MHz, Chloroform-*d*) δ 8.27 (s, 1H), 7.95 (d, *J* = 8.1 Hz, 2H), 7.67 (d, *J* = 7.9 Hz, 2H), 7.50 (t, *J* = 7.8 Hz, 1H), 7.11 (d, *J* = 7.1 Hz, 1H), 6.98 (s, 1H), 6.88 (d, *J* = 8.5 Hz, 1H), 6.41 (t, *J* = 5.8 Hz, 1H), 5.66 (t, *J* = 6.1 Hz, 1H), 4.94 (dd, *J* = 12.0, 5.3 Hz, 1H), 3.90 (t, *J* = 7.5 Hz, 2H), 3.35 (dq, *J* = 19.5, 6.4 Hz, 4H), 2.94–2.71 (m, 3H), 2.64–2.56 (m, 2H), 2.52–2.43 (m, 2H), 2.15 (q, *J* = 11.6, 9.7 Hz, 3H), 1.92–1.81 (m, 6H), 1.65 (q, *J* = 7.9, 7.3 Hz, 8H). ^{13}C NMR (101 MHz, Chloroform-*d*) δ 183.0, 173.4, 172.2, 171.0, 169.4, 168.3, 167.5, 149.8, 146.6, 139.2, 136.2, 132.3, 126.3, 116.5, 111.6, 110.4, 110.1, 106.6, 53.0, 48.8, 40.1, 36.9, 33.7, 31.5, 31.4, 30.1, 29.6, 29.4, 28.9, 26.6, 26.4, 25.5, 24.9, 24.2, 22.7. RT = 16.30 min.

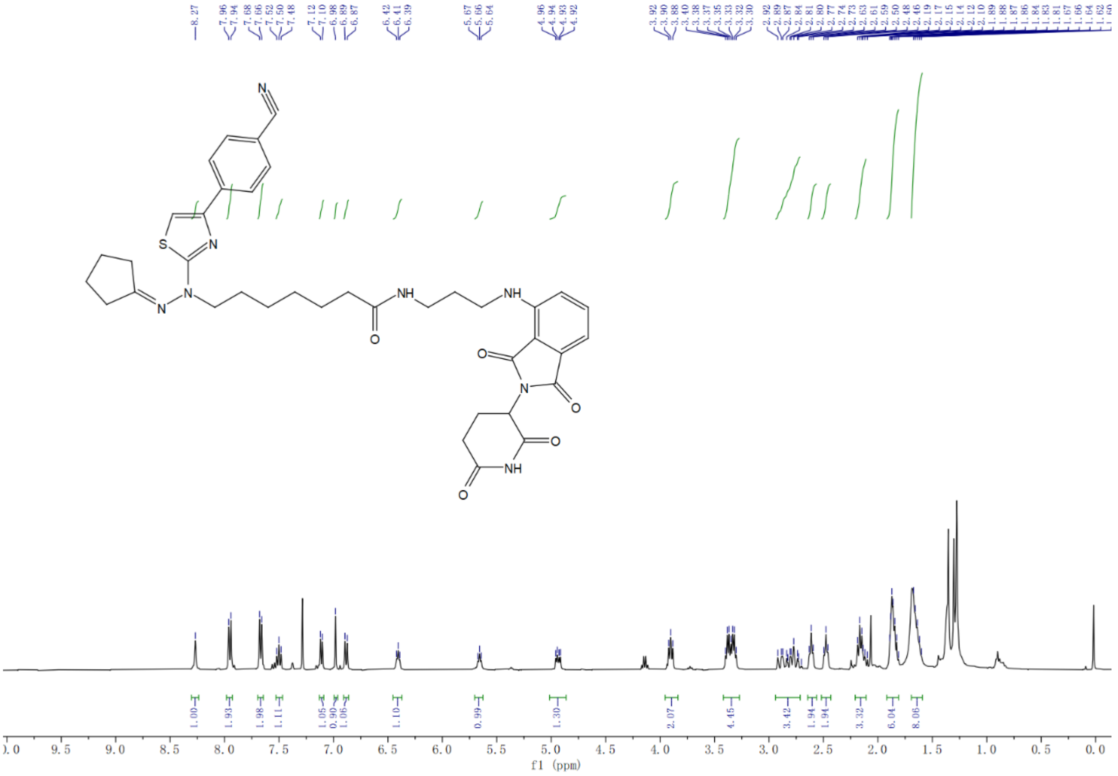

^{1}H NMR spectrum of compound TP17.

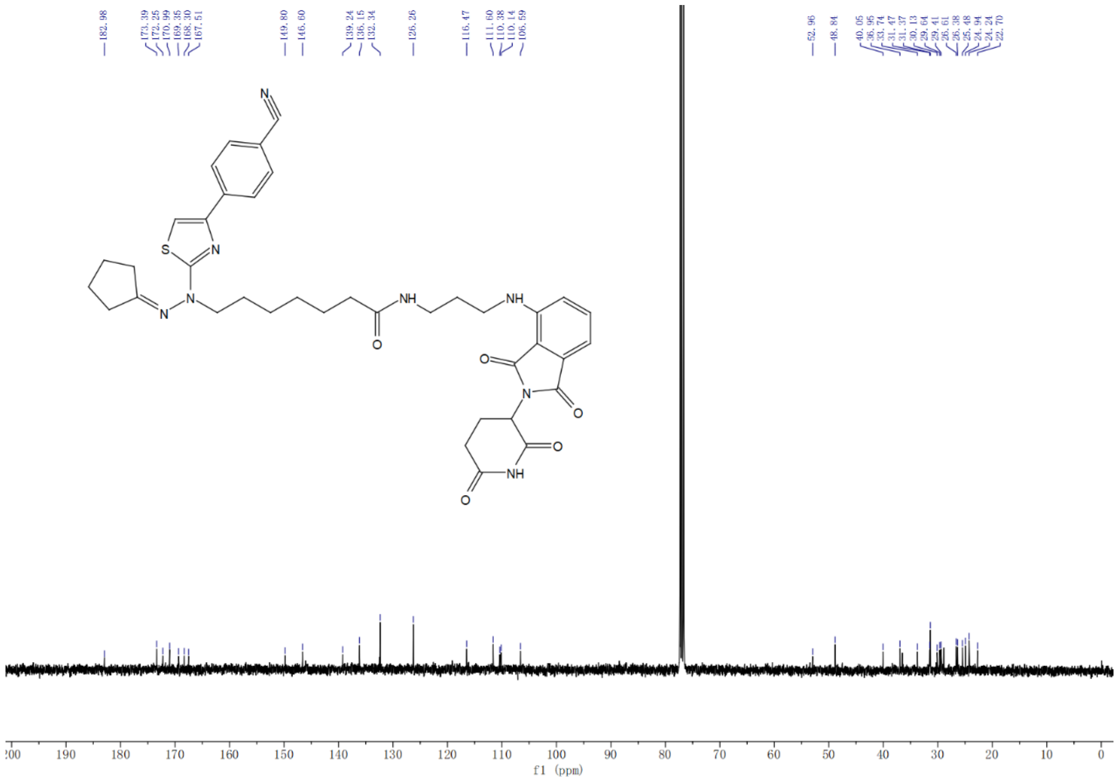

^{13}C NMR spectrum of compound TP17.

7-(1-(4-(4-Cyanophenyl)thiazol-2-yl)-2-cyclopentylidenehydrazineyl)-N-(4-((2-(2,6-dioxopiperidin-3-yl)-1,3-dioxoisoindolin-4-yl)amino)butyl)heptanamide (TP18)

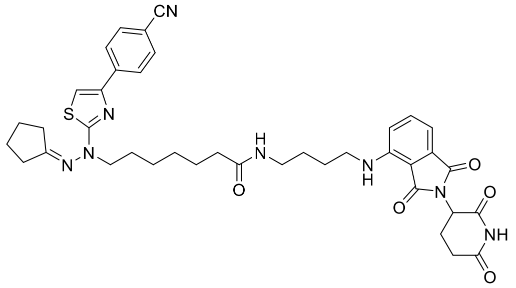

^{1}H NMR (400 MHz, Chloroform-*d*) δ 8.23 (s, 1H), 7.96 (d, *J* = 8.2 Hz, 2H), 7.67 (d, *J* = 7.7 Hz, 2H), 7.51 (t, *J* = 7.8 Hz, 1H), 7.11 (d, *J* = 7.0 Hz, 1H), 6.98 (s, 1H), 6.89 (d, *J* = 8.4 Hz, 1H), 6.32–6.15 (m, 1H), 5.51 (s, 1H), 4.93 (dd, *J* = 12.2, 5.4 Hz, 1H), 3.90 (t, *J* = 7.5 Hz, 2H), 3.31 (q, *J* = 6.4 Hz, 4H), 2.89 (t, *J* = 12.4 Hz, 1H), 2.77 (p, *J* = 14.1 Hz, 2H), 2.60 (d, *J* = 7.2 Hz, 2H), 2.47 (d, *J* = 6.9 Hz, 2H), 2.19–2.11 (m, 3H), 1.88 (d, *J* = 6.8 Hz, 4H), 1.72–1.65 (m, 8H), 1.36 (d, *J* = 4.9 Hz, 4H). ^{13}C NMR (101 MHz, Chloroform-*d*) δ 182.9, 173.0, 172.2, 171.0, 169.5, 168.4, 167.5, 146.8, 136.2, 132.3, 126.3, 116.6, 111.6, 110.4, 109.9, 106.6, 53.0, 48.8, 42.1, 38.8, 36.6, 33.7, 31.5, 31.4, 30.1, 29.6, 28.9, 27.1, 26.5, 24.9, 24.2, 23.2, 22.7. RT = 18.88 min.

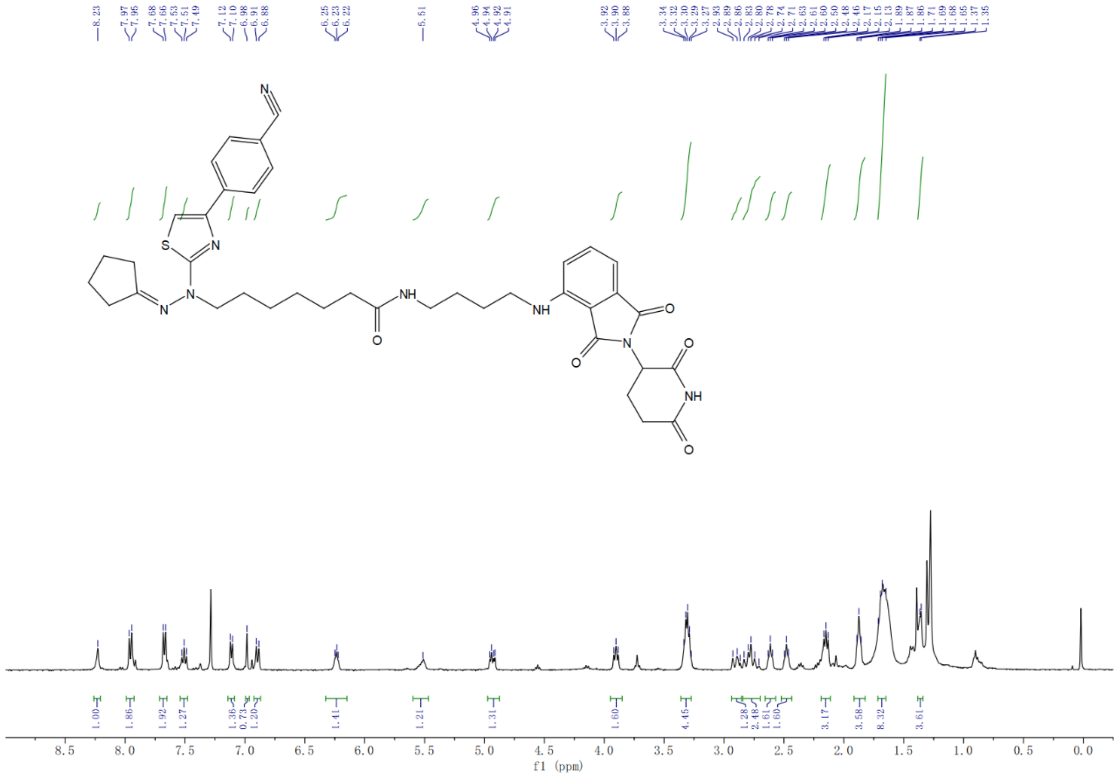

^{1}H NMR spectrum of compound TP18.

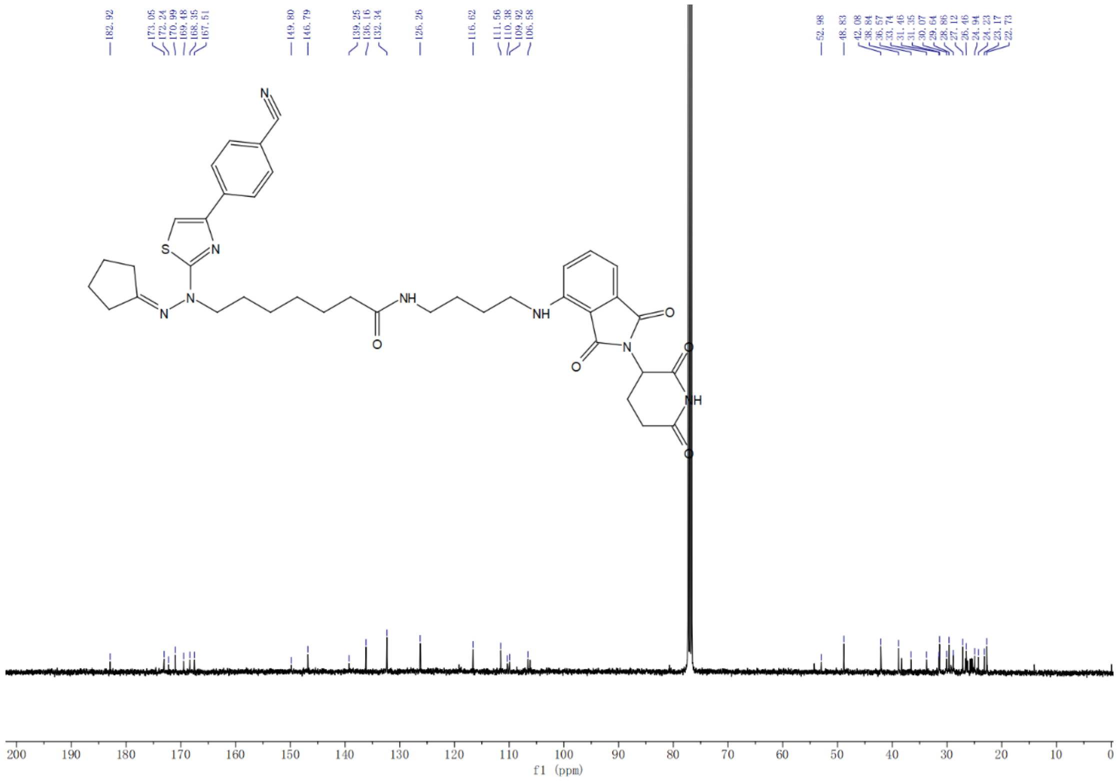

^{13}C NMR spectrum of compound TP18.

7-(1-(4-(4-Cyanophenyl)thiazol-2-yl)-2-cyclopentylidenehydrazineyl)-N-(5-((2-(2,6-dioxopiperidin-3-yl)-1,3-dioxoisoindolin-4-yl)amino)pentyl)heptanamide

(TP19)

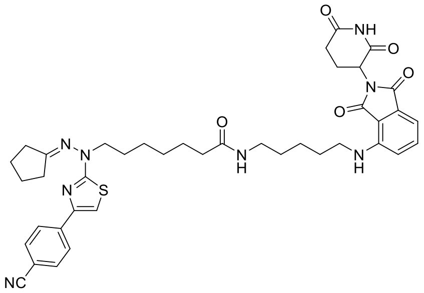

^{1}H NMR (400 MHz, Chloroform-*d*) δ 8.69 (s, 1H), 7.91 (d, *J* = 8.0 Hz, 2H), 7.63 (d, *J* = 8.0 Hz, 2H), 7.48 (t, *J* = 7.8 Hz, 1H), 7.08 (d, *J* = 6.9 Hz, 1H), 6.93 (s, 1H), 6.86 (d, *J* = 8.6 Hz, 1H), 6.21 (d, *J* = 5.9 Hz, 1H), 5.65 (d, *J* = 6.1 Hz, 1H), 4.92 (dd, *J* = 12.1, 5.4 Hz, 1H), 3.70 (t, *J* = 7.3 Hz, 2H), 3.25 (q, *J* = 6.9 Hz, 6H), 2.90–2.79 (m, 2H), 2.74 (dd, *J* = 15.5, 5.5 Hz, 2H), 2.14 (dt, *J* = 14.2, 6.6 Hz, 4H), 1.78–1.70 (m, 2H), 1.70–1.61 (m, 6H), 1.56–1.51 (m, 2H), 1.40 (t, *J* = 7.1 Hz, 8H). ^{13}C NMR (101 MHz, Chloroform-*d*) δ 174.6, 173.1, 171.4, 169.5, 168.7, 167.6, 149.9, 146.9, 139.3, 136.1, 132.3, 126.2, 119.2, 116.6, 111.4, 110.2, 109.8, 106.2, 54.2, 48.8, 42.4, 39.2, 36.5, 31.4, 30.1, 29.6, 29.3, 28.8, 28.7, 26.2, 25.7, 25.4, 24.1, 22.7. RT = 15.29 min.

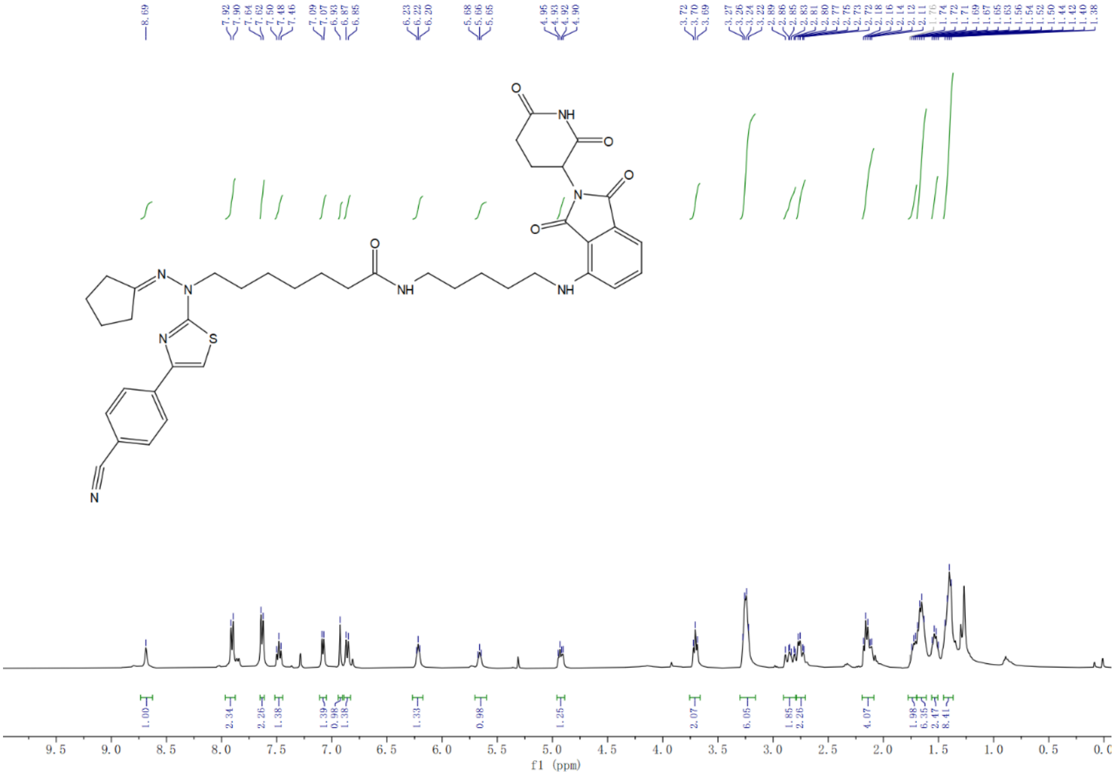

^{1}H NMR spectrum of compound TP19.

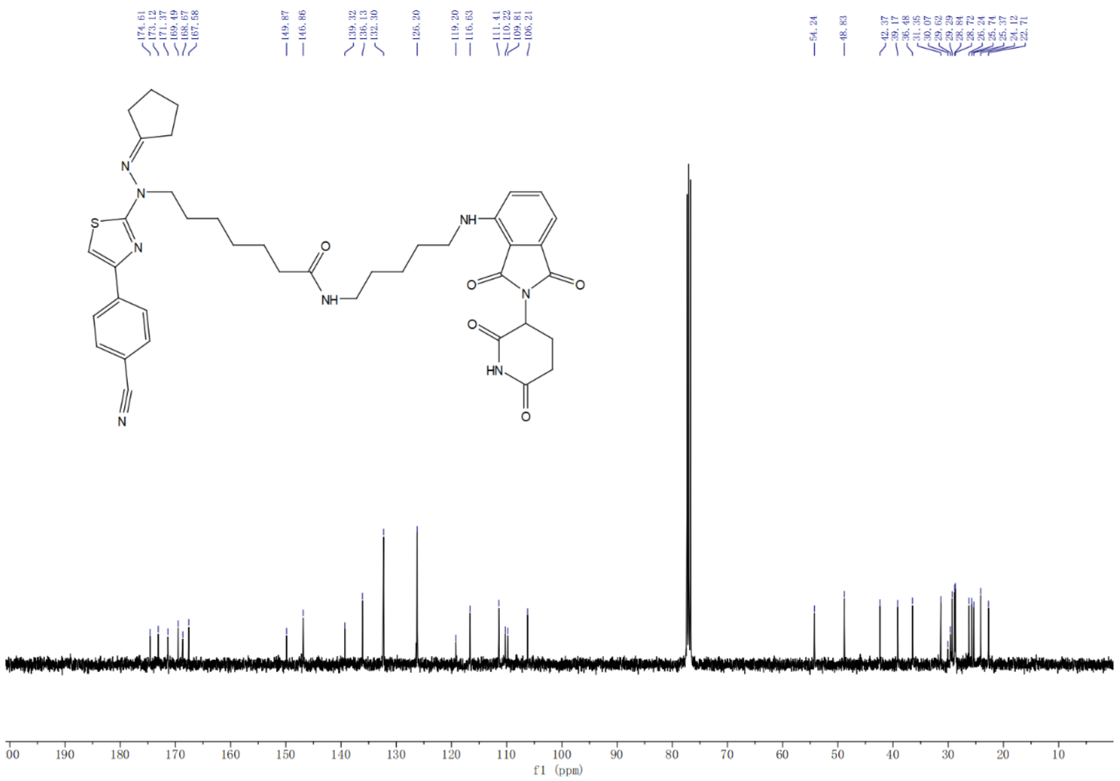

^{13}C NMR spectrum of compound TP19.

7-(1-(4-(4-Cyanophenyl)thiazol-2-yl)-2-cyclopentylidenehydrazineyl)-N-(6-((2-(2,6-dioxopiperidin-3-yl)-1,3-dioxoisoindolin-4-yl)amino)hexyl)heptanamide (TP20)

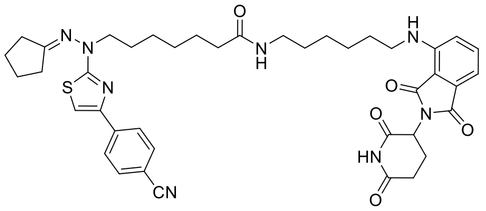

^{1}H NMR (400 MHz, Chloroform-*d*) δ 8.47 (s, 1H), 7.91 (d, *J* = 8.2 Hz, 2H), 7.64 (d, *J* = 8.1 Hz, 2H), 7.49 (t, *J* = 7.8 Hz, 1H), 7.09 (d, *J* = 7.1 Hz, 1H), 6.93 (s, 1H), 6.87 (d, *J* = 8.5 Hz, 1H), 6.22 (t, *J* = 5.6 Hz, 1H), 4.92 (dd, *J* = 11.9, 5.4 Hz, 1H), 3.71 (t, *J* = 7.2 Hz, 2H), 3.24 (q, *J* = 6.6 Hz, 4H), 2.92–2.70 (m, 4H), 2.15 (dt, *J* = 11.0, 6.4 Hz, 4H), 1.74 (q, *J* = 7.0 Hz, 2H), 1.66 (p, *J* = 7.4 Hz, 6H), 1.51 (t, *J* = 7.2 Hz, 2H), 1.40 (tdt, *J* = 15.3, 11.3, 5.7 Hz, 12H). ^{13}C NMR (101 MHz, Chloroform-*d*) δ 174.6, 173.0, 171.2, 169.5, 168.5, 167.6, 149.9, 146.9, 139.3, 136.1, 132.4, 132.3, 126.2, 119.2, 116.6, 111.4, 110.2, 109.8, 106.2, 60.4, 54.3, 48.8, 42.4, 39.3, 36.5, 31.3, 29.6, 29.5, 29.0, 28.8, 26.5, 26.2, 25.7, 25.4, 22.7, 14.1. RT = 15.73 min.

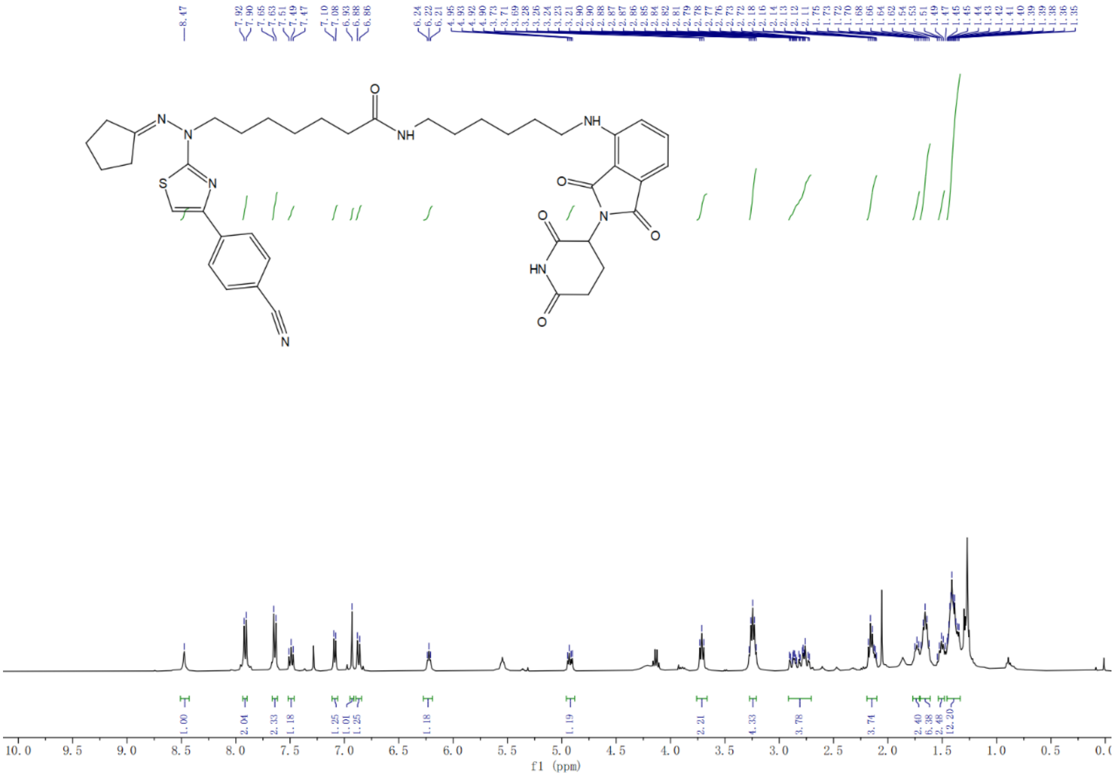

^{1}H NMR spectrum of compound TP20.

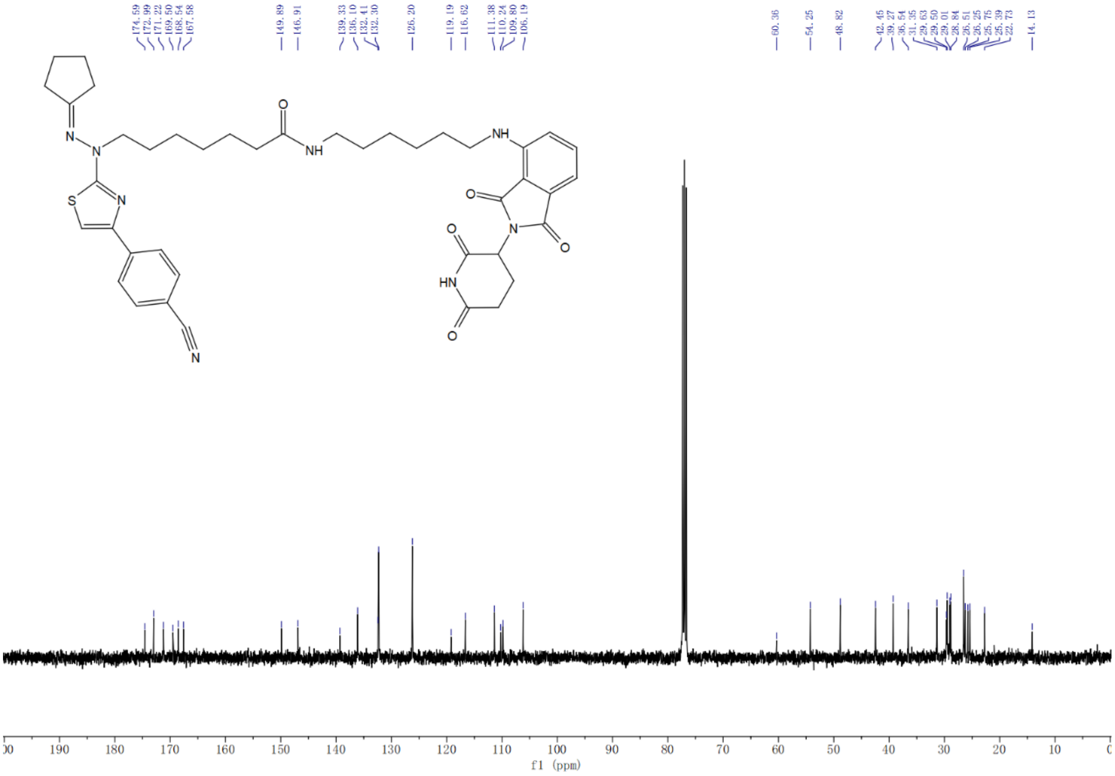

^{13}C NMR spectrum of compound TP20.

General procedure for the synthesis of compounds **TP21–23**

These compounds were prepared in a similar manner as NP1192 but with intermediates

4–6 replacing intermediate 7 to obtain a yellow solid (35.7-49.7% yield).

4-(2-(2-Cyclopentylidene-1-(2-(4-(2-(2,6-dioxopiperidin-3-yl)-1,3-dioxoisoindolin-4-yl)piperazin-1-yl)-2-oxoethyl)hydrazineyl)thiazol-4-yl)benzonitrile (TP21, NP1161)

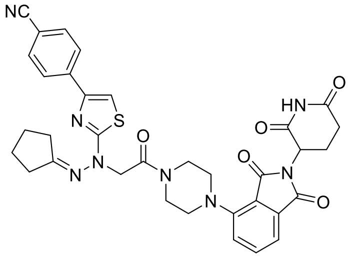

^{1}H NMR (400 MHz, DMSO-d_6) δ 11.11 (s, 1H), 7.99 (d, J = 8.9 Hz, 2H), 7.80 (d, J = 8.4 Hz, 2H), 7.74 (d, J = 7.6 Hz, 1H), 7.48 (s, 1H), 7.42 (d, J = 7.0 Hz, 1H), 7.37 (d, J = 8.8 Hz, 1H), 5.18 (d, J = 5.7 Hz, 1H), 5.15–5.07 (m, 1H), 4.69 (d, J = 12.5 Hz, 1H), 3.70 (d, J = 23.6 Hz, 4H), 3.52 (d, J = 33.2 Hz, 3H), 3.39 (s, 5H), 3.28 (s, 3H), 2.95–2.81 (m, 1H), 2.66–2.53 (m, 2H), 2.46 (s, 1H), 2.04 (dd, J = 12.6, 6.1 Hz, 1H). ^{13}C NMR (101 MHz, Chloroform-d) δ 176.4, 175.1, 171.1, 168.3, 167.1, 167.0, 166.6, 149.7, 149.6, 140.6, 135.9, 132.3, 126.2, 123.3, 116.7, 110.3, 107.7, 54.2, 51.7, 49.2, 41.7, 34.8, 31.4, 30.1, 29.6, 29.3, 22.6. RT = 14.89 min.

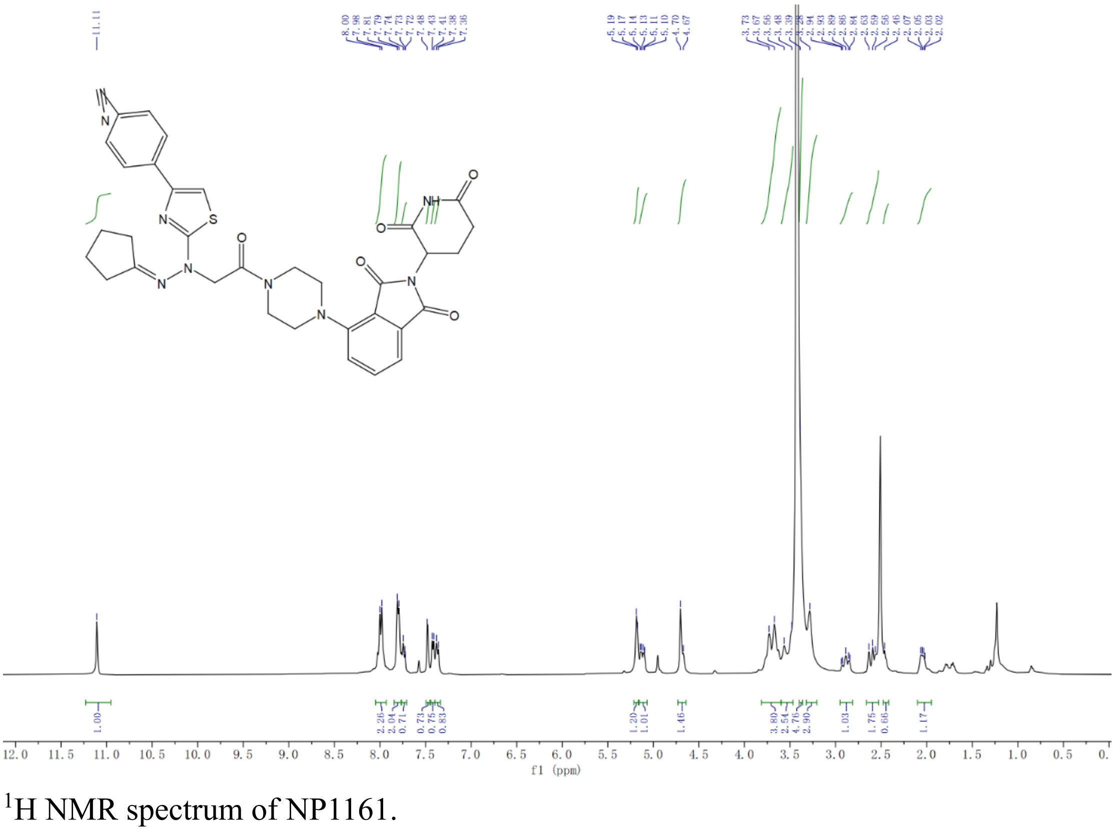

^{1}H NMR spectrum of NP1161.

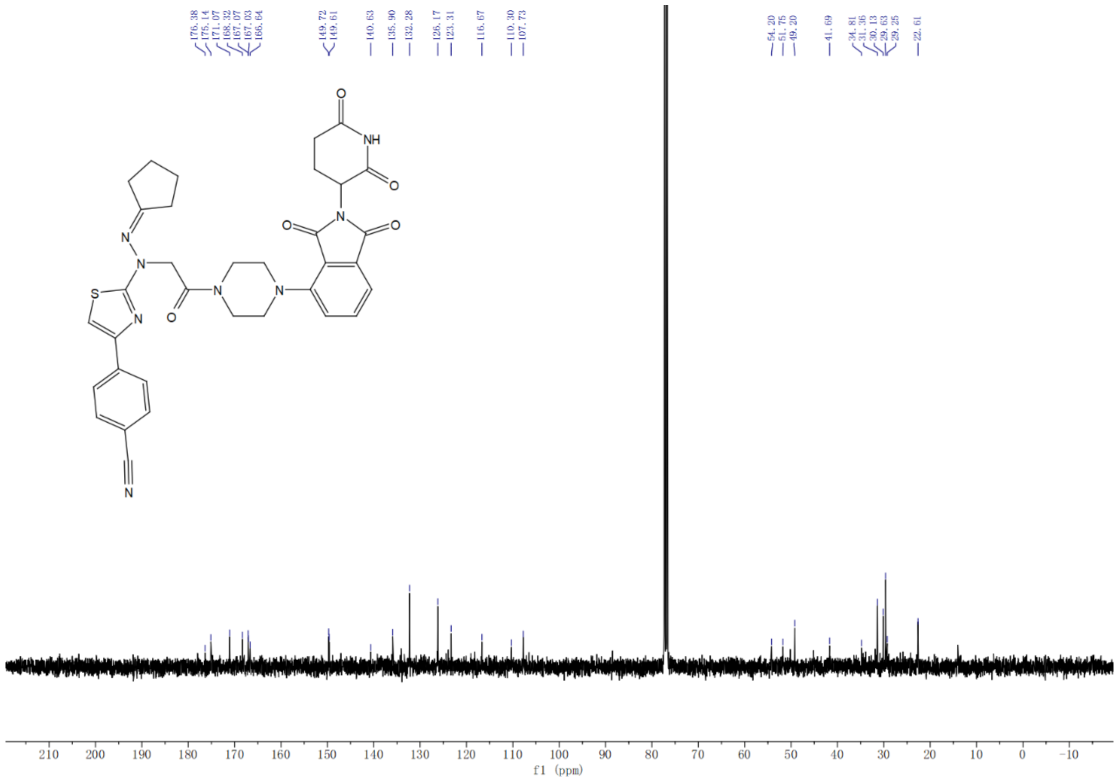

^{13}C NMR spectrum of compound NP1161.

4-(2-(2-Cyclopentylidene-1-(4-(4-(2-(2,6-dioxopiperidin-3-yl)-1,3-dioxoisoindolin-4-yl)piperazin-1-yl)-4-oxobutyl)hydrazineyl)thiazol-4-yl)benzonitrile (TP22)

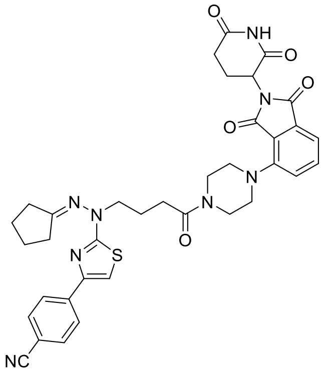

^{1}H NMR (400 MHz, DMSO-d_6) δ 11.09 (s, 1H), 7.96 (d, J = 7.8 Hz, 2H), 7.78 (d, J = 8.4 Hz, 2H), 7.66 (t, J = 8.0 Hz, 1H), 7.37 (t, J = 5.4 Hz, 2H), 7.12 (d, J = 8.4 Hz, 1H), 5.19 (d, J = 9.5 Hz, 2H), 5.11 (d, J = 7.8 Hz, 1H), 3.73 (d, J = 7.3 Hz, 2H), 3.59 (d, J = 27.7 Hz, 4H), 3.33 (s, 4H), 3.14 (s, 4H), 2.88 (t, J = 14.9 Hz, 1H), 2.65–2.52 (m, 2H), 2.46 (t, J = 7.2 Hz, 3H), 2.09–1.89 (m, 4H). ^{13}C NMR (101 MHz, Chloroform-d) δ 180.2, 178.0, 175.8, 175.1, 172.2, 171.4, 154.5, 154.0, 144.3, 138.6, 137.6, 131.1, 124.3, 121.9, 114.2, 112.2, 57.9, 54.0, 36.3, 36.1, 35.0, 34.7, 27.2, 27.2, 26.3. RT = 15.37 min.

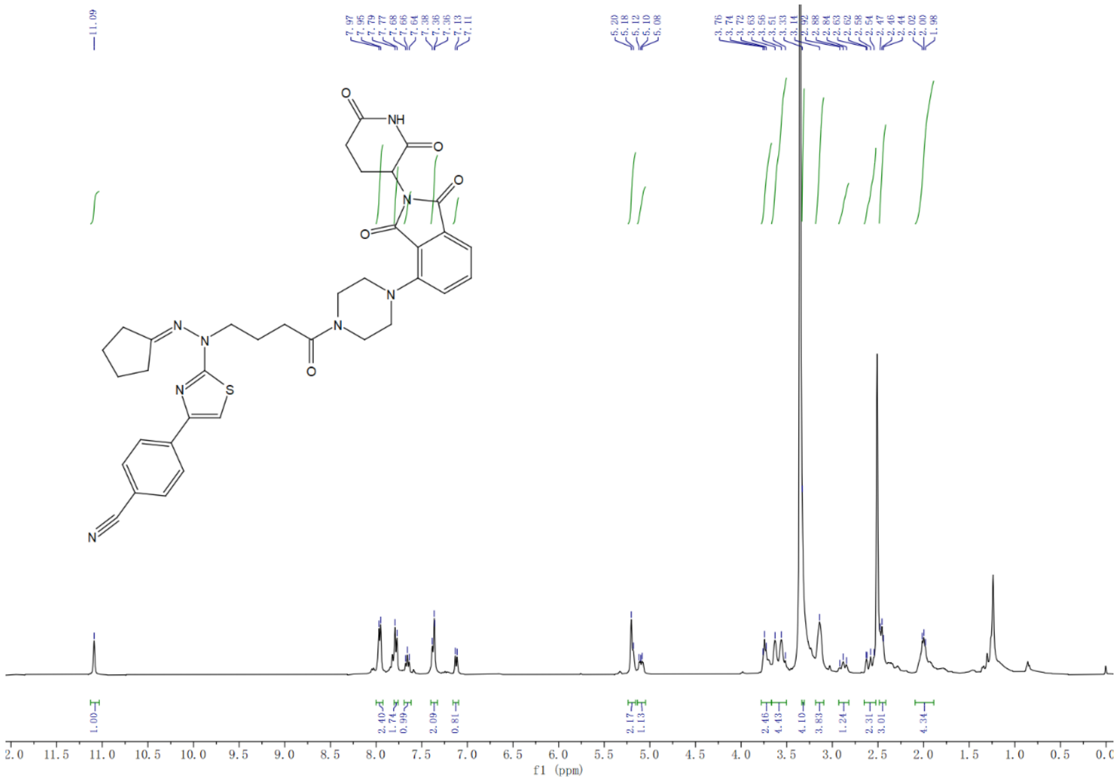

^{1}H NMR spectrum of compound TP22.

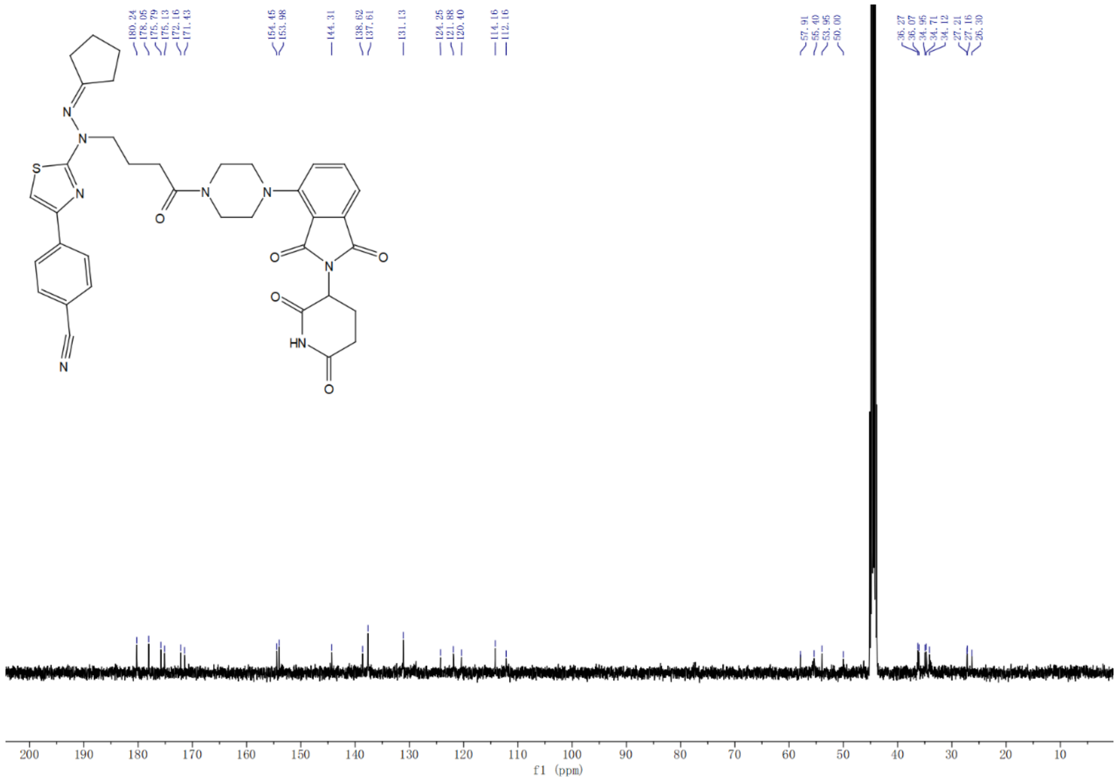

^{13}C NMR spectrum of compound TP22.

4-(2-(2-Cyclopentylidene-1-(5-(4-(2-(2,6-dioxopiperidin-3-yl)-1,3-dioxoisoindolin-4-yl)piperazin-1-yl)-5-oxopentyl)hydrazineyl)thiazol-4-yl)benzonitrile (TP23)

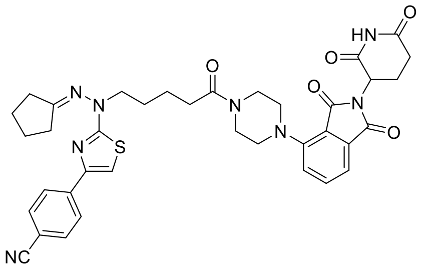

^{1}H NMR (400 MHz, Chloroform-*d*) δ 8.26 (s, 1H), 7.96 (d, *J* = 8.1 Hz, 2H), 7.68 (d, *J* = 8.2 Hz, 2H), 7.63 (d, *J* = 8.0 Hz, 1H), 7.47 (d, *J* = 7.2 Hz, 1H), 7.12 (d, *J* = 8.4 Hz, 1H), 6.99 (s, 1H), 5.03 (dd, *J* = 11.9, 5.4 Hz, 1H), 3.99 (t, *J* = 6.8 Hz, 2H), 3.83 (t, *J* = 7.7 Hz, 2H), 3.64 (t, *J* = 5.1 Hz, 2H), 3.27 (dq, *J* = 10.7, 6.2, 5.3 Hz, 4H), 2.96–2.76 (m, 3H), 2.65–2.57 (m, 2H), 2.47 (dt, *J* = 14.7, 6.8 Hz, 4H), 2.20–2.11 (m, 1H), 1.87 (dd, *J* = 8.7, 5.4 Hz, 4H), 1.76 (dd, *J* = 13.7, 6.5 Hz, 4H). ^{13}C NMR (101 MHz, Chloroform-*d*) δ 182.5, 172.3, 171.3, 170.9, 168.2, 167.1, 166.6, 149.8, 149.7, 135.8, 134.1, 132.4, 126.2, 123.3, 118.0, 116.4, 110.4, 106.7, 52.1, 52.0, 50.1, 49.2, 45.6, 41.3, 33.8, 32.7, 31.5, 31.3, 26.2, 25.0, 24.2, 22.6, 22.3. RT = 15.82 min.

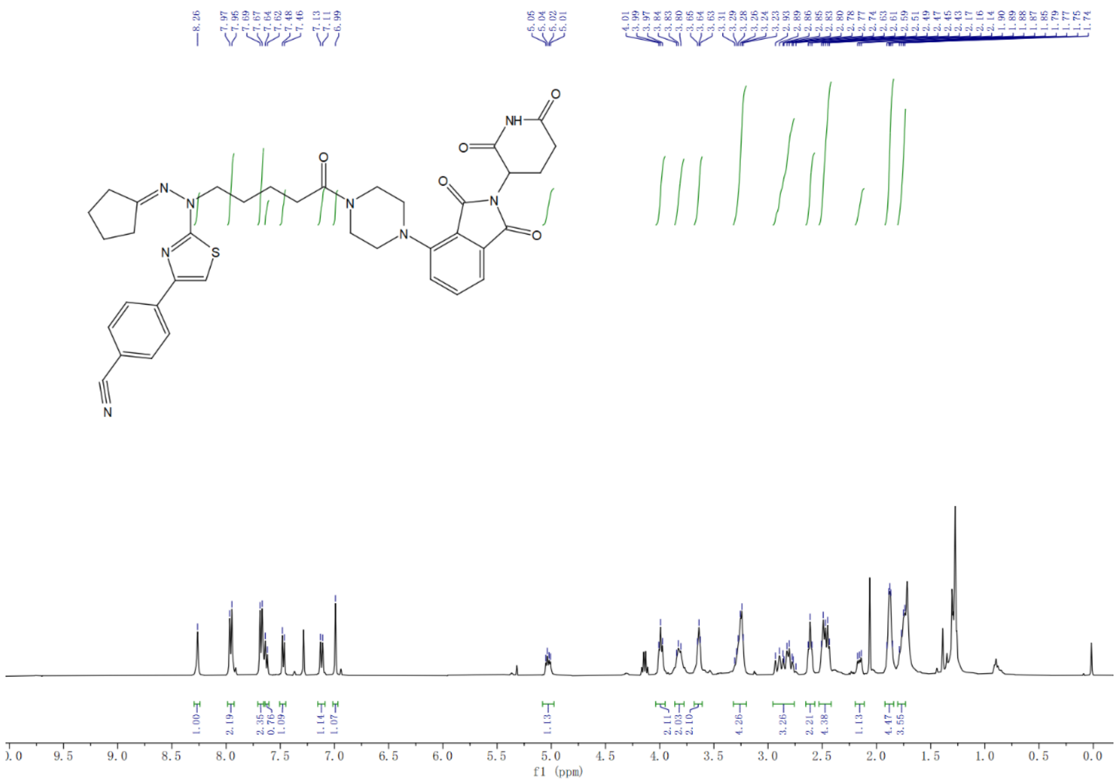

^{1}H NMR spectrum of compound TP23.

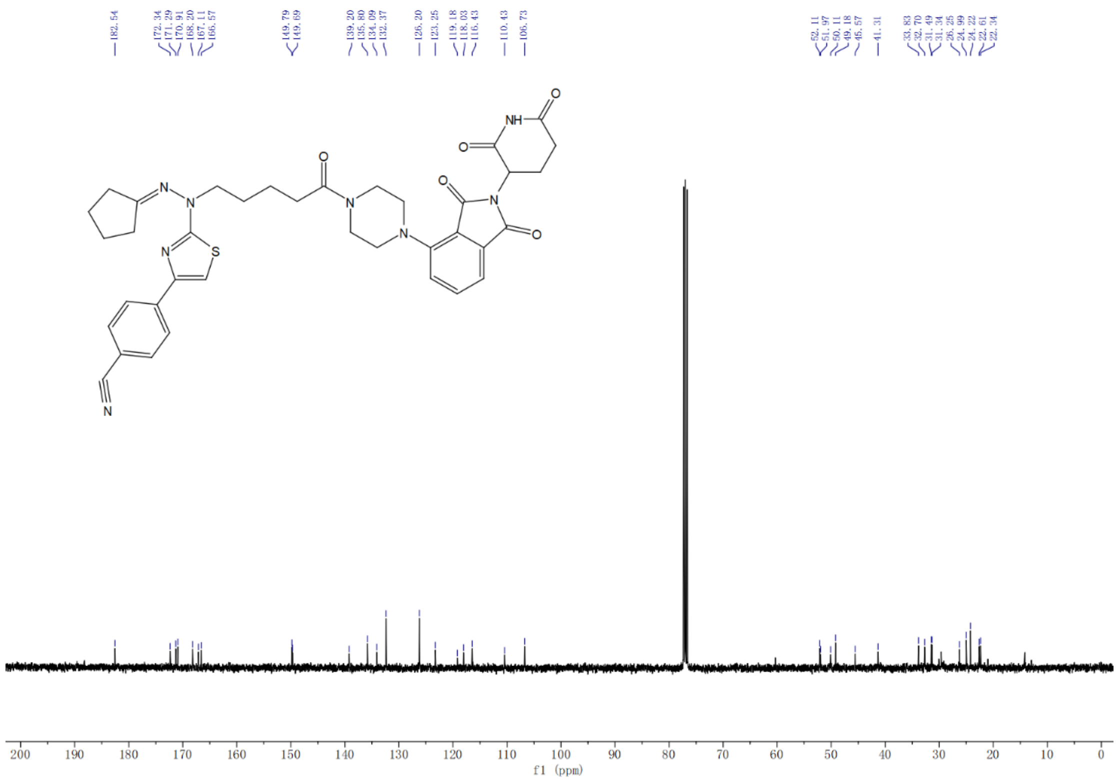

^{13}C NMR spectrum of compound TP23.

General procedure for the synthesis of compounds **TP25–32**

These compounds were prepared in a similar manner as TP21–TP24 but with intermediates 31–32 replacing intermediate 22 to obtain a yellow solid (31.3-44.9% yield).

4-(2-(2-Cyclopentylidene-1-(2-(4-(1-(2-(2,6-dioxopiperidin-3-yl)-1,3-dioxoisoindolin-4-yl)piperidin-4-yl)piperazin-1-yl)-2-oxoethyl)hydrazineyl)thiazol-4-yl)benzonitrile (TP25, NP1163)

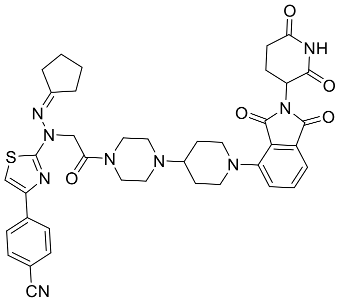

^1H NMR (400 MHz, Chloroform-*d*) δ 8.68 (s, 1H), 7.87 (d, *J* = 8.2 Hz, 2H), 7.65 (d, *J* = 8.0 Hz, 2H), 7.59 (t, *J* = 8.0 Hz, 1H), 7.40 (d, *J* = 7.2 Hz, 1H), 7.18 (d, *J* = 8.4 Hz, 1H), 7.00 (s, 1H), 5.02 (dd, *J* = 12.2, 5.3 Hz, 1H), 4.75 (s, 2H), 4.63 (s, 2H), 3.82 (t, *J* = 12.0 Hz, 2H), 3.65 (s, 2H), 3.55 (d, *J* = 4.9 Hz, 2H), 2.85 (dp, *J* = 37.0, 13.7, 12.5 Hz, 6H), 2.70 (d, *J* = 6.2 Hz, 2H), 2.63 (d, *J* = 5.4 Hz, 2H), 2.53 (d, *J* = 11.7 Hz, 1H), 2.18–2.10 (m, 1H), 1.92 (d, *J* = 11.2 Hz, 6H), 1.86–1.75 (m, 3H). ^{13}C NMR (101 MHz, DMSO-d_6) δ 175.4, 173.2, 170.4, 167.5, 166.8, 166.6, 150.2, 149.2, 139.5, 136.2, 134.0, 132.9, 126.5, 124.3, 119.5, 116.8, 115.0, 109.6, 108.5, 60.9, 54.1, 50.8, 49.2, 49.0, 45.0,

42.1, 31.4, 29.4, 28.2, 22.5. RT = 13.31 min.

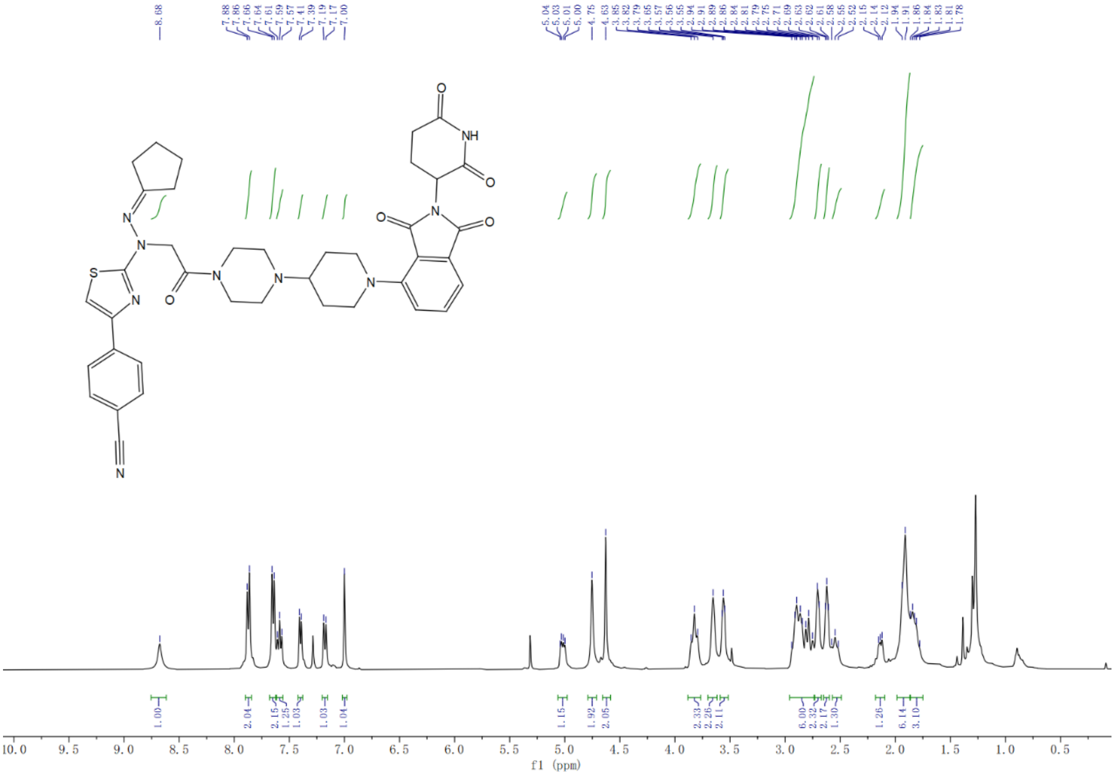

^{1}H NMR spectrum of compound NP1163.

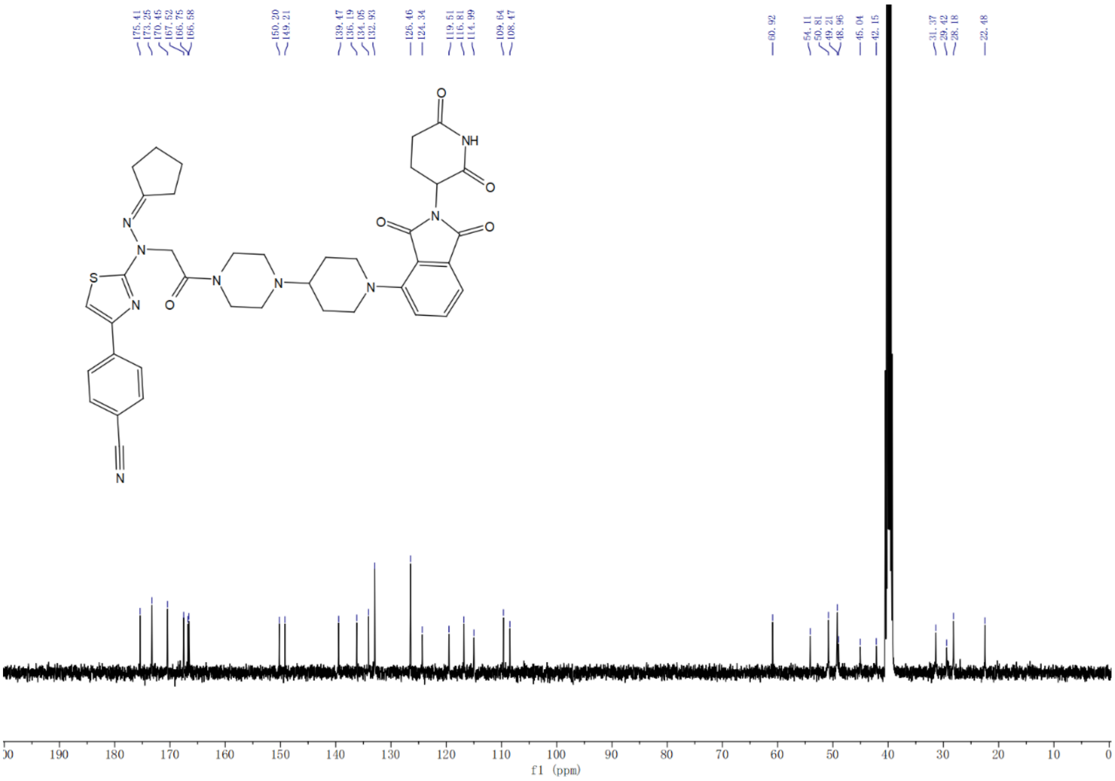

^{13}C NMR spectrum of compound NP1163.

4-(2-(2-Cyclopentylidene-1-(4-(4-(1-(2-(2,6-dioxopiperidin-3-yl)-1,3-dioxoisoindolin-4-yl)piperidin-4-yl)piperazin-1-yl)-4-

oxobutyl)hydrazineyl)thiazol-4-yl)benzonitrile (TP26)

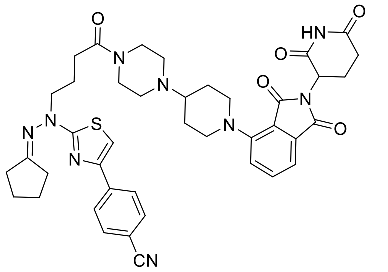

^{1}H NMR (400 MHz, DMSO-d_6) δ 11.10 (s, 1H), 7.99 (d, *J* = 7.9 Hz, 2H), 7.80 (d, *J* = 7.9 Hz, 2H), 7.68 (t, *J* = 8.0 Hz, 1H), 7.38 (s, 1H), 7.31 (dd, *J* = 12.3, 7.7 Hz, 2H), 5.17 (s, 2H), 5.09 (dd, *J* = 13.2, 5.3 Hz, 1H), 3.76–3.62 (m, 4H), 3.33 (s, 6H), 2.81 (ddt, *J* = 49.4, 25.8, 14.9 Hz, 6H), 2.65–2.54 (m, 2H), 2.46 (d, *J* = 8.9 Hz, 2H), 2.36 (p, *J* = 5.4, 4.0 Hz, 5H), 2.28 (d, *J* = 12.7 Hz, 4H), 2.00 (dt, *J* = 27.9, 7.0 Hz, 4H). ^{13}C NMR (101 MHz, Chloroform-*d*) δ 171.1, 170.6, 168.4, 168.3, 167.3, 166.6, 150.2, 149.6, 139.4, 135.5, 134.1, 132.4, 126.0, 124.4, 123.5, 117.3, 115.6, 110.2, 106.2, 61.3, 53.8, 51.2, 49.1, 48.7, 34.8, 31.9, 31.4, 30.4, 30.1, 29.9, 29.6, 29.3, 28.0, 22.6, 14.1. RT = 15.74 min.

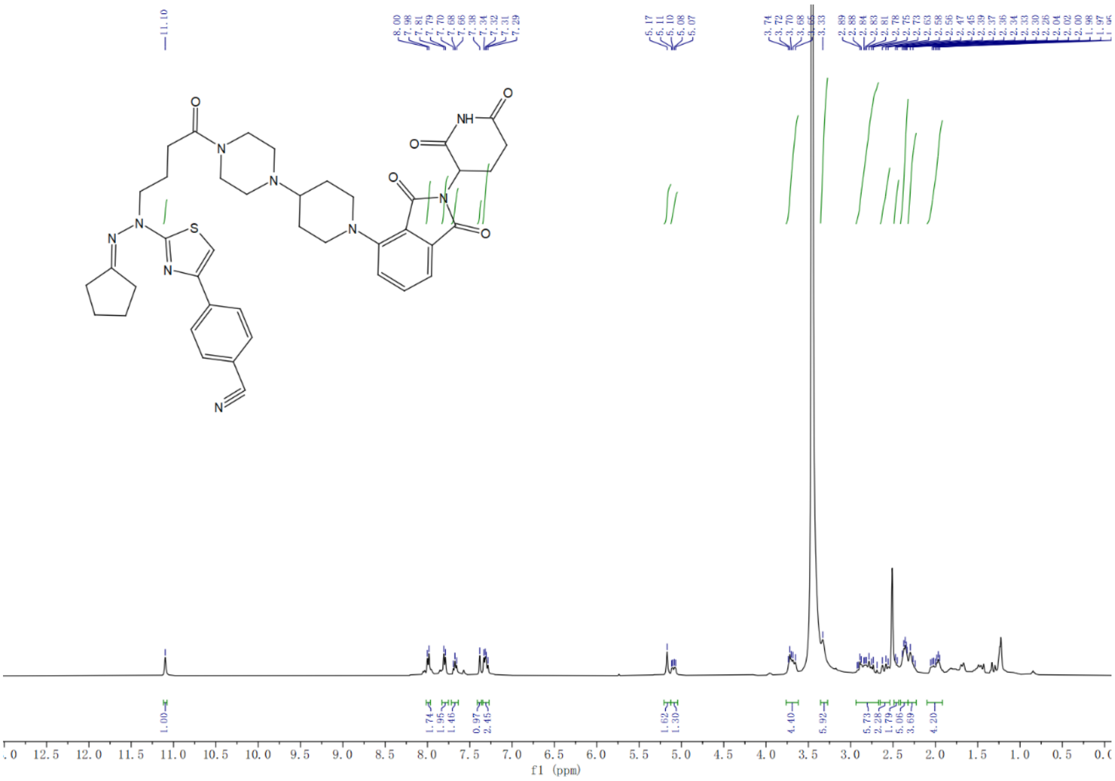

^{1}H NMR spectrum of compound TP26.

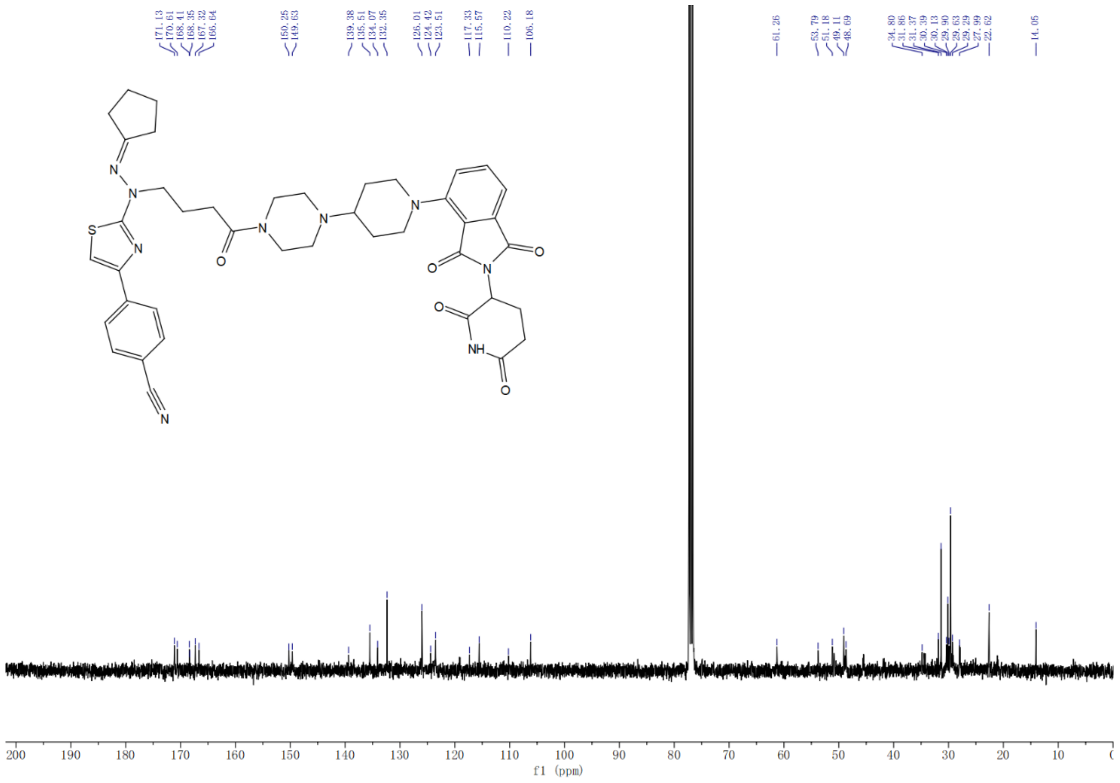

^{13}C NMR spectrum of compound TP26.

4-(2-(2-Cyclopentylidene-1-(5-(4-(1-(2-(2,6-dioxopiperidin-3-yl)-1,3-dioxoisoindolin-4-yl)piperidin-4-yl)piperazin-1-yl)-5-oxopentyl)hydrazineyl)thiazol-4-yl)benzonitrile (TP27)

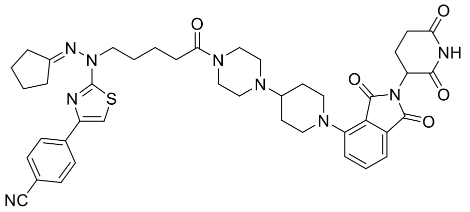

^{1}H NMR (400 MHz, Chloroform-*d*) δ 8.60 (s, 1H), 7.95 (d, *J* = 8.1 Hz, 2H), 7.66 (d, *J* = 8.1 Hz, 2H), 7.58 (t, *J* = 7.9 Hz, 1H), 7.39 (d, *J* = 7.5 Hz, 1H), 7.17 (d, *J* = 8.5 Hz, 1H), 6.99 (s, 1H), 5.08–4.90 (m, 1H), 3.97 (t, *J* = 6.8 Hz, 2H), 3.80 (t, *J* = 10.1 Hz, 2H), 3.61 (s, 2H), 3.40 (s, 2H), 2.84 (dq, *J* = 33.4, 17.7, 14.2 Hz, 6H), 2.53 (dd, *J* = 36.4, 14.1 Hz, 11H), 2.37 (t, *J* = 6.9 Hz, 3H), 2.12 (d, *J* = 11.3 Hz, 1H), 1.96 (s, 2H), 1.79–1.66 (m, 6H). ^{13}C NMR (101 MHz, Chloroform-*d*) δ 182.6, 172.3, 171.1, 171.1, 168.3, 167.3, 166.6, 150.3, 149.7, 139.2, 135.5, 134.0, 132.4, 126.2, 123.6, 119.1, 117.3, 115.5, 110.4, 106.7, 61.3, 52.3, 51.2, 50.9, 49.1, 49.0, 45.8, 41.7, 33.8, 32.8, 31.5, 31.4, 28.0, 26.4, 25.0, 24.2, 22.6. RT = 16.08 min.

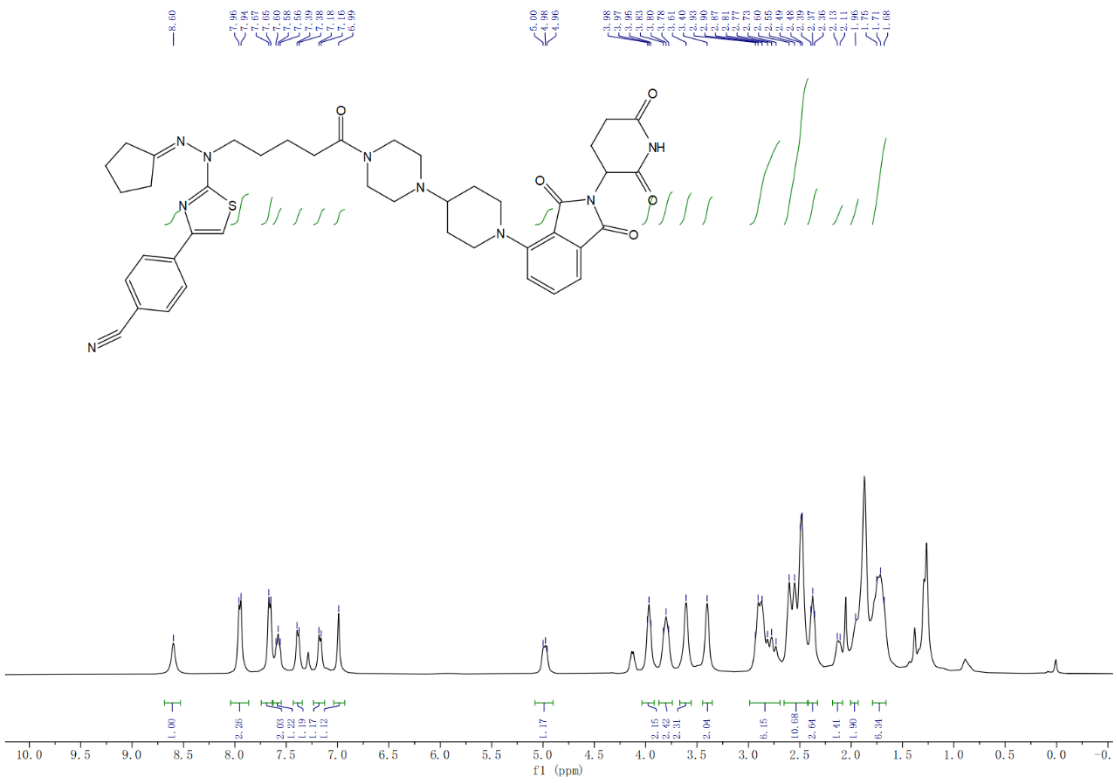

^{1}H NMR spectrum of compound TP27.

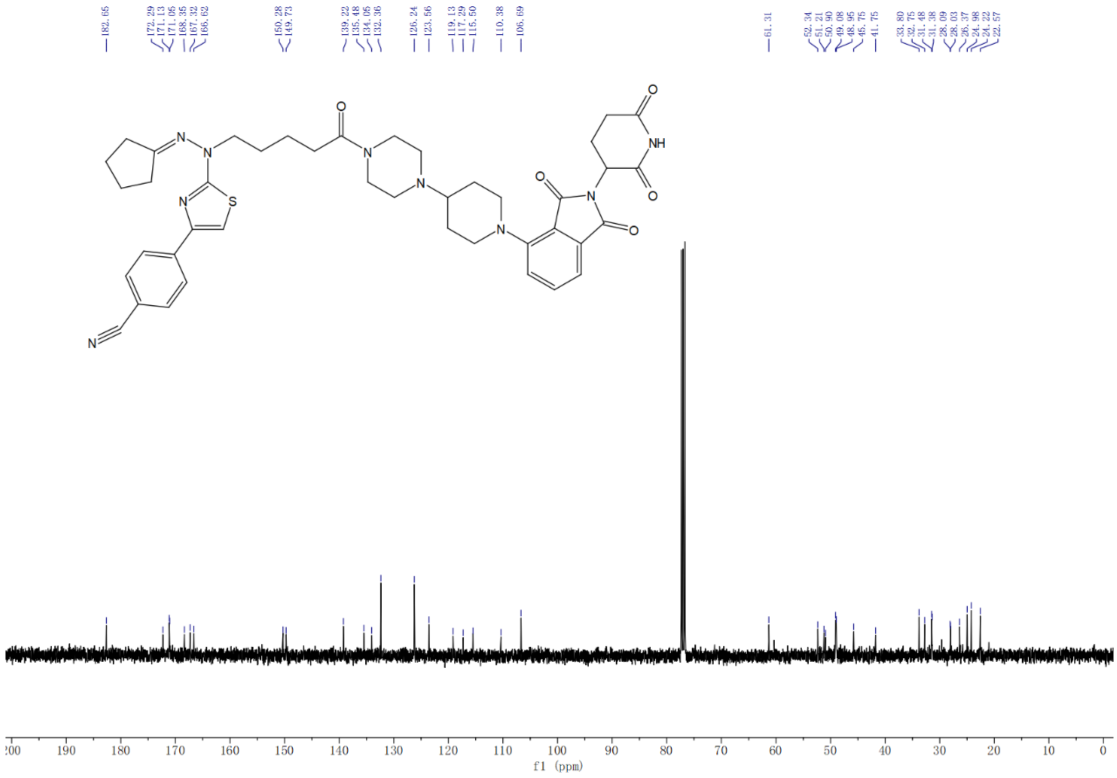

^{13}C NMR spectrum of compound TP27.

4-(2-(2-Cyclopentylidene-1-(7-(4-(1-(2-(2,6-dioxopiperidin-3-yl)-1,3-dioxoisoindolin-4-yl)piperidin-4-yl)piperazin-1-yl)-7-oxoheptyl)hydrazineyl)thiazol-4-yl)benzonitrile (TP28)

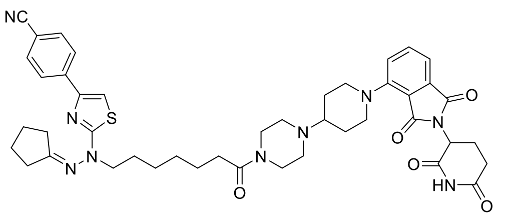

^{1}H NMR (400 MHz, Chloroform-*d*) δ 8.47 (s, 1H), 7.92 (d, *J* = 8.4 Hz, 2H), 7.65 (d, *J* = 8.4 Hz, 2H), 7.61–7.55 (m, 1H), 7.39 (d, *J* = 7.1 Hz, 1H), 7.17 (d, *J* = 8.5 Hz, 1H), 6.94 (s, 1H), 4.98 (dd, *J* = 12.1, 5.4 Hz, 1H), 4.21 (s, 2H), 3.80 (t, *J* = 9.1 Hz, 2H), 3.74 (t, *J* = 7.2 Hz, 2H), 3.64 (t, *J* = 5.0 Hz, 2H), 3.47 (t, *J* = 5.8 Hz, 2H), 2.97–2.70 (m, 6H), 2.59 (t, *J* = 5.0 Hz, 4H), 2.51 (dq, *J* = 11.1, 5.9, 4.2 Hz, 2H), 2.33 (t, *J* = 7.5 Hz, 2H), 2.16–2.09 (m, 1H), 1.93 (d, *J* = 10.8 Hz, 4H), 1.83–1.73 (m, 4H), 1.67 (t, *J* = 7.4 Hz, 3H), 1.49–1.41 (m, 5H). ^{13}C NMR (101 MHz, Chloroform-*d*) δ 174.6, 171.4, 171.0, 168.3, 167.3, 166.6, 150.3, 149.9, 139.3, 135.5, 134.1, 132.3, 126.2, 123.6, 119.2, 117.3, 115.5, 110.3, 106.2, 61.4, 54.2, 51.2, 50.9, 49.1, 48.9, 45.8, 41.8, 32.9, 31.4, 29.6, 29.0, 28.1, 26.3, 25.8, 25.0, 22.6. RT = 17.26 min.

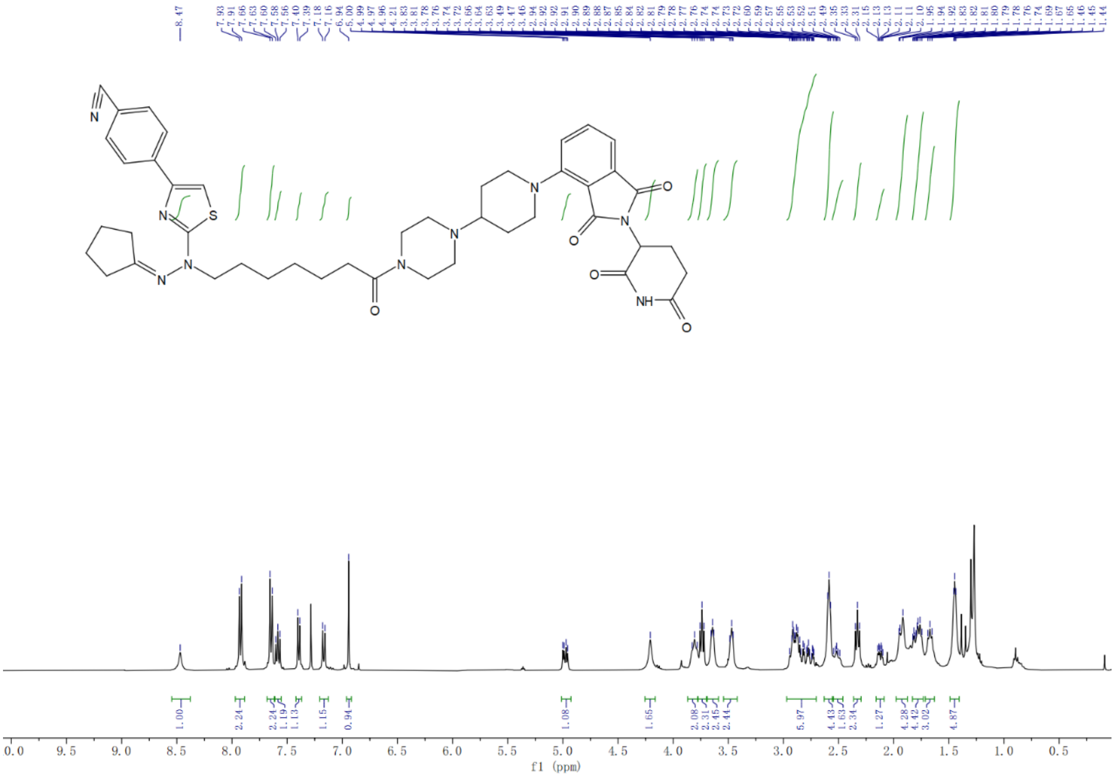

^{1}H NMR spectrum of compound TP28.

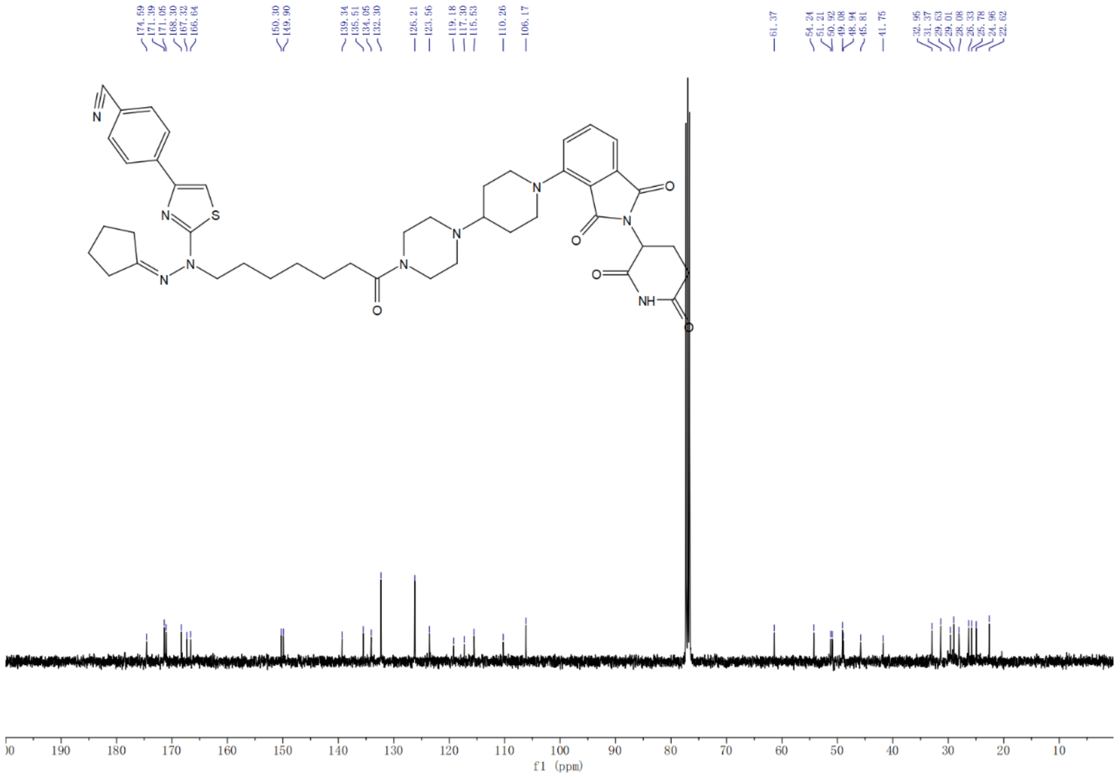

^{13}C NMR spectrum of compound TP28.

4-(2-(2-Cyclopentylidene-1-(2-(4-((1-(2-(2,6-dioxopiperidin-3-yl)-1,3-dioxoisoindolin-4-yl)piperidin-4-yl)methyl)piperazin-1-yl)-2-oxoethyl)hydrazineyl)thiazol-4-yl)benzonitrile (TP29)

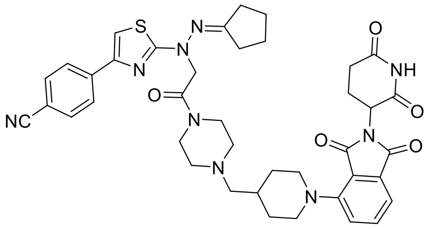

^{1}H NMR (400 MHz, Chloroform-*d*) δ 8.41 (s, 1H), 7.88 (d, *J* = 7.7 Hz, 2H), 7.64 (d, *J* = 7.7 Hz, 2H), 7.58 (q, *J* = 6.8, 5.7 Hz, 1H), 7.38 (d, *J* = 7.0 Hz, 1H), 7.19 (d, *J* = 8.3 Hz, 1H), 7.01 (d, *J* = 5.9 Hz, 1H), 5.04–4.92 (m, 1H), 4.74 (s, 2H), 4.65 (d, *J* = 6.4 Hz, 2H), 3.76 (t, *J* = 10.2 Hz, 2H), 3.65 (s, 2H), 3.56 (s, 2H), 2.96–2.71 (m, 6H), 2.63 (dd, *J* = 11.1, 6.3 Hz, 1H), 2.53 (t, *J* = 5.3 Hz, 2H), 2.48–2.42 (m, 2H), 2.30 (d, *J* = 6.9 Hz, 3H), 2.12 (dd, *J* = 11.4, 5.5 Hz, 1H), 1.92 (d, *J* = 12.3 Hz, 3H), 1.72 (s, 2H), 1.49 (d, *J* = 13.7 Hz, 3H). ^{13}C NMR (101 MHz, Chloroform-*d*) δ 175.2, 171.1, 168.3, 167.4, 166.7, 166.6, 150.8, 149.7, 139.3, 135.5, 134.1, 132.3, 126.3, 126.2, 123.6, 119.2, 117.2, 115.3, 110.3, 107.7, 64.2, 54.2, 53.7, 53.0, 51.8, 51.5, 49.1, 44.9, 41.9, 33.0, 31.4, 31.4, 30.8, 30.1, 29.6, 24.2, 22.6. RT = 16.73 min.

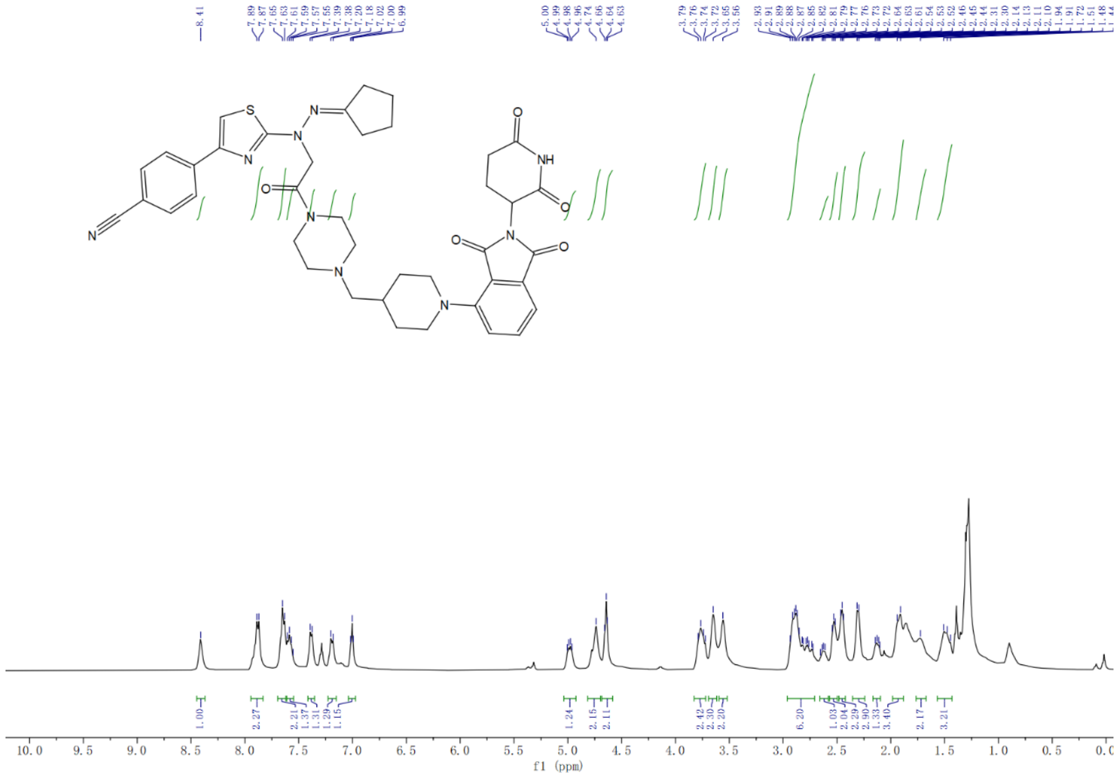

^{1}H NMR spectrum of compound TP29.

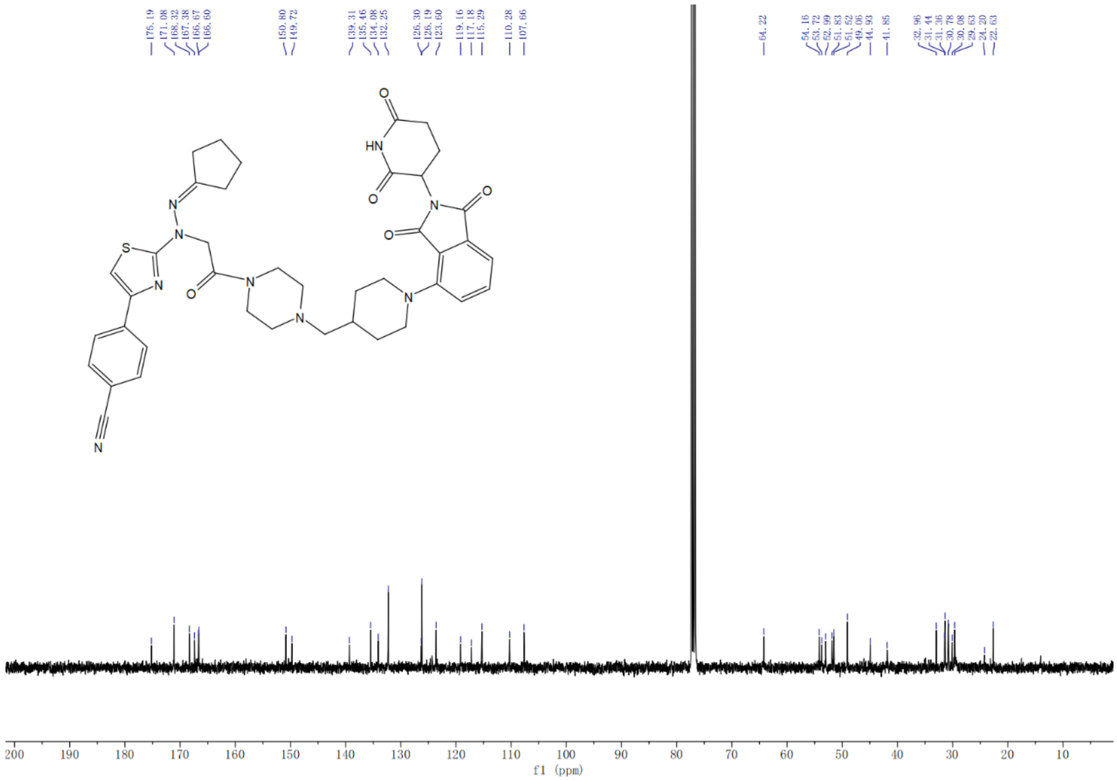

^{13}C NMR spectrum of compound TP29.

4-(2-(2-Cyclopentylidene-1-(4-(4-((1-(2-(2,6-dioxopiperidin-3-yl)-1,3-dioxoisoindolin-4-yl)piperidin-4-yl)methyl)piperazin-1-yl)-4-oxobutyl)hydrazineyl)thiazol-4-yl)benzonitrile (TP30)

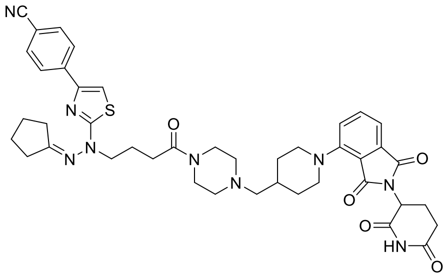

^{1}H NMR (400 MHz, Chloroform-*d*) δ 8.34 (s, 1H), 7.94 (dd, *J* = 15.8, 8.0 Hz, 2H), 7.66 (t, *J* = 7.0 Hz, 2H), 7.58 (t, *J* = 7.9 Hz, 1H), 7.38 (d, *J* = 7.1 Hz, 1H), 7.18 (t, *J* = 7.6 Hz, 1H), 7.00 (s, 1H), 4.98 (dd, *J* = 12.2, 5.4 Hz, 1H), 4.04 (t, *J* = 7.1 Hz, 2H), 3.75 (t, *J* = 11.2 Hz, 2H), 3.56 (q, *J* = 6.7, 6.0 Hz, 2H), 3.35 (dt, *J* = 17.9, 5.0 Hz, 2H), 2.94–2.84 (m, 3H), 2.83–2.71 (m, 2H), 2.64–2.56 (m, 2H), 2.51 (d, *J* = 7.2 Hz, 2H), 2.47 (t, *J* = 6.2 Hz, 1H), 2.38 (t, *J* = 7.1 Hz, 2H), 2.32 (d, *J* = 5.7 Hz, 3H), 2.26 (t, *J* = 5.1 Hz, 1H), 2.20 (d, *J* = 7.1 Hz, 2H), 2.16 (d, *J* = 9.3 Hz, 1H), 2.09 (t, *J* = 6.5 Hz, 1H), 2.04 (t, *J* = 7.2 Hz, 2H), 1.87 (s, 4H), 1.80 (d, *J* = 13.9 Hz, 3H). ^{13}C NMR (101 MHz, Chloroform-*d*) δ 182.0, 172.5, 171.1, 170.5, 168.3, 167.4, 166.7, 150.8, 149.7, 139.2, 135.4, 132.3, 126.3, 123.6, 119.1, 117.1, 115.2, 110.4, 106.7, 106.1, 64.2, 53.8, 53.0, 52.1, 51.8, 51.5, 49.0, 45.3, 41.5, 33.9, 32.9, 31.5, 31.4, 30.8, 30.3, 29.6, 25.0, 24.2, 22.6, 22.2. RT = 18.96 min.

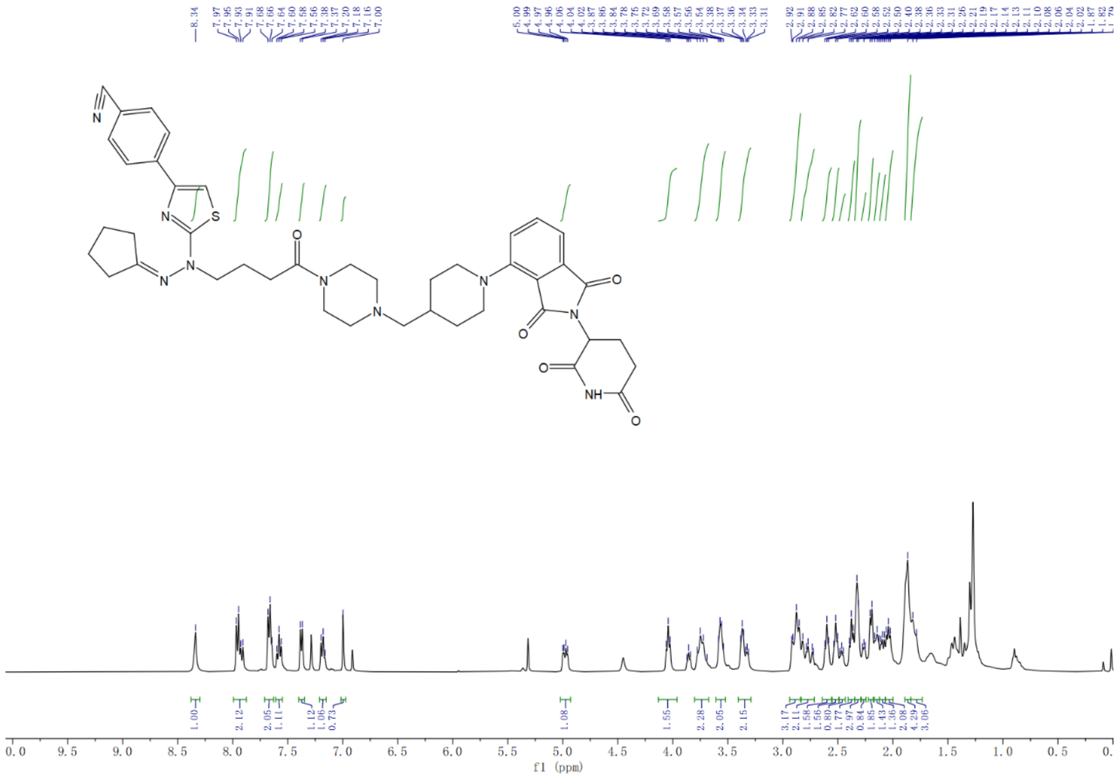

^{1}H NMR spectrum of compound TP30.

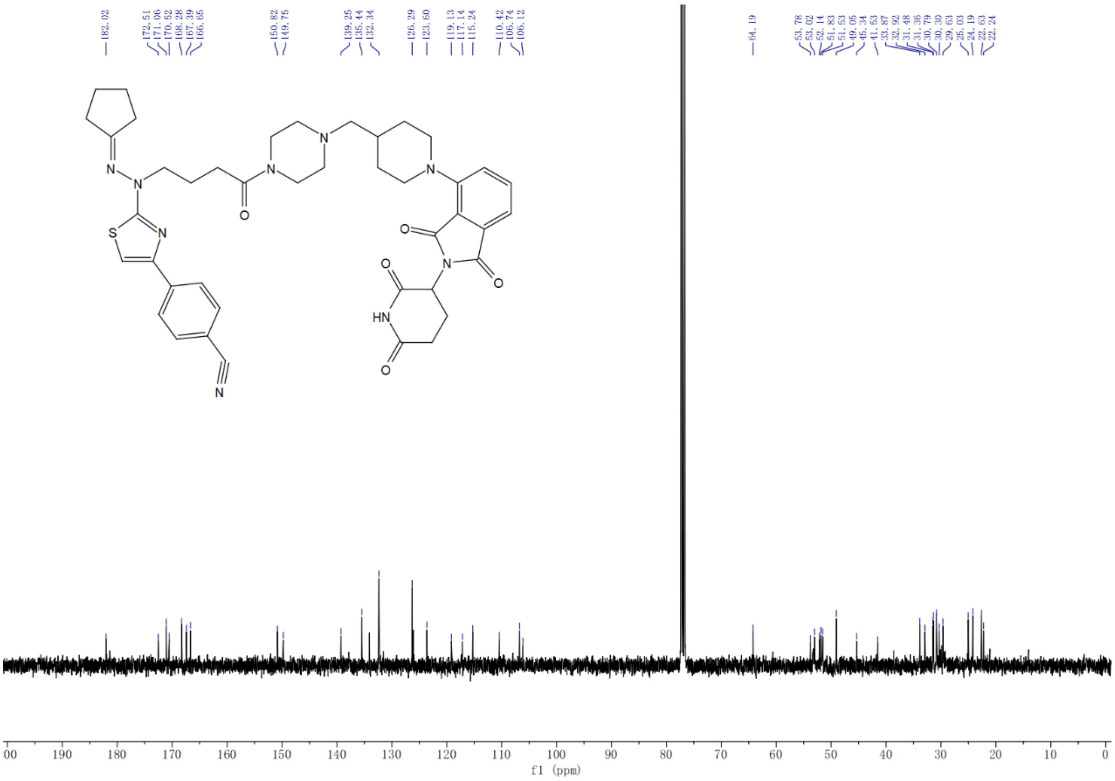

^{13}C NMR spectrum of compound TP30.

4-(2-(2-Cyclopentylidene-1-(5-(4-((1-(2-(2,6-dioxopiperidin-3-yl)-1,3-dioxoisoindolin-4-yl)piperidin-4-yl)methyl)piperazin-1-yl)-5-oxopentyl)hydrazineyl)thiazol-4-yl)benzonitrile (TP31)

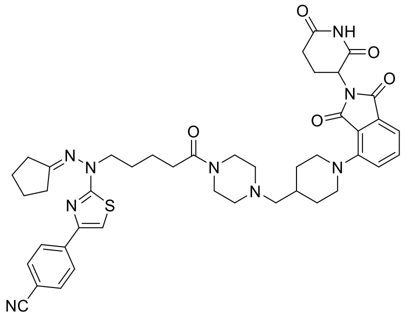

^{1}H NMR (400 MHz, Chloroform-*d*) δ 8.43 (s, 1H), 7.95 (d, *J* = 8.1 Hz, 2H), 7.67 (d, *J* = 8.0 Hz, 2H), 7.57 (t, *J* = 7.8 Hz, 1H), 7.37 (d, *J* = 7.0 Hz, 1H), 7.18 (d, *J* = 8.4 Hz, 1H), 6.99 (s, 1H), 4.98 (dd, *J* = 12.0, 5.4 Hz, 1H), 3.96 (t, *J* = 6.9 Hz, 2H), 3.75 (t, *J* = 10.5 Hz, 3H), 3.59 (d, *J* = 5.6 Hz, 2H), 3.46–3.37 (m, 2H), 2.94–2.68 (m, 6H), 2.66–2.57 (m, 2H), 2.47 (d, *J* = 7.1 Hz, 2H), 2.37 (q, *J* = 7.1, 6.7 Hz, 6H), 2.24 (d, *J* = 7.0 Hz, 2H), 2.16–2.08 (m, 1H), 1.91 (s, 2H), 1.77–1.64 (m, 6H), 1.47 (q, *J* = 8.4, 4.8 Hz, 3H). ^{13}C NMR (101 MHz, Chloroform-*d*) δ 182.8, 172.3, 171.1, 168.3, 167.4, 166.6, 150.8, 149.7, 139.2, 135.4, 134.1, 132.3, 126.3, 123.6, 119.1, 117.1, 115.2, 110.4, 106.7, 64.2, 53.9, 53.1, 52.5, 51.8, 51.5, 49.0, 41.5, 33.8, 32.9, 32.7, 31.5, 31.4, 30.8, 26.4, 25.0, 24.2, 22.6, 22.6. RT = 16.74 min.

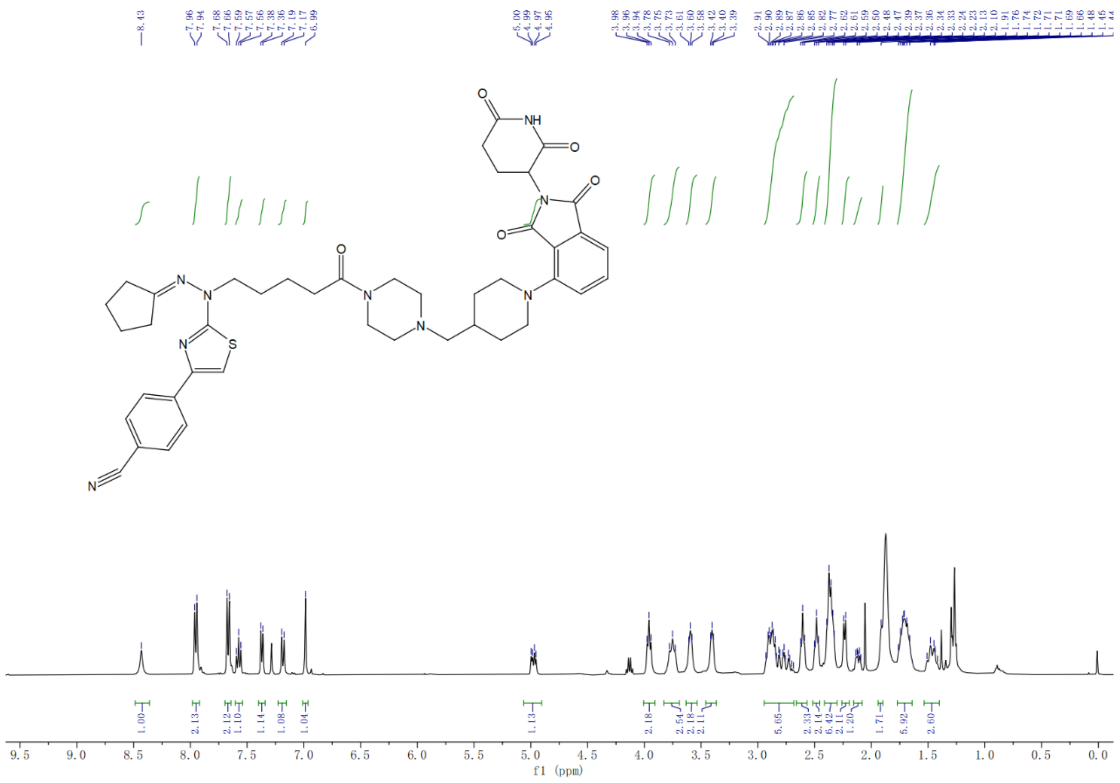

^{1}H NMR spectrum of compound TP31.

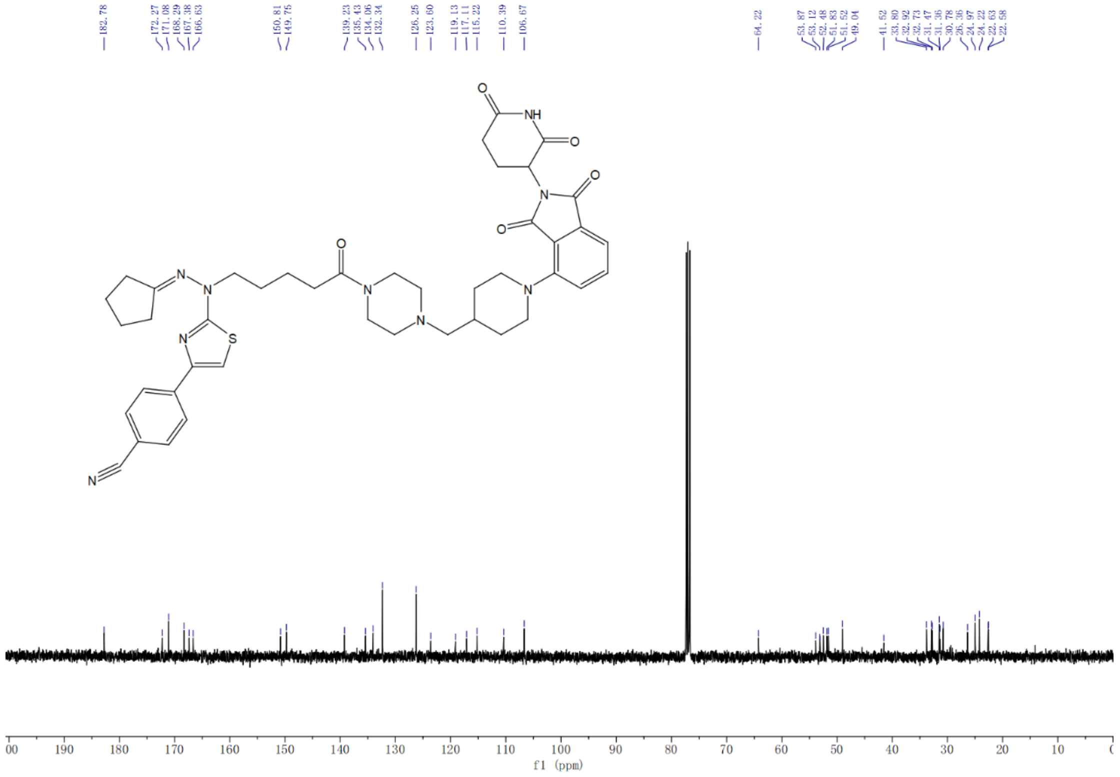

^{13}C NMR spectrum of compound TP31.

4-(2-(2-Cyclopentylidene-1-(7-(4-((1-(2-(2,6-dioxopiperidin-3-yl)-1,3-dioxoisoindolin-4-yl)piperidin-4-yl)methyl)piperazin-1-yl)-7-oxoheptyl)hydrazineyl)thiazol-4-yl)benzonitrile (TP32)

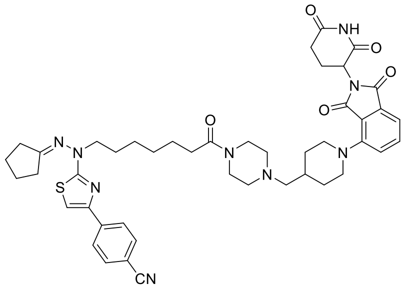

^{1}H NMR (400 MHz, Chloroform-*d*) δ 8.22 (s, 1H), 7.93 (d, *J* = 8.2 Hz, 2H), 7.66 (d, *J* = 8.2 Hz, 2H), 7.61–7.55 (m, 1H), 7.38 (d, *J* = 7.2 Hz, 1H), 7.19 (d, *J* = 8.4 Hz, 1H), 6.95 (s, 1H), 4.98 (dd, *J* = 12.1, 5.4 Hz, 1H), 4.19 (s, 2H), 3.79–3.71 (m, 4H), 3.62 (d, *J* = 5.4 Hz, 2H), 3.45 (t, *J* = 5.0 Hz, 2H), 3.00–2.84 (m, 4H), 2.84–2.70 (m, 2H), 2.40 (q, *J* = 4.9 Hz, 4H), 2.33 (t, *J* = 7.5 Hz, 2H), 2.27 (d, *J* = 7.2 Hz, 2H), 2.17–2.10 (m, 1H), 1.91 (dt, *J* = 13.2, 6.6 Hz, 3H), 1.81–1.74 (m, 4H), 1.72–1.64 (m, 4H), 1.51–1.42 (m, 7H). ^{13}C NMR (101 MHz, Chloroform-*d*) δ 174.6, 171.4, 171.0, 168.2, 167.4, 166.6, 150.8, 149.9, 139.3, 135.4, 134.1, 132.3, 126.2, 123.6, 119.2, 117.1, 115.2, 110.3, 106.1, 64.3, 54.3, 54.0, 53.1, 51.9, 51.5, 49.0, 45.5, 41.5, 33.0, 32.9, 31.4, 30.8, 29.6, 29.0, 26.4, 25.8, 25.0, 22.6. RT = 18.20 min.

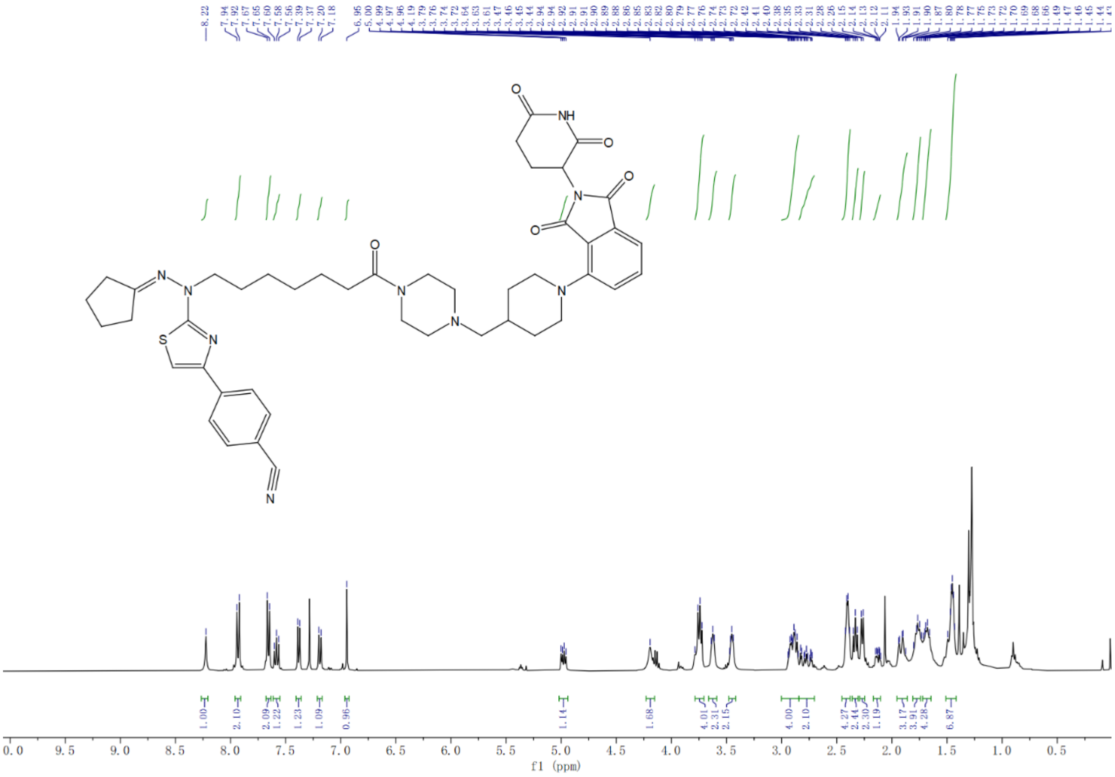

^{1}H NMR spectrum of compound TP32.

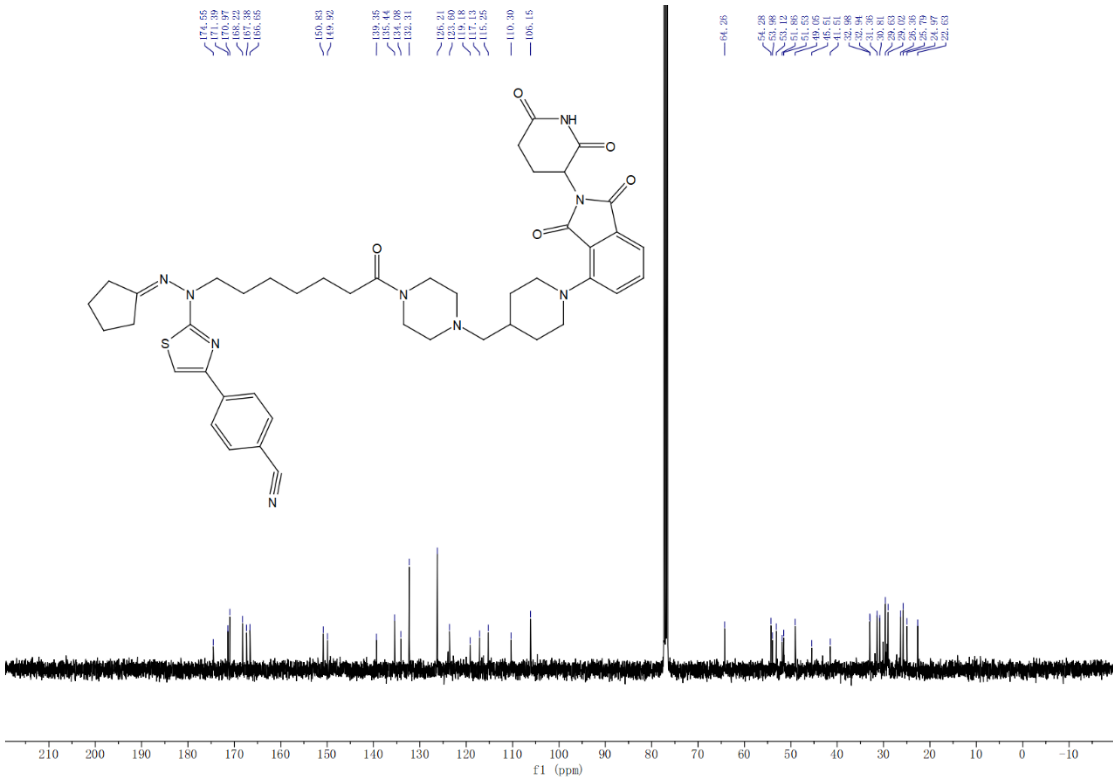

^{13}C NMR spectrum of compound TP32.
